# Improving the Efficacy of Quinolylnitrones for Ischemic Stroke Therapy, QN4 and QN15 as New Neuroprotective Agents after Oxygen–Glucose Deprivation/Reoxygenation-Induced Neuronal Injury

**DOI:** 10.3390/ph15111363

**Published:** 2022-11-07

**Authors:** José M. Alonso, Alejandro Escobar-Peso, Israel Fernández, Alberto Alcázar, José Marco-Contelles

**Affiliations:** 1 Laboratory of Medicinal Chemistry (IQOG, CSIC), C/Juan de la Cierva 3, 28006 Madrid, Spain; 2 Department of Research, Hospital Universitario Ramón y Cajal, IRYCIS, Ctra. Colmenar km 9.1, 28034 Madrid, Spain; 3 Departamento de Química Orgánica I and Centro de Innovación en Química Avanzada (ORFEO-CINQA), Facultad de Ciencias Químicas, Universidad Complutense de Madrid, 28040 Madrid, Spain

**Keywords:** brain ischemia, DFT calculations, ischemic stroke, neuroprotection, nitrones, quinolylnitrones, multipotent antioxidant drugs

## Abstract

In our search for new neuroprotective agents for stroke therapy to improve the pharmacological profile of the compound quinolylnitrone QN23, we have prepared and studied sixteen new, related and easily available quinolylnitrones. As a result, we have identified compounds QN4 and QN15 as promising candidates showing high neuroprotection power in a cellular experimental model of ischemia. Even though they were found to be less active than our current lead compound QN23, QN4 and QN15 provide an improved potency and, particularly for QN4, an expanded range of tolerability and improved solubility compared to the parent compound. A computational DFT-based analysis has been carried out to understand the antioxidant power of quinolylnitrones QN23, QN4 and QN15. Altogether, these results show that subtle, simple modifications of the quinolylnitrone scaffold are tolerated, providing high neuroprotective activity and optimization of the pharmacological potency required for an improved design and future drug developments in the field.

## 1. Introduction

Stroke is a pathology caused by the interruption of the blood supply to the brain, frequently of ischemic nature (87% of cases), mainly caused by a thrombus, but also due to the rupture of a blood vessel (hemorrhagic stroke) [1]. By cutting off the supply of oxygen and nutrients, stroke causes damage to the brain tissue [2]. A very severe stroke can cause sudden death, but it more likely constitutes a major cause of neurological disability, and the second cause of dementia after Alzheimer’s disease [3]. Restoration of the normal circulatory conditions may reverse the damage, but in case it is not allowed, compromised tissue may also turn into infarcted tissue [4]. Thus, a proper reperfusion should be the first goal for any ischemic stroke therapy, but other new therapies are sought to extend the benefit of reperfusion therapies, ameliorate the restoration of cellular homeostasis and improve neuroprotection against the damage caused by reperfusion itself, i.e., oxidative stress. This is the case of neuroprotection based on antioxidants such as nitrones, well-known organic molecules showing strong radical scavenging properties [5].

**Figure 1:**
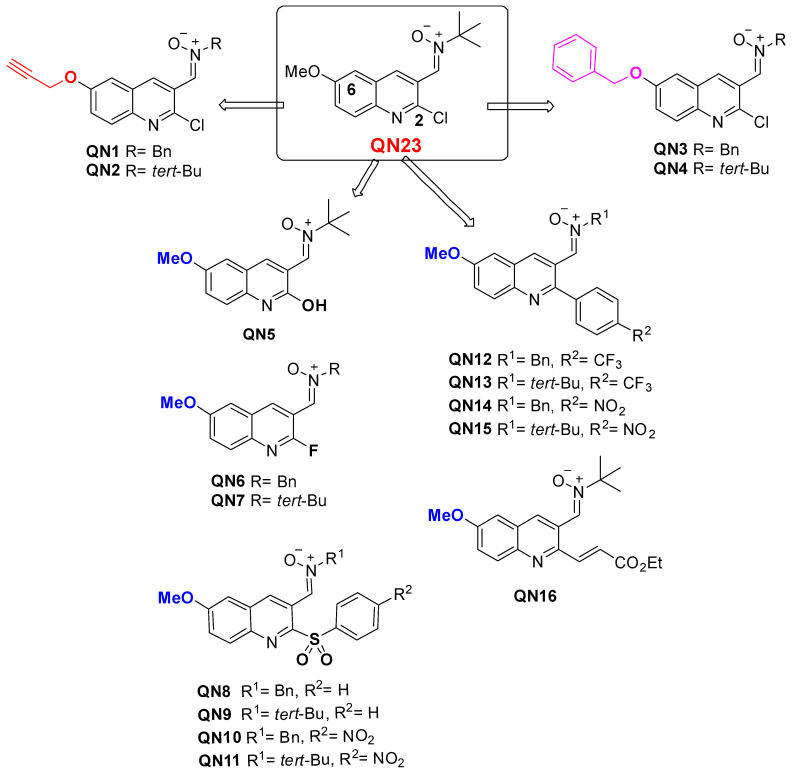
Structure of QN23 and the quinolylnitrones QNs 1–16 investigated in this work.

**Scheme 1:**
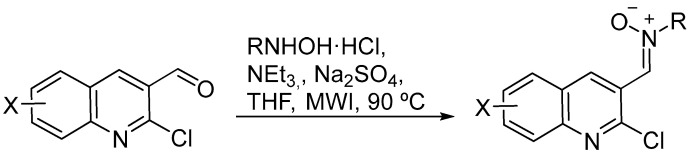
General method for the synthesis of QNs.

In this context, and based on a collaborative project with Dr. Alcázar’s (Hospital Ramón y Cajal, Madrid, Spain) and Prof. Oset-Gasque’s (UCM, Madrid, Spain) teams, in the last decade we have dedicated a great effort to develop an ambitious project targeted to the identification of novel small nitrones for the therapy of cerebral ischemia (CI) [6]. So far, we have reported new α-phenyl-*N-tert-*butyl nitrone analogues [7,8,9,10,11], *N*-substituted, *C*-dialkoxyphosphorylated nitrones [12], cholesteronitrones [13,14] and quinolylnitrones (QNs) [15,16,17,18]. Particularly, in the development of QNs, we have successfully demonstrated that (*Z*)-*N-tert*-butyl-1-(2-chloro-6-methoxyquinolin-3-yl)methanimine oxide (QN23) [15] (Figure 1) is a new and potent antioxidant agent, showing strong power to scavenge diverse radical oxygenated species (ROS) and efficient in vitro and in vivo neuroprotective profile [15,19]. QN23 (Figure 1) was synthesized following the general method shown in Scheme 1 from 2-chloro-6-methoxyquinoline-3-carbaldehyde and *N*-*tert*-butylhydroxylamine hydrochloride, in 82% yield, and isolated as a single and pure *Z*-isomer [15].

In a previous study, we reported the neuroprotection capacity of QN23, compared with the NXY-059 as reference nitrone, in primary neuronal cultures subjected to 4 h of oxygen and glucose deprivation (OGD) conditions, followed by treatment with the nitrones added at the beginning of a 24 h recovery period [15]. Neuronal cultures were treated with different concentrations of selected nitrones and their effect was evaluated after 24 h of recovery after OGD by a cell viability assay using 3-(4,5-dimethylthiazol-2-yl)-2,5-diphenyltetrazolium bromide (MTT). In these experiments, the cell viability without OGD was considered as 100% control, while 4 h OGD resulted in a significant reduction in cell viability (67.6%) compared with 100% control (*p* <0.0001, one-sample t-test). The OGD-induced cell injury was partially reversed after 24 h of recovery, but it did not reach the control value (77.1%; *p* < 0.001 compared with 4 h OGD by Student’s t-test). The reference neuroprotective nitrone NXY-059, assayed ranging from 1 μM to 1 mM, only produced effective neuroprotection at 250 μM (88.9%; *p* < 0.05 compared with 24 h of recovery by Dunnett’s post-test after ANOVA), while QN23 significantly increased cell viability at the whole 1−250 μM concentration range, reaching cell viabilities near to control values at 100 μM (94.9%; *p* < 0.01 compared with 24 h of recovery by Dunnett’s post-test after ANOVA) [15]. In Table 1, we have gathered and quantitatively compared the neuroprotective effect of QN23 (100−250 μM) and NXY-059, showing and confirming the remarkable neuroprotection provided by QN23 [15]. These results prompted us to start the in vivo CI model studies with this compound. The already-reported positive results have allowed us to propose ligand QN23 for advanced pre-clinical studies, as a promising candidate for the treatment of ischemic stroke [15]. Taking QN23 as a reference, we have kept focused on the development of new and improved quinolylnitrones derivatives in terms of neuroprotection activity. In this work, we shall describe the synthesis and the neuroprotective properties of sixteen novel QN23 analogues, in our attempt to keep researching on this privileged scaffold for the potential treatment of ischemic stroke. 

## 2. Results and Discussion

### 2.1. Design and Synthesis of New Quinolylnitrones

For the design and selection of the novel QNs, first of all we were curious about knowing the effect of the substitution of the *O*-methyl group at C6 by the *O*-propargyl and *O*-benzyl groups, including in both cases the benzyl (Bn) and the *tert-*butyl (*tert*-Bu) as the *N*-substituent at the nitrone functional motif, a process that resulted in QNs 1–4 (Figure 1). Next, we explored the replacement of the chlorine atom at C2 by: (a) a hydroxyl group, hence QN5; (b) a fluorine atom, resulting in QNs 6,7; (c) arylsulfones such as in QNs 8–11; and (d) a carbon atom implying an aryl group such as those in QNs 12–15; (Figure 1) or an ethyl α,β-unsaturated carboxylic ester, QN16 (Figure 1).

**Scheme 2:**
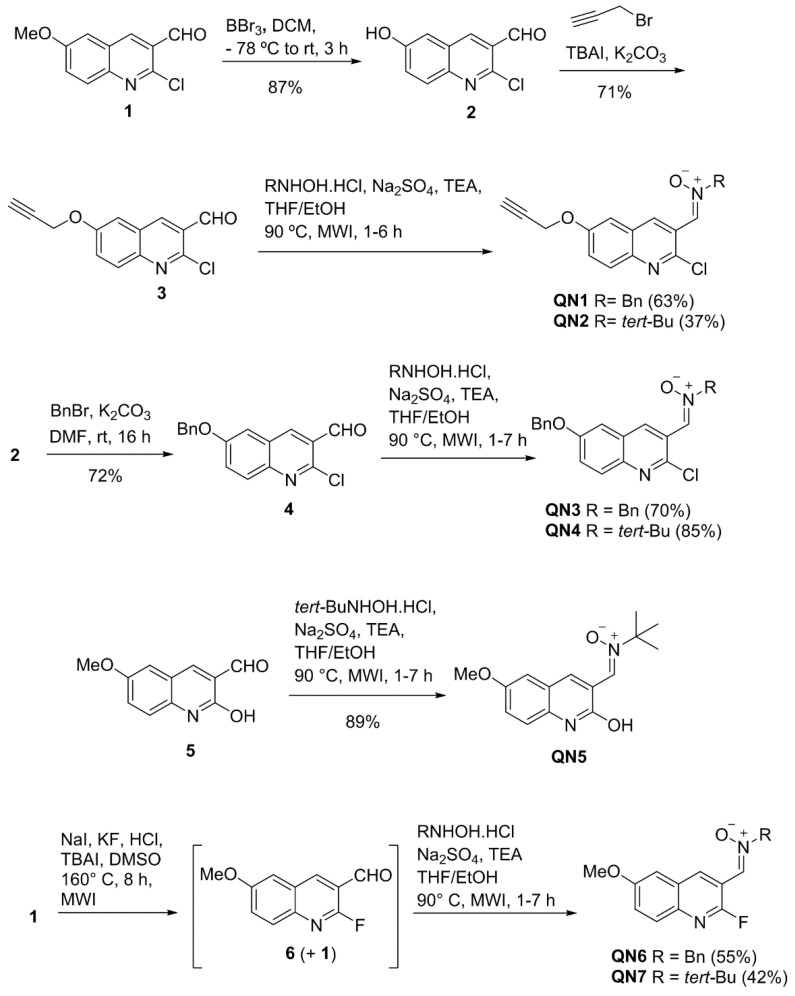

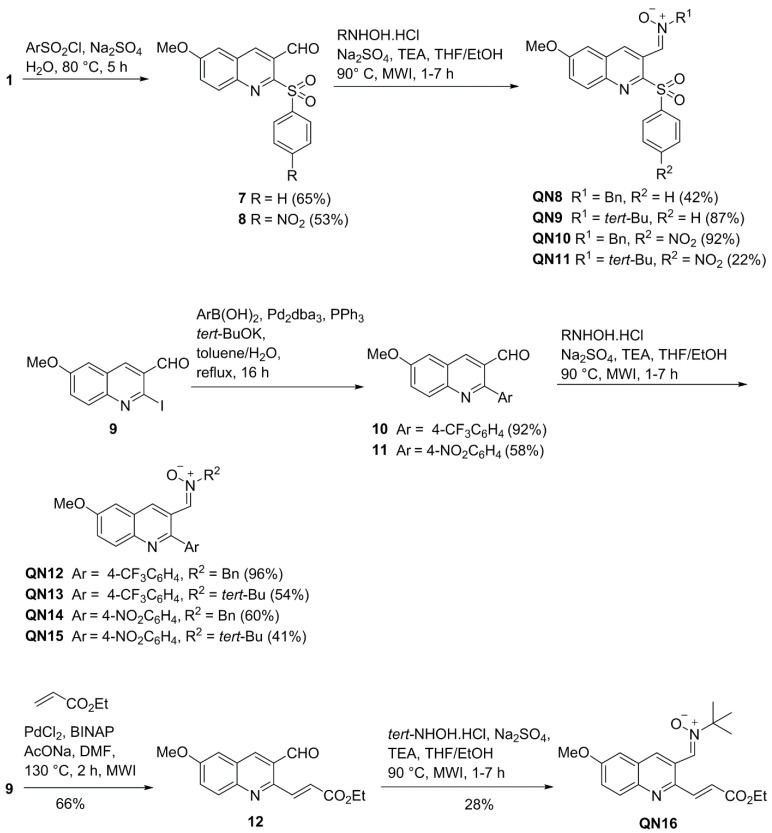
Synthesis of quinolylnitrones QN1–16.

The synthesis of QNs 1–16 has been carried out as shown in Scheme 2. We have applied the general methodology shown in Scheme 1, starting from commercial or readily available carbaldehydes and the appropriate *N*-alkylhydroxylamine hydrochlorides, as described in the Supplementary Material. 

QNs 1–4 were obtained by *O*-propargylation (or *O*-benzylation) of 2-chloro-6-hydroxyquinoline-3-carbaldehyde (2) under mild basic reaction conditions (Scheme 2, see Supplementary Material). 

The synthesis of diversely functionalized QNs 5–16 has been carried out as shown in Scheme 2. Fluorine- and arylsulfonyl-substituted intermediates 6, 7 and 8 (Scheme 2) were prepared by nucleophilic substitution of 2-chloro-6-methoxyquinoline-3-carbaldehyde (1) using KF and arylsulfonyl chlorides, respectively, and following modifications of the reported procedures [20,21]. Thus, reaction of carbaldehydes 6–8 with the appropriate *N*-alkyl hydroxylamine hydrochloride led to the expected QNs 6 and 7 (Scheme 2), and QNs 8–11 (Scheme 2), in moderate to excellent yields. QNs 12–15 substituted with 2-aryl were envisioned through a short reaction sequence including C–C coupling process under Suzuki conditions. Thus, as 2-chloroquinoline 1 was unreactive under the experimental conditions, we tried (Pd_2_(dba)_3_/PPh_3_/*tert*-BuOK) on the more reactive 2-iodo-6-methoxyquinoline-3-carbaldehyde (9), affording the expected aryl-substituted quinolines 10 and 11 with good to excellent yields (Scheme 2). A modification of reported Heck-type reaction on heteroaromatic systems [22], also allowed us the preparation of 2-ethoxypropenyl quinoline carbaldehyde 12 in good yield (Scheme 2). Unfortunately, the reaction of compound 12 with *N*-benzyl hydroxylamine did not afford the corresponding benzyl-substituted nitrone, probably due to intramolecular cycloaddition reactions leading to complex reaction mixtures. Similar side reactions possibly took place in the reaction of intermediate 12 with *N*-*tert*-butyl hydroxylamine, although in this case, we succeeded to isolate 2-ethoxyoxopropenyl quinoline nitrone QN16 in moderate yield after purification by column chromatography (Scheme 2), as the pure and single *E*-isomer, showing the vinyl protons at δ 7.95 and 7.07 as doublets with a vicinal coupling constant of 15.4 Hz. Finally, but unfortunately, other experiments targeted to get quinoline carbaldehydes decorated with carbonitrile or sulfonic acid groups at C2-position were unsuccessful, providing only decomposition products or extremely insoluble reaction mixtures. 

QNs 1–16 have been isolated as single and pure *Z*-isomers, whose structures and stereochemistry are in good agreement with the observed analytical and spectroscopic data (Supplementary Material), as well as with those described in the literature for analogous nitrones [15].

### 2.2. Neuroprotection Evaluation of QNs 1–16 in an Experimental Model of Ischemia on Primary Neuronal Cultures 

**Figure 2:**
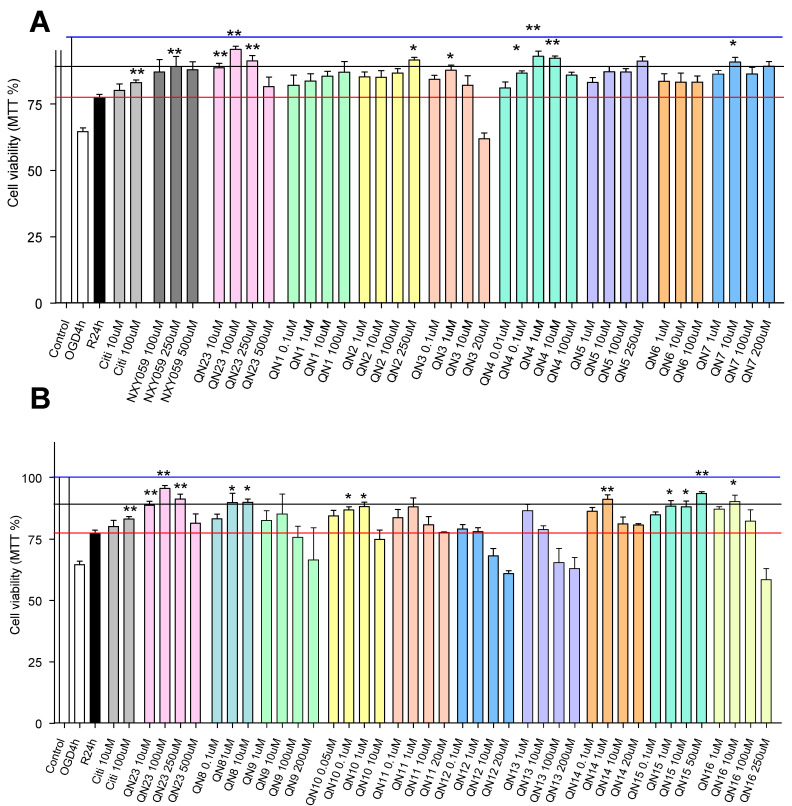
Effect of QNs 1–16 on primary neuronal cultures exposed to OGD and subsequent reoxygenation. Bar chart showing the percentage of cell viability at 24 h of normoxic and normoglycemic recovery after 4 h of OGD, either untreated (R24h) or treated with different concentrations (µM) of citicoline, NXY-059, QN23, or QNs 1–7 (**A**) and QNs 8–16 (**B**) at the onset of the recovery period. The value induced by 4 h of OGD without recovery period (OGD4h) is also indicated. Cell viability of control cells (1.916 ± 0.07 AU) was considered as 100% (blue line). Bars represent the average of four to eight independent experiments; error bars representing the SE. * *p* < 0.05, and ** *p* < 0.01 compared with R24h (red line) by Dunnett’s post-test after ANOVA, when it was significant. Black line shows the increase of cell viability induced by the reference nitrone NXY-059 (250 µM). Statistical significances below R24h value were not shown.

QNs 1–16 were tested in an experimental model of ischemia in order to assess their potential effect on cell viability preservation after an ischemic insult [16,23,24]. To do so, primary neuronal cultures were subjected to OGD conditions for 4 h and treated at the onset of the reoxygenation period with the previously mentioned QNs, or reference compounds such as citicoline—a well-known neuroprotective agent—, NXY-059 or QN23. Cell viability was determined by the MTT assay, for which control group value accounted for 100% (Figure 2). As a consequence of the OGD period, a severe decrease in cell viability was observed, as measured in the unrecovered experimental group (OGD 4h, 64.4 ± 1.4%, *p* < 0.0001 compared with 100% control, one-sample t-test). This impairment in cell metabolism was partially reversed after recovery of normal oxygen and glucose levels for 24 h (R24h; 77.2 ± 1.3%, *p* < 0.0001 vs. control, by Student’s t-test). The addition of citicoline (100 µM) at the onset of the recovery period after OGD prompted a significant increase in cell viability (82.8 ± 1.1%, *p* < 0.01 vs. R24h, by Student’s t-test) compared with the untreated (vehicle) R24h group (Figure 2). When reference compounds NXY-059 or QN23 were added instead of citicoline, higher levels of cell viability were achieved (88.9 ± 3.7% and 95.1 ± 1.4% for 250 µM NXY-059 and 100 µM QN23, respectively) (Figure 2). QNs 1–16 were initially tested on the range 1–250 µM, which has proved adequate as a first approach into the dose-effect assessment of these kind of compounds in our experimental model. For some compounds, however, poor solubility made it impossible to test the highest concentrations. Thus, based on the effect on cell viability obtained for this concentration range and its solubility, we expanded the concentrations tested accordingly. The observed results, shown in Figure 2, were gathered into a structure-activity relationship (SAR) study that is described below.

According to the different structural pattern studied, exploration of *O*-propargyl motif at C6 position on ligands QN1 and QN2 rendered no remarkable overall protective effect in the concentration range tested. Only the *N*-*tert*-Bu-derived QN2 at 250 µM afforded a significant increase in cell viability when compared to R24h, although unfortunately, no higher concentration could be tested due to solubility issues (Figure 2A). 

A superior performance of the *N*-*tert*-Bu derivative in contrast to the *N*-*tert*-Bn substitute was observed for the compounds QN4 and QN3, respectively, both substituted at C6 with the *O*-Bn group. In fact, QN4 showed a good protective profile with a safe concentration range ranging from 0.01 µM to 100 µM, being 1 µM the best performing concentration (Figure 2A). 

The next group of QNs tested included different combinations over the quinoline core substituted with a methoxy group at C6 (Figure 2A). The simplest and the only one of the entire set carrying an electron-donating group at C2 (-OH, QN5) did not give a remarkable protective effect at the concentrations tested. Likewise, molecules QN6 and QN7 bearing a C(2)F substituent did not show significant differences to the value obtained for the vehicle treated group (R24h), except for QN7 at 10 µM. 

The results of the substitution at C2 with other electron-withdrawing groups, different from Cl as in QN23, such as an arylsulfone (QN8–11), a substituted phenyl group (QN12–15) or a α,β-unsaturated carboxylic ester (QN16), are shown in Figure 2B. For the first group, only the *N-tert*-Bn nitrone QN10 showed a significantly higher effect than R24h, both at 0.1 µM and 1 µM doses. Phenyl-substituted ligands QN12 and QN13 with a trifluoromethyl group in *para* position showed toxic effects in the concentration range tested, whilst the *p*-NO_2_-substituted QN14 and QN15 were, in contrast, safe in the whole concentrations range tested. Remarkably, QN15 showed interesting cell viability results when applied in the range 1–50 µM, significantly higher than vehicle-treated group (R24h; 70% at 50 µM). Finally, α,β-unsaturated carboxylic ester QN16 showed interesting results at 10 µM, even though it sharply decreased the beneficial effect with higher concentrations. 

With these results, in order to express cell viability as a more useful concept in our search for protective compounds in the ischemic disease, we defined neuroprotection activity as the effect that achieved a cellular viability higher than the untreated group (R24h), which set our basal neuroprotection value (0%). Cell viability observed in the control group was set as 100% of neuroprotection. Consequently, the neuroprotection values obtained for standards NXY-059, QN23 and the new ligands QNs 1–16 have been gathered together in Table S1 (Supplementary Material), while in Table 2 we have only shown the most active QNs at the diverse tested doses. 

According to these results, QN4 and QN15 are the compounds that exert the highest neuroprotection among the QN1–16 set (Figure 2, Table 2). When compared to our lead compound QN23, neither QN4 nor QN15 reach the cell viability value obtained for QN23 at its best-performing concentration, meaning a slightly decreased activity (67.9% and 70.3% as maximal neuroprotection values for QN4 and QN15, respectively, vs. 79.7% for QN23). On the other hand, both QN4 and QN15 still exert higher cell viability values than reference compound NXY-059, and their effective concentrations are lower than the ones needed for QN23 (1 µM and 50 µM for QN4 and QN15, respectively, vs. 100 µM for QN23), meaning increased pharmacological potency. Finally, solubility and tolerability range must be considered. QN4 showed good solubility and no toxic effects in the range 0.01 µM to 100 µM (100-fold higher than the most effective concentration 1 µM), whereas QN15 was not soluble when assayed at any concentration higher than 50 µM, its most effective concentration. For QN23, its safe concentration range was restricted to 10–500 µM (5-fold higher than its most effective concentration 100 µM). 

**Figure 3:**
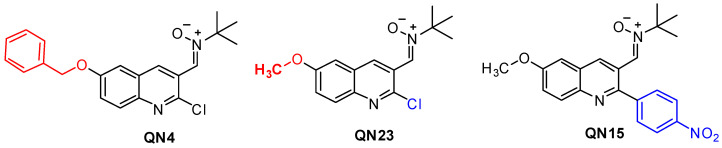
Structure of QN23 and the most potent neuroprotective agents QN4 and QN15 identified in this work.

Noteworthy, when attending to their structural patterns and differences to QN23, QN4 bears an *O*-benzyl group at C6 instead of a methoxy group, whereas QN15 shows a 4-nitroaryl substituent at C2 instead of a chlorine atom, both with a *N-tert*-butyl substituent at the nitrone motif (Figure 3).

### 2.3. Density Functional Theory (DFT) Calculations

**Figure 4:**
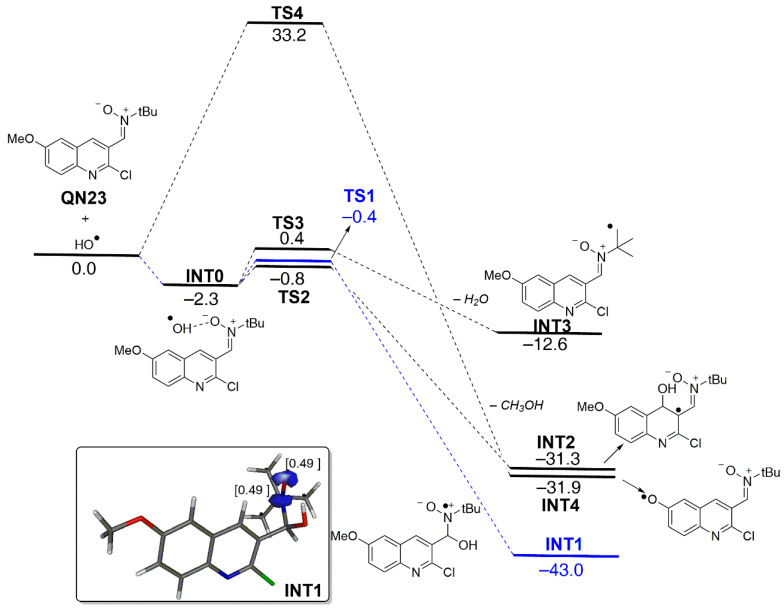
Computed reaction profiles for the reaction of QN23 and HO^•^. Relative free energy values (ΔG, at 298 K) are given in kcal/mol. Inset: computed spin density for **INT1**. All data have been computed at the B3LYP-D3/def2-SVP level.

Density Functional Theory (DFT) calculations were carried out at the dispersion corrected B3LYP-D3/def2-SVP level to gain more insight into the reactivity of **QN23** and related species with HO^•^, as a model radical species. Four different processes, shown in Figure 4, can be envisaged, namely, (i) the addition of the radical to the electrophilic carbon atom of the nitrone, or alternatively, (ii) to the conjugated double-bond involving the pyridine fragment; and the hydrogen atom abstractions involving either (iii) the *tert*-Bu group of the nitrone, or (iv) the Me group of the methoxy substituent (Figure 4).

Our calculations using the *Z*-isomer of QN23, which, in agreement with the experimental observations, is 7.6 kcal/mol more stable than its *E*-counterpart, indicate that the H-abstraction reaction involving the methoxy group is unfeasible in view of the high barrier (ΔG^≠^ = 33.2 kcal/mol) computed for this process. In sharp contrast, the other alternative reactions, which begin from **INT0** (an initial intermediate where the OH^•^ radical is bonded to the oxygen atom of the nitrone moiety via a hydrogen bond) are nearly barrierless processes (ΔG^≠^ < 2 kcal/mol) (Figure 4). Despite that, the computed reaction energies of these processes are rather different. Thus, it becomes clear that both addition reactions are thermodynamically more favored than the H-abstraction involving the *tert*-Bu group of the nitrone. Moreover, the addition to the C=N moiety is much more favored over the process involving the conjugated double-bond, mainly due to two reasons: (a) the significant aromaticity loss in the pyridine ring in **INT2**, and (b) the stabilization of the radical by the oxygen atom in **INT1**, as confirmed by the computed spin density (0.49e in the oxygen and nitrogen atoms, see inset of Figure 4). Therefore, and similar to our previous studies involving other nitrone-based neuroprotective agents [8,13], the mode of action of QN23 (and the related systems described herein) against radical species is dictated by a thermodynamic control, where the addition of the radical to the electrophilic C=N moiety of the nitrone is clearly favored. 

**Figure 5:**
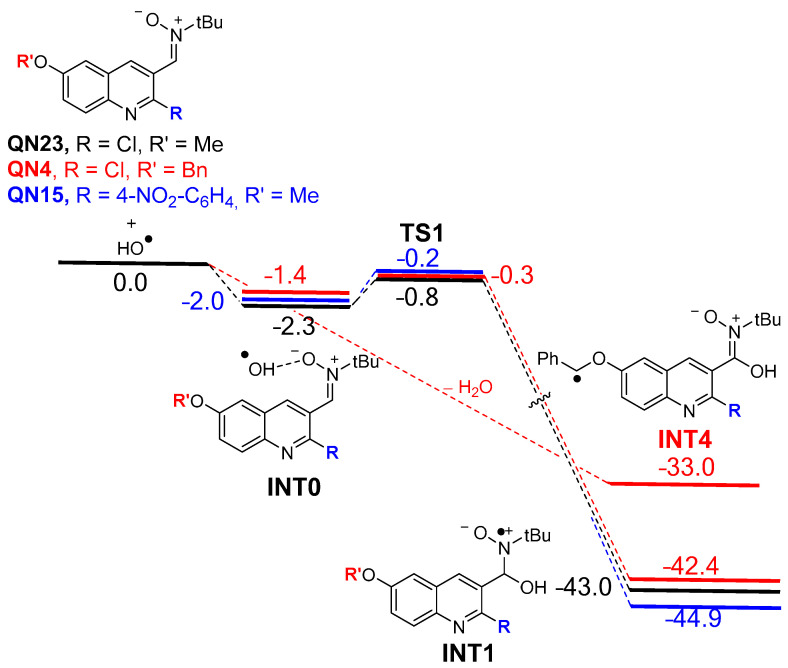
Comparative computed reaction profiles for the reaction of HO^•^ with QN23 (black), QN4 (red) and QN15 (blue) on the electrophilic carbon atom of the nitrone moiety. Relative free energy values (ΔG, at 298 K) are given in kcal/mol. All data have been computed at the B3LYP-D3/def2-SVP level.

Next, we computationally explored the analogous reactions involving compounds QN4 and QN15 to rationalize their presumed outstanding antioxidant and radical scavenging capacities in comparison to the standard QN23 (Figure 5). Not surprisingly, rather similar energy values were computed for all the systems as the reactive nitrone moiety remains practically unaltered. Once again, it can be inferred that, similar to QN23, the high observed neuroprotection activity of ligands QN4 and QN15 may be related to the high exergonicity of the addition reaction of the HO^•^ radical to the electrophilic C=N moiety present on the nitrones. For QN4 in particular, an alternative reaction involving the H-abstraction from the benzylic CH_2_O group could be also envisaged due to the stabilization of the corresponding radical by the adjacent phenyl group (**INT4**, Figure 5). However, our calculations indicate that this alternative process is thermodynamically not favored over the main reaction pathway involving the addition of the hydroxyl radical to the nitrone. 

## 3. Conclusions

In our search for new neuroprotective agents for stroke therapy to improve the neuroprotective efficacy of ligand QN23 [15,19], we have designed, prepared and investigated sixteen new, related, easily available QNs. As a result, we have identified two compounds, QN4 and QN15, showing high neuroprotection capacity, quite similar to that observed for QN23 (Figure 2). At the same time, these results have shown that subtle, but simple functional modifications on the QN scaffold are tolerated, affording new neuroprotective agents, and resulting in a structural pattern that could be of interest for new designs and future developments. In particular, and regarding the structure of compound QN23, QN4 bears a *O*-benzyl group at C6 instead of a methoxy group, whereas QN15 shows a 4-nitroaryl substituent at C2 instead of a chlorine atom, both bearing the *N-tert*-Bu substituent at the nitrone motif (Figure 3). 

Other nitrones, such as azulenyl bis-nitrones, are spin traps having neuroprotective effects in cerebral ischemia [25]. In subsequent studies, a second-generation of the potent antioxidant azulenyl nitrone confirmed their neuroprotective capacity in transient focal cerebral ischemia [26]. In addition, a nitrone with thrombolytic properties, the compound (*Z*)-*N*-*tert*-butyl-1-(3,5,6-trimethylpyrazin-2-yl)methanimine oxide, has been reported for the treatment of ischemic stroke [27]. This is a novel nitrone that shows simultaneous thrombolytic activity and scavenging ROS power for stroke therapy, in addition to nontoxicity and blood−brain barrier permeability. Very interestingly, note that in this case the nitrone motif —the origin of its spin trap capacity— is conjugated with a pyrazine heterocyclic ring, while the nitrones described here are quinolylnitrones and where the nitrone group is conjugated with a related quinoline heterocyclic ring. Furthermore, and not surprisingly, nitrone bears a *tert*-Bu substituent at the nitrogen of the nitrone, the same preferred substituent present in the most potent neuroprotective agents, QN4 and QN15, identified in this work. In this feature, new *para*-substituted nitrones of the potent α-phenyl-*N-tert*-butyl nitrone (PBN) have been reported with good spin-trapping and neuroprotective properties [28].

Remarkably, QN4 and QN15 are non-toxic and safe compounds at all tested doses, despite the toxicological alert described for the nitro group [29]. Finally, and in terms of neuroprotection capacity, the effect induced by QN4 and QN15 is still higher than reference compound NXY-059, the furthest-reaching—but withdrawn in Phase III studies—chemical compound in clinical development for the treatment of CI so far. These data prompt us to keep focusing on the quinolylnitrone core, which proved suitable in the development of not only new neuroprotective compounds, but also able to be easily modified in order to obtain more potent and pharmacologically adequate entities for its further development as potential candidates for ischemic stroke.

## 4. Materials and Methods

### 4.1. Chemistry

#### 4.1.1. General Methods

Melting points were determined on a Köffler apparatus, and are uncorrected. ^1^H NMR and ^13^C NMR spectra were recorded in CDCl_3_ at 300 MHz and at 75 MHz, respectively, using solvent peaks [CDCl_3_: 7.26 (D), 77.2 (C) ppm; D_2_O: 4.60 ppm and DMSO-d_6_: 2.49 (D), 39.52 (C) ppm] as internal reference. Mass spectra were recorded on a GC/MS spectrometer with an API-ES ionization source. Elemental analyses were performed at CNQO (CSIC, Spain). TLCs were performed on silica F254 and detection by UV light at 254 nm, or by charring with either ninhydrin, anisaldehyde or phosphomolybdic-H_2_SO_4_ reagents. Anhydrous solvents were used in all experiments. Column chromatography was performed on silica gel 60 (230 mesh).

#### 4.1.2. General Procedures

##### General Procedure for the Synthesis of Nitrones

A solution of the corresponding carbaldehyde (1 mmol), Na_2_SO_4_ (3 mmol), TEA (2 mmol) and the appropriate hydroxylamine hydrochloride (1.5 mmol) in THF/EtOH (5 mL, 4:1) was heated at 90 °C during 1–6 h under microwave irradiation (MWI). After that time, the solvent was evaporated and the crude mixture was purified on column chromatography using the indicated mixtures of solvents. 

##### General Procedure for the Synthesis of 6-Methoxy-2-(arylsulfonyl)quinoline-3-carbaldehydes 7, 8

A solution of 2-chloro-6-methoxyquinoline-3-carbaldehyde (1) (1 mmol), the corresponding arylsulfonyl chloride (1.2 mmol) and Na_2_SO_3_ (1.3 mmol) in H_2_O (8 mL) was heated at 80 °C in an oil bath for 5 h. After that time, the mixture was diluted with DCM (15 mL), and extracted with NaHCO_3_ (5% water solution), brine, and the organic phase was dried over MgSO_4_. After filtration and evaporation of the solvent, the crude mixture was purified on column chromatography using the indicated mixtures of solvents. Arylsulfonyl quinolines were obtained as mixtures of the expected products and starting material that we were unable to separate, and were directly used in the next step. 

##### General Procedure for the Synthesis of 6-Methoxy-2-arylquinoline-3-carbaldehydes 10, 11

A solution of 2-iodo-6-methoxyquinoline-3-carbaldehyde (9) (1 mmol), the corresponding arylboronic acid (1.2 mmol), Pd_2_dba_3_ (5 mol%), PPh_3_ (5 mol%) and *tert-*BuOK (1 mmol) in a mixture of toluene/H_2_O (5 mL, 4:1) was refluxed during 16 h. Then, the solvent was evaporated and the residue was filtered through a path of Celite. Expected aldehydes were obtained as non-separable mixtures of compounds along with starting material, and therefore directly submitted to the next step without further purification.

#### 4.1.3. (*Z*)-*N*-Benzyl-1-(2-chloro-6-(prop-2-yn-1-yloxy)quinolin-3-yl)methanimine Oxide (QN1) 

Following the General procedure A, the reaction of carbaldehyde 3 (100 mg, 0.408 mmol) with Na_2_SO_4_ (154 mg, 1.224 mmol), TEA (0.11 mL, 0.816 mmol) and *N*-benzylhydroxylamine hydrochloride (97 mg, 0.612 mmol) in THF/EtOH (5 mL, 4:1) for 1 h, gave after work-up and column chromatography (hexane/AcOEt, 4:1) nitrone QN1 as a white solid (45 mg, 63%): mp 122–3 °C; ^1^H NMR (300 MHz, CDCl_3_) δ 10.14 (s, 1H), 8.03 (s, 1H), 7.79 (dd, *J* = 9.2, 2.7 Hz, 1H), 7.51–7.27 (m, 6H, H7, C_6_H_5_), 7.18 (d, *J* = 2.7 Hz, 1H), 5.09 (s, 2H, NCH_2_Ph), 4.71 (d, *J* = 2.4 Hz, 2H, OC*H_2_*C≡CH), 2.48 (t, *J* = 2.4 Hz, 1H, OCH_2_C≡C*H*); ^13^C NMR (75 MHz, CDCl_3_) δ 156.6 (C), 146.5 (C), 143.6 (C), 136.6 (CH), 133.1 (C), 130.0 (CH), 129.8 (CH), 129.79 (CH), 129.74 (2 CH C2′), 129.56 (CH), 129.52 (CH), 128.2 (C), 124.7 (CH), 122.9 (C), 108.4 (CH), 78.1 (C), 76.7 (CH), 72.7 (CH_2_), 56.5 (CH_2_); MS (EI) 350.0 (1) [M^+^]; 315.1 (100) [M^+^-Cl]. HRMS (ESI_ACN). Calcd. for C_20_H_15_ClN_2_O_2_: 350.08221. Found: 350.08263. Anal. Calcd. for C_20_H_15_ClN_2_O_2_.1/3H_2_O: C, 67.32; H, 4.43; N, 7.85. Found: C, 67.34; H, 4.26; N, 7.91. 

#### 4.1.4. (*Z*)-*N-tert*-Butyl-1-(2-chloro-6-(prop-2-yn-1-yloxy)quinolin-3-yl)methanimine Oxide (QN2)

Following the General procedure A, the reaction of carbaldehyde **3** (100 mg, 0.408 mmol) with Na_2_SO_4_ (154 mg, 1.224 mmol), TEA (0.11 mL, 0.816 mmol) and *N-tert*-butylhydroxylamine hydrochloride (77 mg, 0.612 mmol) in THF/EtOH (5 mL, 4:1) for 6 h, after work-up and column chromatography (hexane/AcOEt/DCM, 3:1:1) afforded compound QN2 as a white solid (37 mg, 37%): mp 106–7 °C; ^1^H NMR (300 MHz, CDCl_3_) δ 10.27 (s, 1H), 8.21 (s, 1H), 7.82 (d, *J* = 9.2 Hz, 1H), 7.35 (dd, *J* = 9.2, 2.8 Hz, 1H), 7.24 (d, *J* = 2.8 Hz, 1H), 4.73 (d, *J* = 2.4 Hz, 2H), 2.49 (t, *J* = 2.4 Hz, 1H), 1.61 (s, 9H); ^13^C NMR (75 MHz, CDCl_3_) δ 156.6 (C), 147.1 (C), 143.5 (C), 136.3 (CH), 130.0 (CH), 128.4 (C), 125.8 (CH), 124.5 (CH), 123.4 (C), 108.4 (CH), 78.1 (C), 76.7 (CH), 72.9 (C), 56.5 (CH_2_), 28.7 (3 × CH_3_); MS (EI): 316.1 (2) [M^+^]; 281.1 (32) [M^+^-Cl]; 225.1 (100) [281.1–*tert-*Bu]. HRMS ESI_ACN. Calcd. for C_17_H_17_ClN_2_O_2_: 316.09786. Found: 316.09909. Anal. Calcd. for C_17_H_17_ClN_2_O_2_.½H_2_O: C. 62.67; H, 5.57; N, 8.60, Found: C, 62.44; H, 5.29; N, 8.63. 

#### 4.1.5. (*Z*)-*N*-Benzyl-1-(6-(benzyloxy)-2-chloroquinolin-3-yl)methanimine Oxide (QN3)

Following the General procedure A, the reaction of carbaldehyde 4 (70 mg, 0.236 mmol) with N-benzylhydroxylamine hydrochloride (56 mg, 0.353 mmol), Na_2_SO_4_ (89 mg, 0.708 mmol) and TEA (65 μL, 0.472 mmol) in THF/EtOH (3:0.5 mL), for 1 h, after work-up and column chromatography (hexane/AcOEt, 2:1), gave nitrone QN3, obtained as a white solid (65 mg, 70%): mp 182–3 °C; ^1^H NMR (300 MHz, CDCl_3_) δ 10.09 (s, 1H), 8.02 (s, 1H), 7.79 (d, *J* = 9.2 Hz, 1H), 7.53–7.23 (m, 11H, H7, 2 C_6_H_5_), 7.11 (d, *J* = 2.8 Hz, 1H), 5.08 (s, 4H); ^13^C NMR (75 MHz, CDCl_3_) δ 157.9 (C), 146.2 (C), 143.5 (C), 136.5 (CH), 133.1 (2 C), 130.0 (2 CH), 129.9 (CH), 129.8 (2 CH), 129.7 (CH), 129.5 (2 CH), 129.1 (2 CH), 128.7 (CH), 128.4 (C), 127.9 (CH), 125.1 (CH), 122.8 (C), 108.0 (CH), 72.7 (CH_2_), 70.8 (CH_2_); MS (EI): 402.1 (5) [M^+^]; 367 (100) [M^+^-Cl]. HRMS ESI_ACN. Calcd. for C_24_H_19_ClN_2_O_2_: 402.11351. Found: 402.11419. Anal. Calcd. for C_24_H_19_ClN_2_O_2_: C, 70.50; H, 4,85; N, 6.85. Found: C, 70.39; H, 4.79; N, 6.81. 

#### 4.1.6. (*Z*)-*N-tert*-Butyl-1-(6-(benzyloxy)-2-chloroquinolin-3-yl)methanimine Oxide (QN4)

Following the General procedure A, the reaction of carbaldehyde 4 (70 mg, 0.236 mmol) with *N-tert*-butylhydroxylamine hydrochloride (44 mg, 0.353 mmol), Na_2_SO_4_ (89 mg, 0.708 mmol) and TEA (65 μL, 0.472 mmol) in THF/EtOH (3:0.5 mL), for 6 h, gave after work-up and column chromatography (hexane/AcOEt, 7:3) nitrone QN4, obtained as a white solid (73 mg, 85%): mp 205–6 °C; ^1^H NMR (300 MHz, CDCl_3_) δ 10.19 (s, 1H), 8.19 (s, 1H), 7.80 (d, *J* = 9.1 Hz, 1H), 7.45–7.24 (m, 6H), 7.13 (d, *J* = 2.8 Hz, 1H), 5.10 (s, 2H), 1.60 (s, 9H); ^13^C NMR (75 MHz, CDCl_3_) δ 157.9 (C), 146.8 (C), 143.3 (C), 136.5 (CH), 136.2 (C), 129.9 (CH), 129.1 (2 CH), 128.67 (CH), 128.63 (C), 127.8 (2 CH), 125.8 (CH), 124.8 (CH), 123.3 (C), 108.1 (CH), 72.8 (C), 70.7 (CH_2_), 28.7 (3 × CH_3_); MS (EI): 367.1 (2) [M^+^]; 333.1 (37) [M^+^-Cl]. HRMS ESI_ACN. Calcd. for C_21_H_21_ClN_2_O_2_: 368.12916. Found: 368.12987. Anal. Calcd. for C_21_H_21_ClN_2_O_2_: C, 68.35; H, 5,74; N, 7.59. Found: C, 68.22; H, 5.74; N, 7.58. 

#### 4.1.7. (*Z*)-*N-tert*-Butyl-1-(2-hydroxy-6-methoxyquinolin-3-yl)methanimine Oxide (QN5)

Following the General procedure A, the reaction of commercial compound 5 (50 mg, 0.153 mmol) with *N-tert*-butylhydroxylamine hydrochloride (30 mg, 0.229 mmol), Na_2_SO_4_ (60 mg, 0.459 mmol) and TEA (42, 0.306 mmol) in THF (2 mL), for 6 h, after work-up and column chromatography (DCM/AcOEt, 1:2), gave nitrone QN5 as a yellow solid (40 mg, 89%): mp 172–3 °C; ^1^H NMR (300 MHz, CDCl_3_) δ 11.77 (br s, 1H), 10.06 (s, 1H), 8.26 (s, 1H), 7.28–7.15 (m, 1H), 7.09 (dd, *J* = 8.9, 2.6 Hz, 1H), 6.99 (d, *J* = 2.6 Hz, 1H), 3.76 (s, 3H, OCH_3_), 1.59 (s, 9H); ^13^C NMR (75 MHz, CDCl_3_) δ 162.7 (C), 155.8 (C), 137.8 (CH), 133.1 (C), 125.3 (CH), 122.7 (C), 121.6 (CH), 121.4 (C), 117.1 (CH), 110.3 (CH), 72.1 (C), 56.0 (CH_3_), 28.7 (3 x CH_3_); MS (EI): 274.1 (62) [M^+^]. HRMS ESI_ACN. Calcd. for C_15_H_18_N_2_O_3_: 274.13174. Found: 274.13130. Anal. Calcd for C_15_H_18_N_2_O_3_: C, 65.68; H, 6,61; N, 10.21. Found: C, 65.39; H, 6.58; N, 9.97.

#### 4.1.8. (*Z*)-*N*-Benzyl-1-(6-(methoxy)-2-fluoroquinolin-3-yl)methanimine oxide (QN6)

A solution of 2-chloro-6-methoxyquinoline-3-carbaldehyde (1) (70 mg, 0.317 mmol), NaI (190 mg, 1.267 mmol), KF (147 mg, 2.536 mmol) and catalytic amount of HCl and TBAI in DMSO (1 mL), was heated at 160 °C for 8 h under MWI. Then, the mixture was diluted with AcOEt and washed with H_2_O (3 × 10 mL). The organic phase was washed with brine and dried over MgSO_4_. After evaporation of the solvent, the crude mixture was purified by column chromatography (hexane/AcOEt, 7:3) to yield a mixture of major 2-fluoro-6-methoxyquinoline-3-carbaldehyde (6) {^1^H NMR (300 MHz, CDCl_3_) δ 10.32 (s, 1H, CHO), 8.35 (s, 1H, H4), 7.88 (dd, *J* = 9.2, 1.0 Hz, 1H, H8), 7.43 (dd, *J* = 9.2, 2.8 Hz, 1H), 7.10 (dd, *J* = 2.8, 1.0 Hz, 1H), 3.93 (s, 3H, OCH_3_); MS (EI): 206.1 (100) [M+1]} and minor starting material 1, that we could not separate (55 mg of a mixture of compounds 9/1 in 1:0.3 ^1^H NMR ratio). Compound 6, obtained in 65% based on the ^1^H NMR spectrum, was used in the next steps without further purification. Following the General procedure A, the reaction of the mixture 6 + 1 (60 mg) with *N*-benzylhydroxylamine hydrochloride (70 mg, 0.439 mmol), Na_2_SO_4_ (111 mg, 0.879 mmol) and TEA (81 μL, 0.586 mmol) in THF/EtOH (3:0.5 mL), for 1 h, after work-up and column chromatography (hexane/AcOEt/Et_2_O, 4:1:1) gave nitrone **QN6** as a white solid (50 mg, 55%): mp 197–9 °C; ^1^H NMR (300 MHz, CDCl_3_) δ 10.08 (d, *J* = 9.8 Hz, 1H), 7.77 (s, 1H), 7.69 (d, *J* = 9.1 Hz, 1H), 7.49–7.22 (m, 6H), 7.08 (d, *J* = 2.8 Hz, 1H), 5.07 (s, 2H), 3.82 (s, 3H); ^13^C NMR (75 MHz, CDCl_3_) δ 158.3 (C, d, *J* = 2.2 Hz), 156.5 (d, *J* = 242 Hz, C), 140.1 (C, d, *J* = 68.4 Hz), 138.5 (d, *J* = 3.4 Hz, CH), 133.1 (C), 129.7 (2 CH), 129.5 (2 CH), 129.2 (CH), 128.4 (C), 126.6 (CH), 124.4 (CH), 114.1 (C, d, *J* = 28.5 Hz), 107.2 (CH), 72.4 (CH_2_), 56.0 (CH_3_); MS (EI): 310.1 (100) [M^+^]. HRMS ESI_ACN Calcd. for C_18_H_15_FN_2_O_2_: 310.11176. Found: 310.11076. Anal. Calcd for C_18_H_15_FN_2_O_2_: C, 69.67; H, 4,87; N, 9.03. Found: C, 69.52; H, 5.01; N, 9.04. 

#### 4.1.9. (*Z*)-*N-tert*-Butyl-1-(6-(methoxy)-2-fluoroquinolin-3-yl)methanimine Oxide (QN7)

Following the General procedure A, the reaction of mixture 6 + 1 (100 mg) with N-tert-butylhydroxylamine hydrochloride (88 mg, 0.731 mmol), Na_2_SO_4_ (184 mg, 1.461 mmol) and TEA (135 μL, 0.974 mmol) in THF (4 mL), for 6 h, after work-up and column chromatography (hexane/AcOEt/MeOH, 10:5:1) gave nitrone QN7, as a white solid (57 mg, 42%): mp 156–7 °C; ^1^H NMR (300 MHz, CDCl_3_) δ 10.26 (d, *J* = 9.8 Hz, 1H), 7.96 (s, 1H), 7.77 (d, *J* = 9.2 Hz, 1H), 7.37 (dd, *J* = 9.2, 2.8 Hz, 1H), 7.18 (s, 1H), 3.89 (s, 3H), 1.65 (s, 9H); ^13^C NMR (75 MHz, CDCl_3_) δ 158.2 (C, d, *J* = 2.0 Hz), 156.8 (d, *J* = 239 Hz, C), 140.7 (C, d, *J* = 17.4 Hz), 138.2 (d, *J* = 3.5 Hz, CH), 129.2 (CH), 128.5 (C), 124.0 (C), 122.4 (CH), 114.5 (C, d, *J* = 27.7 Hz), 107.2 (CH), 72.6 (C), 55.9 (CH_3_), 28.7 (3 × CH_3_); MS (EI): 276.1 (57) [M^+^]. HRMS ESI_ACN. Calcd. for C_15_H_17_FN_2_O_2_: 276.12741. Found: 276.12834. Anal. Calcd. C_15_H_17_FN_2_O_2_: C, 63.15; H, 6.36; N, 9.82. Found: C, 63.35; H, 6.15; N, 9.74. 

#### 4.1.10. (*Z*)-*N*-Benzyl-1-(6-methoxy-2-(phenylsulfonyl)quinolin-3-yl)methanimine Oxide (QN8)

Following the General procedure B, the reaction of 2-chloro-6-methoxyquinoline-3-carbaldehyde (1) (42 mg, 0.189 mmol) with phenylsulfonyl chloride (72 μL, 0.567 mmol) and Na_2_SO_3_ (71 mg, 0.567 mmol) in H_2_O (0.7 mL), after purification on column chromatography (hexane/AcOEt, 2:1) gave a mixture of unreacted compound 1 and 6-methoxy-2-(phenylsulfonyl)quinoline-3-carbaldehyde (7) (40 mg, 65%, yield estimated on the ^1^H NMR basis spectrum), as a thick gum {^1^H NMR (300 MHz, CDCl_3_) (for major compound 7) δ 11.09 (s, 1H), 8.69 (s, 1H), 8.12–7.96 (m, 2H), 7.85 (d, *J* = 9.2 Hz, 1H), 7.64–7.48 (m, 3H), 7.42 (dd, *J* = 9.2, 2.7 Hz, 1H), 7.14 (d, *J* = 2.7 Hz, 1H), 3.89 (s, 3H); MS (EI): 327.1 (5) [M^+^]}. Following the General procedure A, the reaction of mixture 1 + 7 (200 mg, 0.611 mmol) with *N*-benzylhydroxylamine hydrochloride (146 mg, 0.917 mmol), Na_2_SO_4_ (260 mg, 1.835 mmol) and TEA (170 μL, 1.223 mmol) in THF (4 mL), for 1 h and work-up, after purification on column chromatography (hexane/AcOEt, 2:1) gave the expected nitrone QN8 as a light yellow solid (97 mg, 42%): mp 164–6 °C; ^1^H NMR (300 MHz, CDCl_3_) δ 10.29 (s, 1H), 8.89 (s, 1H), 7.87 (dd, *J* = 8.4, 1.4 Hz, 1H), 7.69–7.25 (m, 11H), 7.04 (d, *J* = 2.7 Hz, 1H), 5.12 (s, 2H), 3.82 (s, 3H); ^13^C NMR (75 MHz, CDCl_3_) δ 160.5 (C), 152.5 (C), 141.6 (C), 139.1 (C), 136.3 (CH), 134.1 (C), 133.3 (CH), 131.6 (CH), 130.3 (C), 130.0 (2 CH), 129.7 (2 CH), 129.5 (2 CH), 129.4 (2 CH), 129.2 (CH), 128.4 (CH) 125.3 (CH), 121.4 (C), 106.1 (CH), 73.0 (CH_2_), 56.1 (CH_3_); MS (EI): 432.1 (3) [M^+^], 291.1 (100) [M^+^-SO_2_Ph]. HRMS ESI_ACN. Calcd. for C_24_H_20_N_2_O_4_S: 432.11438. Found: 432.11454. Anal. Calcd. for C_24_H_20_N_2_O_4_S: C, 66.65; H, 4.66; N, 6.48; S, 7.41. Found: C, 66.88; H, 4.88; N, 6.74; S, 7.26.

#### 4.1.11. (*Z*)-*N-tert-*Butyl-1-(6-methoxy-2-(phenylsulfonyl)quinolin-3-yl)methanimine Oxide (QN9)

Following the General procedure A, the reaction of mixture 1 + 7 (70 mg, 0.214 mmol) with *N-tert*-butylhydroxylamine hydrochloride (90 mg, 0.321 mmol), Na_2_SO_4_ (180 mg, 0.642 mmol) and TEA (126 μL, 0.428 mmol) in THF (5 mL), for 6 h and work-up, after column chromatography (hexane/AcOEt, 2:1) gave nitrone QN9 obtained as a light yellow solid (80 mg, 87%): mp 137–8 °C; ^1^H NMR (300 MHz, CDCl_3_) δ 10.39 (s, 1H), 9.04 (s, 1H), 8.00–7.90 (m, 2H), 7.70–7.41 (m, 4H), 7.27 (dd, *J* = 9.2, 2.7 Hz, 1H), 7.07 (d, *J* = 2.7 Hz, 1H), 3.83 (s, 3H), 1.62 (s, 9H); ^13^C NMR (75 MHz, CDCl_3_) δ 160.4 (C), 152.9 (C), 141.4 (C), 139.2 (C), 136.0 (CH), 134.1 (CH), 131.5 (CH), 130.5 (C), 129.6 (2 CH), 129.1 (2 CH), 124.9 (CH), 124.3 (CH), 122.0 (C), 106.1 (CH), 73.1 (C), 28.7 (3 x CH_3_); MS (EI): 398.1 (3) [M^+^], 257.1 (27) [M^+^-SO_2_Ph]. HRMS ESI_ACN. Calcd. for C_21_H_22_N_2_O_4_S: 398.13003. Found: 398.13047. Anal. Calcd. for C_21_H_22_N_2_O_4_S: C, 62.36; H, 5.65; N, 6.93; S, 7.93 Found: C, 62.49; H, 5.54; N, 6.75; S, 7.84.

#### 4.1.12. (*Z*)-*N-*Benzyl-1-(2-((4-nitrophenyl)sulfonyl)-6-methoxyquinolin-3-yl) methanimine Oxide (QN10)

Following the General procedure B, the reaction of 2-chloro-6-methoxyquinoline-3-carbaldehyde (1) (200 mg, 0.904 mmol) with 4-nitrobenzenesulfonyl chloride (239 mg, 1.085 mmol) and Na_2_SO_3_ (148 mg, 1.175 mmol) in H_2_O (3 mL), after purification on column chromatography (hexane/AcOEt, 4:1), afforded 6-methoxy-2-((4-nitrophenyl)sulfonyl)quinoline-3-carbaldehyde (8) (178 mg, 53%, yield estimated on the ^1^H NMR basis spectrum), a thick yellow gum, as a mixture of non-separable compounds 8 and 1 [^1^H NMR (300 MHz, CDCl_3_) (for compound 8) δ 10.55 (d, *J* = 0.7 Hz), 8.64 (d, *J* = 0.7 Hz, 1H), 8.42 (d, *J* = 8.7 Hz, 2H), 8.30 (d, *J* = 8.7 Hz, 2H), 7.96 (dd, *J* = 9.3, 0.7 Hz, 1H), 7.52 (dd, *J* = 9.3, 2.8Hz, 1H), 7.19 (d, *J* = 2.8 Hz, 1H)]. Following the General procedure A, the reaction of mixture 1 + 8 (90 mg, 0.410 mmol) with *N*-benzylhydroxylamine hydrochloride (98 mg, 0.615 mmol), Na_2_SO_4_ (116 mg, 0.821 mmol) and TEA (170 μL, 1.231 mmol) in THF (3 mL), for 1 h, after work-up and purification on column chromatography (DCM/AcOEt, 5:1), gave nitrone QN10 as a yellow solid (80 mg, 92%): mp 175–7 °C; ^1^H NMR (300 MHz, CDCl_3_) δ 10.31 (s, 1H), 8.79 (s, 1H), 8.30 (d, *J* = 9.0 Hz, 2H), 8.10 (d, *J* = 9.0 Hz, 2H), 7.62–7.22 (m, 7H), 7.06 (d, *J* = 2.0 Hz, 1H), 5.14 (s, 2H), 3.83 (s, 3H); ^13^C NMR (75 MHz, CDCl_3_) δ 160.7 (C), 151.7 (C), 151.2 (C), 144.7 (C), 141.2 (C), 136.4 (CH), 133.1 (C), 131.4 (2 CH), 131.3 (C), 130.4 (2 CH), 129.9 (CH), 129.8 (CH), 129.5 (2 CH), 127.5 (CH), 125.6 (CH), 124.1 (2 CH), 121.3 (C), 106.1 (CH), 73.1 (CH_2_), 56.2 (CH_3_); MS (EI): 477.1 (1) [M^+^], 291.1 (100) [M^+^-SO_2_Ar]. HRMS ESI_ACN. Calcd. for C_24_H_19_N_3_O_6_S: 477.09946. Found: 477.09948. Anal. Calcd. for C_24_H_19_N_3_O_6_S: C, 59.25; H, 4.14; N, 8.64; S, 6.59. Found: C, 59.19; H, 4.12; N, 8.74; S, 6.54.

#### 4.1.13. (*Z*)-*N-tert*-Butyl-1-(2-((4-nitrophenyl)sulfonyl)-6-methoxyquinolin-3-yl) methanimine Oxide (QN11)

Following the General procedure A, the reaction of mixture 1 + 8 (116 mg, 0.525 mmol), with *N-tert-*butylhydroxylamine hydrochloride (98 mg, 0.615 mmol), Na_2_SO_4_ (98 mg, 0.791 mmol) and TEA (220 μL, 1.550 mmol) in THF (5 mL), for 6 h, after work-up and purification on column chromatography (DCM/AcOEt, 10:1), gave nitrone QN11 as a yellow solid (51 mg, 22%): mp 144–5 °C; ^1^H NMR (300 MHz, CDCl_3_) δ 10.41 (s, 1H), 8.99 (s, 1H), 8.36 (d, *J* = 8.9 Hz, 2H), 8.18 (d, *J* = 8.9 Hz, 2H), 7.52 (d, *J* = 9.2 Hz, 1H), 7.26 (dd, *J* = 9.2, 2.8 Hz, 1H), 7.09 (d, *J* = 2.8 Hz, 1H), 3.84 (s, 3H), 1.64 (s, 9H); ^13^C NMR (75 MHz, CDCl_3_) δ 160.6 (C), 152.2 (C), 151.2 (C), 144.8 (C), 141.1 (C), 136.1 (CH), 131.4 (2 CH), 131.2 (CH), 130.6 (C), 125.2 (CH), 124.1 (2 CH), 123.5 (CH), 121.9 (C), 106.2 (CH), 73.3 (C), 56.1 (CH_3_), 28.7 (3 × CH_3_); MS (EI): 443.1 (3) [M^+^], 257.1 (32) [M^+^-SO_2_Ar]. HRMS ESI_ACN. Calcd. for C_21_H_21_N_3_O_6_S: 443.11511. Found: 443.11544. Anal. Calcd. for C_21_H_21_N_3_O_6_S: C, 56.88; H, 4.77; N, 9.48; S, 7.23 Found: C, 56.68; H, 4.88; N, 9.41; S, 7.06.

#### 4.1.14. 6-Methoxy-2-(4-trifluoromethylphenyl)quinoline-3-carbaldehyde (10)

Following the General procedure C, the reaction of commercial 2-iodo-6-methoxyquinoline-3-carbaldehyde (9) (50 mg, 0.159 mmol), *p*-trifluoromethylphenylboronic acid (36 mg, 0.191 mmol), *tert-*BuOK (18 mg, 0.159 mmol), Pd_2_dba_3_ (7 mg, 0.007 mmol) and PPh_3_ (2 mg, 0.007 mmol) in toluene/H_2_O (4 + 1 mL), after purification on column chromatography (hexane/AcOEt, 5:1), gave compound 10 (48 mg, 92%) which was obtained as a yellow solid: ^1^H NMR (300 MHz, CDCl_3_) δ 10.09 (s, 1H), 8.68 (s, 1H), 8.03 (d, *J* = 9.2 Hz, 1H), 7.74 (s, 4H), 7.48 (dd, *J* = 9.2, 2.8 Hz, 1H), 7.19 (s, 1H), 3.92 (s, 3H). HRMS ESI_ACN. Calcd. for C_18_H_12_NF_3_O_2_: 331.08201. Found: 331.08222.

#### 4.1.15. (*Z*)-*N*-Benzyl-1-(6-methoxy-2-(4-(trifluoromethyl)phenyl)quinolin-3-yl) methanimine Oxide (QN12)

Following the General procedure A, the reaction of compound 10 (100 mg, 0.302 mmol) with *N*-benzylhydroxylamine hydrochloride (71 mg, 0.453 mmol), Na_2_SO_4_ (86 mg, 0.604 mmol) and TEA (120 μL, 0.906 mmol) in THF (3 mL), for 1 h, after purification on column chromatography (DCM/AcOEt, 10:1), gave nitrone QN12 as a yellow solid (120 mg, 96%): mp 234–5 °C; ^1^H NMR (300 MHz, CDCl_3_) δ 10.15 (s, 1H), 7.88 (d, *J* = 9.2 Hz, 1H), 7.56 (d, *J* = 8.0 Hz, 2H), 7.44 (d, *J* = 8.0 Hz, 2H), 7.37–7.22 (m, 6H), 7.19 (d, *J* = 4.8 Hz, 1H), 7.13 (d, *J* = 2.7 Hz, 1H), 4.95 (s, 2H), 3.87 (s, 3H); ^13^C NMR (75 MHz, CDCl_3_) δ 158.8 (C), 155.0 (C), 143.9 (C), 143.9 (C), 143.2 (C), 134.7 (CH), 132.8 (C), 131.3 (CH), 131.0 (CH), 130.2 (2 CH), 130.0 (2 CH), 129.8 (2 CH), 129.5 (2 CH), 128.5 (C), 125.93, 125.88, 125.83, 125.78 (C, q, *J* = 15.0 Hz), 124.6 (CH), 122.4 (C), 106.4 (CH), 72.2 (CH_2_), 56.0 (CH_3_); MS (EI): 436.1 (29) [M^+^]. HRMS ESI_ACN. Calcd. for C_25_H_19_F_3_N_2_O_2_: 436.13986. Found: 436.13835. Anal. Calcd. for C_25_H_19_F_3_N_2_O_2_: C, 67.87; H, 4.48; N, 6.33. Found: C, 67.90; H, 4.65; N, 6.52.

#### 4.1.16. (*Z*)-*N-tert*-Butyl-1-(6-methoxy-2-(4-(trifluoromethyl)phenyl)quinolin-3-yl) methanimine Oxide (QN13)

Following the General procedure A, the reaction of compound 10 (120 mg, 0.363 mmol) with *N-tert*-butylhydroxylamine hydrochloride (68 mg, 0.544 mmol), Na_2_SO_4_ (103 mg, 0.726 mmol) and TEA (150 μL, 1.089 mmol) in THF (3 mL), for 6 h, after purification on column chromatography (DCM/AcOEt, 7:1), gave nitrone QN13 as a yellow solid (78 mg, 54%): mp 192–4 °C; ^1^H NMR (300 MHz, CDCl_3_) δ 10.25 (s, 1H), 7.91 (ddd, *J* = 9.2, 2.8, 1.2 Hz, 1H), 7.72 (d, *J* = 9.1 Hz, 2H), 7.65 (d, *J* = 9.1 Hz, 2H), 7.59 (s,1H), 7.34 (dd, *J* = 9.2, 2.8 Hz, 1H), 7.14 (dd, *J* = 2.8, 1.2 Hz, 1H), 3.87 (s, 3H), 1.45 (s, 9H); ^13^C NMR (75 MHz, CDCl_3_) δ 158.8 (C), 155.3 (C), 143.8 (3 C), 134.7 (CH), 131.0 (CH), 130.9 (2 CH), 130.4 (2 CH), 128.6 (C), 127.0 (CH), 126.7 (q, *J* = 270.5 Hz, C), 125.9, 125.9, 125.8, 125.8 (C, q, *J* = 15.0 Hz), 124.3 (CH), 122.9 (C), 106.4 (CH), 72.3 (C), 56.0 (CH_3_), 28.6 (3 × CH_3_). HRMS ESI_ACN. Calcd. for C_22_H_21_F_3_N_2_O_2_: 402.15551. Found: 402.15507. Anal. Calcd. for C_22_H_21_F_3_N_2_O_2_: C, 64.94; H, 5.33; N, 6.90. Found: C, 64.77; H, 5.32; N, 7.17.

#### 4.1.17. 6-Methoxy-2-(4-nitrophenyl)quinoline-3-carbaldehyde (11)

Following the General procedure C, the reaction of compound 9 (100 mg, 0.452 mmol), *p*-nitrophenylboronic acid (90 mg, 0.542 mmol), *tert-*BuOK (50 mg, 0.452 mmol), Pd_2_dba_3_ (21 mg, 0.022 mmol) and PPh_3_ (18 mg, 0.068 mmol) in toluene/H_2_O (4 + 1 mL), after purification on column chromatography (hexane/AcOEt, 8:2), gave a mixture of 9 + 11 [^1^H NMR (300 MHz, CDCl_3_) (major compound 11) [δ 10.17 (s, 1H), 8.77 (s, 1H), 8.42 (dd, *J* = 9.0, 2.3 Hz, 2H), 8.11 (d, *J* = 9.2 Hz, 1H), 7.86 (dd, *J* = 9.0, 2.3 Hz, 2H), 7.57 (dd, *J* = 9.2, 1.9 Hz, 1H), 7.26 (m, 1H), 4.00 (s, 3H)] (81 mg, 58%, yield estimated by ^1^H NMR in the reaction mixture) and was obtained as a non-separable mixture with starting material. The mixture of compounds was submitted to the next step without further purification. 

#### 4.1.18. (*Z*)-*N*-Benzyl-1-(6-methoxy-2-(4-nitrophenyl)quinolin-3-yl)methanimine Oxide (QN14)

Following the General procedure A, the reaction of mixture 9 + 11 (100 mg, 0.325 mmol) with *N*-benzylhydroxylamine hydrochloride (77 mg, 0.487 mmol), Na_2_SO_4_ (138 mg, 0.975 mmol) and TEA (90 μL, 0.650 mmol) in THF (3.2 mL), for 1 h, after purification on column chromatography (DCM/AcOEt, 2:1), gave nitrone QN14 as a yellow solid (80 mg, 60%): mp 251–3 °C; ^1^H NMR (300 MHz, CDCl_3_) δ 10.13 (s, 1H), 8.15 (d, *J* = 8.7 Hz, 2H), 7.88 (d, *J* = 9.2 Hz, 1H), 7.51 (d, *J* = 8.7 Hz, 2H), 7.39–7.24 (m, 6H), 7.18–7.16 (m, 1H), 7.14–7.12 (m, 1H), 4.96 (s, 2H), 3.88 (s, 3H); ^13^C NMR (75 MHz, CDCl_3_) δ 159.1 (C), 153.9 (C), 148.0 (C), 146.0 (C), 143.9 (C), 134.8 (CH), 132.8 (C), 131.0 (2 CH), 130.9 (2 CH), 130.0 (CH), 129.8 (CH), 129.7 (2 CH), 129.6 (CH), 128.7 (C), 124.8 (CH), 124.0 (2 CH), 122.3 (C), 106.3 (CH), 72.3 (CH_2_), 56.1 (CH_3_); MS (EI): 413.1 (23) [M^+^], 291.1 (100) [M^+^-C_6_H_4_NO_2_]. HRMS ESI_ACN. Calcd. for C_24_H_19_N_3_O_4_: 413.13756. Found: 413.12755. Anal. Calcd. for C_24_H_19_N_3_O_4_: C, 64.55; H, 4.42; N, 9.22. Found: C, 64.50; H, 4.33; N, 8.99.

#### 4.1.19. (*Z*)-*N-tert*-Butyl-1-(6-methoxy-2-(4-nitrophenyl)quinolin-3-yl)methanimine Oxide (QN15)

Following the General procedure A, the reaction of mixture 9 + 11 (100 mg, 0.325 mmol) with *N-tert*-butylhydroxylamine hydrochloride (61 mg, 0.487 mmol), Na_2_SO_4_ (138 mg, 0.975 mmol) and TEA (90 μL, 0.650 mmol) in THF (3.2 mL), for 6 h; after purification on column chromatography (DCM/AcOEt, 4:1), gave nitrone QN15, as a yellow solid (45 mg, 41%): mp 189–191 °C; ^1^H NMR (300 MHz, CDCl_3_) δ 10.23 (s, 1H), 8.32 (d, *J* = 8.8 Hz, 2H), 7.91 (d, *J* = 9.2 Hz, 1H), 7.74 (d, *J* = 8.8 Hz, 2H), 7.56 (s, 1H), 7.36 (dd, *J* = 9.2, 2.8 Hz, 1H), 7.14 (d, *J* = 2.8 Hz, 1H), 3.87 (s, 3H), 1.46 (s, 9H); ^13^C NMR (75 MHz, CDCl_3_) δ 159.0 (C), 154.2 (C), 148.3 (C), 146.6 (C), 143.8 (C), 134.8 (CH), 131.1 (2 CH), 131.0 (CH), 128.8 (C), 126.6 (CH), 124.6 (CH), 124.1 (2 CH), 122.9 (C), 106.3 (CH), 72.4 (C), 56.0 (CH_3_), 28.6 (3 x CH_3_); MS (EI): 379.1 (38) [M^+^], 322.1 (80) [M^+^-*tert-*Bu]. HRMS ESI_ACN. Calcd. for C_21_H_21_N_3_O_4_: 379.15321. Found: 379.11535. Anal. Calcd. for C_21_H_21_N_3_O_4_: C, 66.48; H, 5.58; N, 11.08. Found C, 66.34; H, 5.46; N, 10.37.

#### 4.1.20. (*Z*)-*N-tert*-Butyl-1-(2-((E)-3-ethoxy-3-oxoprop-1-en-1-yl)-6-methoxyquinolin-3-yl)methanimine Oxide (QN16)

Following the General procedure A, the reaction of compound 12 (80 mg, 0.280 mmol) with *N-tert*-butylhydroxylamine hydrochloride (52 mg, 0.421 mmol), Na_2_SO_4_ (119 mg, 0.843 mmol) and TEA (78 μL, 0.562 mmol) in THF (2 mL) for 6 h, after work-up and purification on column chromatography (DCM/AcOEt, 7:3), gave nitrone QN16 as a yellow solid (26 mg, 26%); mp 152–4 °C; ^1^H NMR (300 MHz, CDCl_3_) δ 10.05 (s, 1H), 7.98 (s, 1H), 7.95 (d, *J* = 15.4 Hz, 1H), 7.86 (d, *J* = 9.2 Hz, 1H), 7.31 (dd, *J* = 9.2, 2.8 Hz, 1H), 7.07 (d, *J* = 15.4 Hz, 1H), 7.06 (d, *J* = 7.4 Hz, 1H), 4.24 (q, *J* = 7.1 Hz, 2H), 3.84 (s, 3H), 1.62 (s, 9H), 1.29 (t, *J* = 7.1 Hz, 3H); ^13^C NMR (75 MHz, CDCl_3_) δ 167.1 (C), 159.0 (C), 148.5 (C), 144.1 (C), 139.5 (CH), 134.3 (CH), 131.2 (CH), 129.4 (C), 125.8 (CH), 124.8 (CH), 124.5 (CH), 123.7 (C), 106.1 (CH), 72.7 (C), 61.2 (CH_2_), 56.0 (CH_3_), 28.7 (3 × CH_3_), 14.7 (CH_3_). HRMS ESI_ACN. Calcd. for C_20_H_24_N_2_O_4_: 356.17361. Found: 356.17531. Anal. Calcd. for C_20_H_24_N_2_O_4_: C, 67.40; H, 6.79; N, 7.86. Found: C, 67.28; H, 6.82; N, 7.88.

### 4.2. Neuroprotection Analysis

#### 4.2.1. Primary Neuronal Cultures

Primary neuronal cultures were prepared from the cerebral cortex of rat embryos following a procedure previously described by our group [22]. Briefly, cell suspensions were prepared from cerebral cortices from E16 Sprague Dawley rat embryos and seeded on plastic multidishes precoated with 0.05 mg/mL poly-D-lysine at a density of 2.5 × 10^5^ cells/cm^2^. Cells were kept at 37 °C in a 6.5% CO_2_ atmosphere in high glucose DMEM medium supplemented with 15% heat-inactivated (56 °C, 30 min) fetal calf serum. After 24 h, culture medium was replaced by serum-free medium (DMEM/Ham’s F12, 1:1 vol/vol, 5 mg/mL glucose, 2 mM L-glutamine, and 1 mM sodium pyruvate, supplemented with 100 μg/mL transferrin, 100 μM putrescine, 30 nM sodium selenite, 20 nM progesterone, and 5 μg/mL insulin). In these experiments, 6- to 7-days in vitro (DIV) neuronal cultures were used, containing 90% β-tubulin isotype III-positive mature neurons, as described previously [23]. All procedures associated with animal experiments were authorized by the Animal Experimentation Ethics Committee of the Hospital Universitario Ramón y Cajal (Madrid, Spain).

#### 4.2.2. Experimental Ischemia in Neuronal Cultures and Treatments

Primary neuronal cultures were subjected to oxygen–glucose deprivation (OGD) to induce experimental ischemia [16]. Cells cultured for 6- to 7- DIV were washed and placed in glucose-free DMEM medium (previously bubbled with 95% N_2_/5% CO_2_ for 30 min) and kept in a humidified anaerobic chamber containing a gas mixture of 95% N_2_/5% CO_2_ at 37 °C for 4 h (OGD 4h). The control group was placed in glucose-supplemented DMEM medium and kept in the normoxic incubator for 4 h. After 4h of incubation under anoxic (OGD 4h) or normoxic (control) conditions, culture cells were quickly placed in serum-free normo-glycemic culture medium and let to reoxygenate. Compounds were formulated in 100% ethanol, added to culture medium with a final concentration ≤0.5% ethanol, and then cells were maintained in normoxic and normoglycemic conditions to recover for 24 h. Vehicle experimental group (R24h) included the same amount of formulation solution than the groups tested with compounds. The experimental procedure was blindly performed, assigning a random order to each group assayed. Compound assays were performed independently from four to eight times with different batches of cultures, and each experiment was run in quadruplicate.

#### 4.2.3. Cell Viability Assays

To assess the potential neuroprotective effect of the compounds against OGD, cell viability was evaluated by quantification of living, metabolically active cells, as determined by the colorimetric assay using the photometric reduction of 3-(4,5-dimethylthiazol-2-yl)-2,5-diphenyl tetrazolium bromide (MTT) (Roche) to a blue formazan product. MTT assay was performed after the 24-h-long recovery period—except for the OGD 4h group, for which MTT was added right after the end of the OGD period, with no recovery—by treating cells with 0.2 mg/mL MTT in the culture medium for 1.5 h at 37 °C in a 6.5% CO_2_ atmosphere. After incubation, cells were lysed with an equal volume of lysis solution (10 mM HCl, 10% SDS) overnight. Values were quantified by absorbance at 595 nm (reference 690 nm). Decreased MTT activity denotes impairment of mitochondrial function and is indicative of cell damage.

### 4.3. Statistical Analysis

Data from each treatment were independently analyzed and their averaged values were used for statistical analysis. The treatment information was kept concealed throughout the study. Data were represented as mean ± SE. Analysis of variance (ANOVA) was performed to compare the data between multiple concentrations, following post hoc test when analysis of variance was significant. Statistical significance level was set at α = 0.05 using Prism statistical software (GraphPad Software 5.0).

### 4.4. Computational Methods

All the calculations reported in this paper were performed with the Gaussian 09 suite of programs [30]. Electron correlation was partially taken into account using the hybrid functional usually denoted as B3LYP [31] in conjunction with the D3 dispersion correction suggested by Grimme et al. [32] using the standard double-ζ quality def2-SVP [33] basis set for all atoms. Geometries were fully optimized in solution without any geometry or symmetry constraints. All species were characterized by frequency calculations, [34] and have positive definite Hessian matrices. Frequency calculations were also used to determine the difference between the potential (E) and Gibbs (G) energies, G−E, which contains the zero-point, thermal and entropy energies. 

## Figures and Tables

**Table 1:** Neuroprotective activity of nitrones NXY-059 and QN23 on neuronal cultures after OGD ^a^.

Nitrone	Concentration (µM)	Neuroprotection (%)
**NXY-059**	250	52.90 ± 2.52
**QN23**	10	52.03 ± 1.75
	100	77.08 ± 2.24 ***
	250	65.88 ± 2.87 *

^a^ Neuroprotection was defined as the percentage of cell viability obtained when compared to the untreated R24 h value (0% neuroprotection), for which control group was set as 100%. * *p* < 0.05, *** *p* < 0.001, compared with nitrone NXY-059 (50 µM) by Newman–Keuls post-test after ANOVA. Data represented as mean ± SE. (Reprinted (adapted) with permission from *J. Med. Chem*. **2019**, *62*, 2184–2201. Copyright 2019 American Chemical Society [15]).

**Table 2:** Neuroprotective activity for NXY-059, QN23 and the most neuroprotective QNs, in neuronal cultures exposed to OGD ^a^.

Compound	Concentration (μM)	Neuroprotection (%)
**NXY-059**	100	41.93 ± 2.26
	250	51.26 ± 2.12
	500	45.64 ± 1.56
**QN23**	10	49.00 ± 0.89
	**100**	79.70 ± 0.98 **
	**250**	60.68 ± 1.29 **
	500	17.60 ± 0.77
**QN2**	1	33.66 ± 0.73
	10	33.26 ± 0.93
	100	39.98 ± 0.77
	**250**	62.43 ± 0.59 **
**QN4**	0.01	15.30 ± 0.43
	0.1	40.38 ± 0.35
	**1**	67.89 ± 1.44 **
	**10**	64.93 ± 0.57 **
	100	36.67 ± 0.44
**QN5**	1	24.50 ± 0.54
	10	42.41 ± 0.98
	100	41.71 ± 0.62
	**250**	60.19 ± 1.08 *
**QN7**	1	38.45 ± 0.62
	**10**	58.64 ± 1.11 *
	100	38.84 ± 1.10
	200	51.72 ± 0.99
**QN15**	0.1	32.42 ± 0.43
	1	48.09 ± 1.21
	10	46.81 ± 1.21
	**50**	70.26 ± 0.63 *

^a^ Neuroprotection was defined as the percentage to reach the control value, defined as 100%, from R24h value, defined as 0%. * *p* < 0.05 and ** *p* < 0.01, compared with NXY-059 (250 μM), by ANOVA and Dunnett’s post-test. Statistical significances of the data lower than 250 μM NXY-059 value were not shown.

## Data Availability

Data is contained within the article and supplementary material.

## Supplementary Materials

The following are available online at https://www.mdpi.com/article/10.3390/ph15111363/s1, 1. Synthesis of the intermediates for the preparation of QNs 1–16 and their NMR spectra. 2. Neuroprotection studies of QNs 1–16.
