# Supplementary material for: Improving the Efficacy of Quinolylnitrones for Ischemic Stroke Therapy, QN4 and QN15 as New Neuroprotective Agents after Oxygen–Glucose Deprivation/Reoxygenation-Induced Neuronal Injury
Source: Pharmaceuticals (Basel). 2022 Nov 7;15(11):1363. doi: 10.3390/ph15111363 (PMC9697404; doi:10.3390/ph15111363)
Supplement: Supplementary file 1 [file pharmaceuticals-15-01363-s001.zip › pharmaceuticals-1982760-supplementary.pdf]

Supplementary Material

Improving the Efficacy of Quinolylnitrones for Ischemic Stroke Therapy, QN4 and QN15 as New Neuroprotective Agents after Oxygen-Glucose Deprivation/Reoxygenation-Induced Neuronal Injury

José M. Alonso,[1,+] Alejandro Escobar-Peso,[2,†] Israel Fernández,[3,*] Alberto Alcázar,[2,*] and José Marco-Contelles [1]

[1] Laboratory of Medicinal Chemistry (IQOG, CSIC), C/Juan de la Cierva 3, 28006 Madrid, Spain

[2] Department of Research, Hospital Universitario Ramón y Cajal, IRYCIS, Ctra. Colmenar km 9.1, 28034 Madrid, Spain

[3] Departamento de Química Orgánica I and Centro de Innovación en Química Avanzada (ORFEO-CINQA), Facultad de Ciencias Químicas, Universidad Complutense de Madrid, 28040 Madrid, Spain

[†] These authors contributed equally to this work.

Content

1. Synthesis and NMR spectra of QNs 1-16……………………………………S2-S41

2. Neuroprotection studies of QNs 1-16………………………………………..S42-S44

1. Synthesis and NMR spectra of QNs

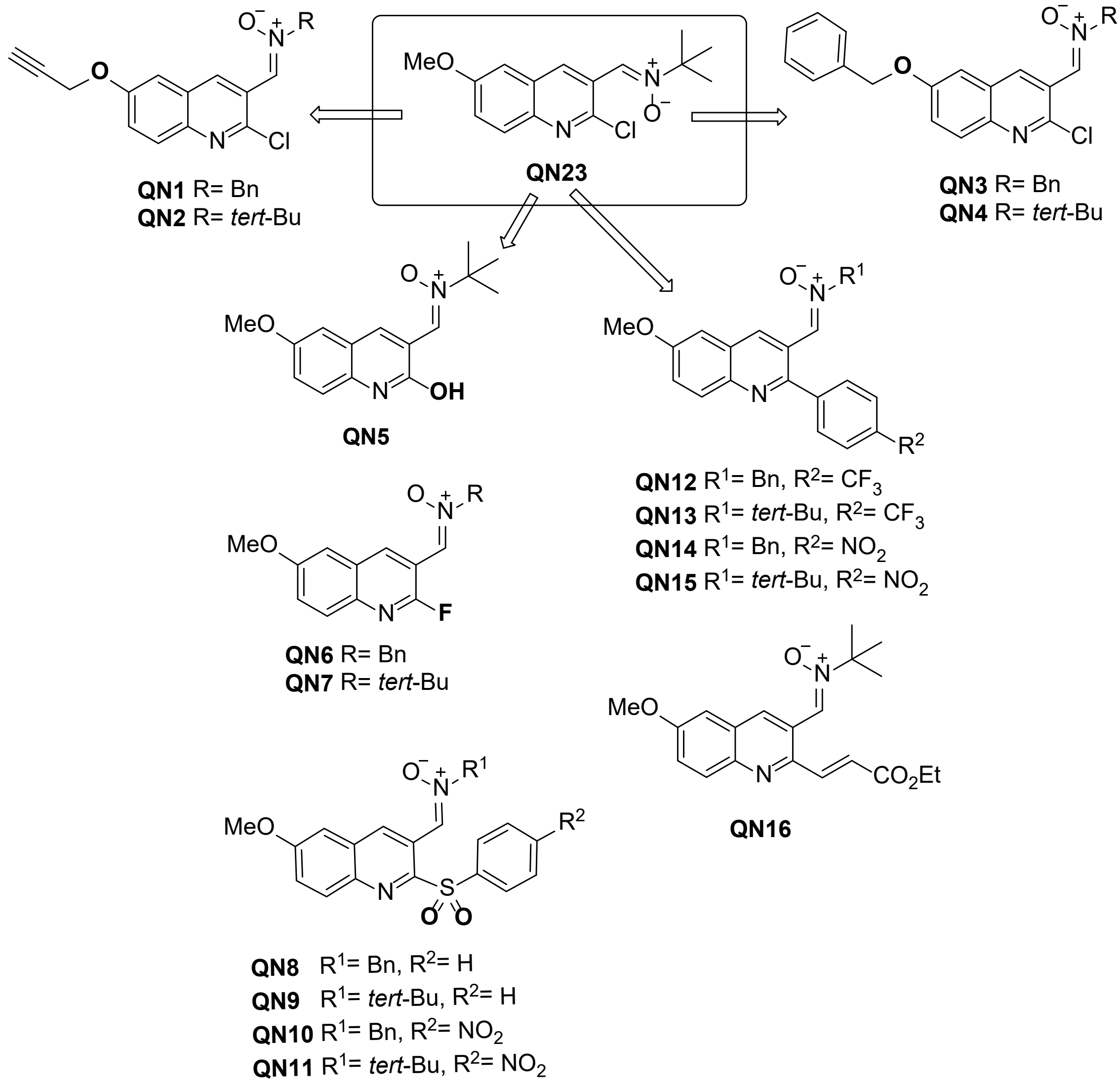

1.1. General methods. Melting points were determined on a Köffler apparatus, and are uncorrected. ^{1}H NMR and ^{13}C NMR spectra were recorded in $CDCl_3$ at 300 MHz and at 75 MHz, respectively, using solvent peaks [$CDCl_3$: 7.26 (D), 77.2 (C) ppm; D_2O: 4.60 ppm and DMSO-d_6: 2.49 (D), 39.52 (C) ppm] as internal reference. Mass spectra were recorded on a GC/MS spectrometer with an API-ES ionization source. Elemental analyses were performed at CNQO (CSIC, Spain). TLC were performed on silica F254 and detection by UV light at 254 nm or by charring with either ninhydrin, anisaldehyde or phosphomolybdic-H_2SO_4 reagents. Anhydrous solvents were used in all experiments. Column chromatography was performed on silica gel 60 (230 mesh).

1.2. General procedures. (A) General procedure for the synthesis of nitrones. A solution of the corresponding carbaldehyde (1 mmol), Na_2SO_4 (3 mmol), TEA (2 mmol) and the appropriate hydroxylamine hydrochloride (1.5 mmol) in THF/EtOH (5 mL, 4:1) was heated at 90 °C during 1-6 h under microwave irradiation (MWI). After that time, the solvent was evaporated and the crude mixture was purified on column chromatography using the indicated mixtures of solvents. **(B) General procedure for the synthesis of 6-methoxy-2-(arylsulfonyl)quinoline-3-carbaldehydes 7, 8**. A solution of 2-chloro-6-methoxyquinoline-3-carbaldehyde (**1**) (1 mmol), the corresponding arylsulfonyl chloride (1.2 mmol), and Na_2SO_3 (1.3 mmol) in H_2O (8 mL) was heated at 80 ˚C in an oil bath during 5 h. After that time, the mixture was diluted with DCM (15 mL), and extracted with $NaHCO_3$ (5% water solution), brine, and the organic phase was dried over $MgSO_4$. After filtration and evaporation of the solvent, the crude mixture was purified on column chromatography using the indicated mixtures of solvents. Arylsulfonyl quinolines were obtained as a mixtures of the expected products and starting material that we were unable to separate, and were directly used in the next step. **(C) General procedure for the synthesis of 6-methoxy-2-arylquinoline-3-carbaldehydes 10, 11**. A solution of commercial 2-iodo-6-methoxyquinoline-3-carbaldehyde (**9**) (1 mmol), the corresponding arylboronic acid (1.2 mmol), Pd_2dba_3 (5 mol%), PPh_3 (5 mol%), and *tert*-BuOK (1 mmol) in a mixture of toluene/H_2O (5 mL, 4:1) was refluxed during 16 h. Then, the solvent was evaporated and the residue was filtered through a path of Celite. Expected aldehydes were obtained as non-separable mixtures of compounds along with starting material, and therefore directly submitted to the next step without further purification.

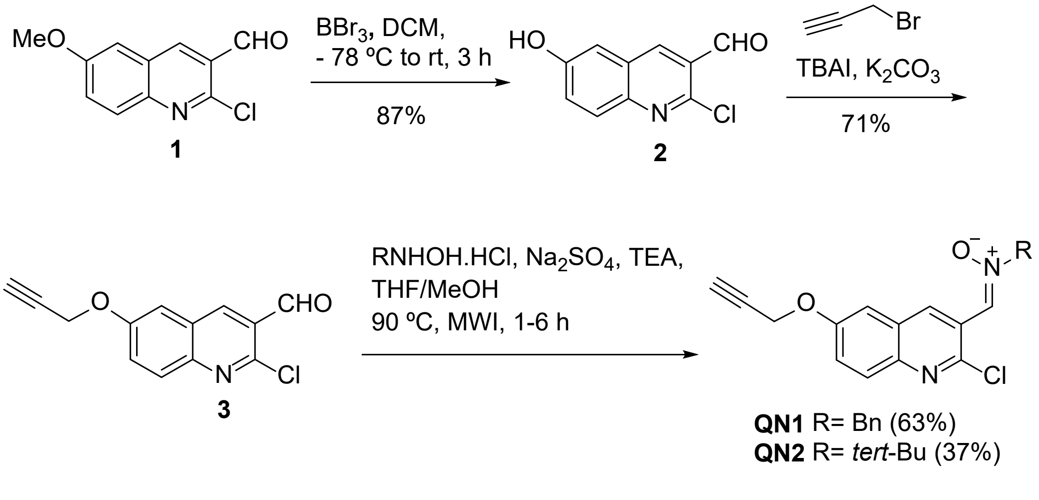

Scheme 1. Synthesis of 6-propargyloxy nitrones **QN1** and **QN2**.

2-Chloro-6-hydroxyquinoline-3-carbaldehyde (2). BBr_3 (7,9 mL, 1M in DCM, 3.5 equiv) was slowly added to a solution of commercial 2-chloro-6-methoxyquinoline-3-carbaldehyde **1** (500 mg, 2.262 mmol) in DCM (14 mL), cooled at - 78 °C. The reaction was left stirring at the same temperature for 30 min, and then, reach room temperature (rt) (20 °C) and stirred for additional 3 h. Then, water (5 mL) was carefully added at 0 °C, and the mixture was extracted using AcOEt/H_2O. The organic phases were collected and dried with brine and $MgSO_4$, the solvent was evaporated under reduced pressure to yield clean product **2** (405 mg, 87%), showing coincident analytical data to previously reported structure (Patel, A. B.; Premlata, K.; Kishor, C. *Catal. Lett.* **2014**, *144*, 1332-1338).

2-Chloro-6-(prop-2-yn-1-yloxy)quinoline-3-carbaldehyde (3). K_2CO_3 (319 mg, 2.318 mmol) in H_2O (3 mL) was added to a solution of compound **2** (240 mg, 1.159 mmol) , propargyl bromide (0.308 mL, 3.477 mmol), and TBAI (184 mg, 0.579 mmol) in THF (3 mL). The mixture was vigorously stirred and heated at 50 ˚C during 2 h, and then left 16 h at rt. After that time, water was added (5 mL), and the mixture was extracted with AcOEt (3x15 mL). The organic phases were collected and dried with brine and $MgSO_4$. After evaporation of the solvent, the crude mixture was purified on column chromatography (DCM/MeOH 30:1), to yield compound **3** as a yellow solid (200 mg, 81%): mp 74-5 °C; ^{1}H NMR (300 MHz, $CDCl_3$) δ 10.56 (s, 1H), 8.67 (s, 1H), 8.00 (dd, *J*= 9.2, 1.1 Hz, 1H), 7.56 (dd, *J*= 9.2, 2.8 Hz, 1H), 7.35 (dd, *J*= 2.8, 1.1 Hz, 1H), 4.85 (d, *J*= 3.0 Hz, 2H), 2.59 (t, *J*= 3.0 Hz, 1H); ^{13}C NMR (75 MHz, $CDCl_3$) δ 189.7 (CH), 156.5 (C), 147.0 (C), 145.2 (C), 140.3 (CH), 129.7 (CH), 127.7 (C), 126.9 (C), 126.3 (CH), 109.9 (CH), 79.0 (C), 78.8 (CH), 56.6 (CH_2); MS (EI) 245 (52) [M^+].

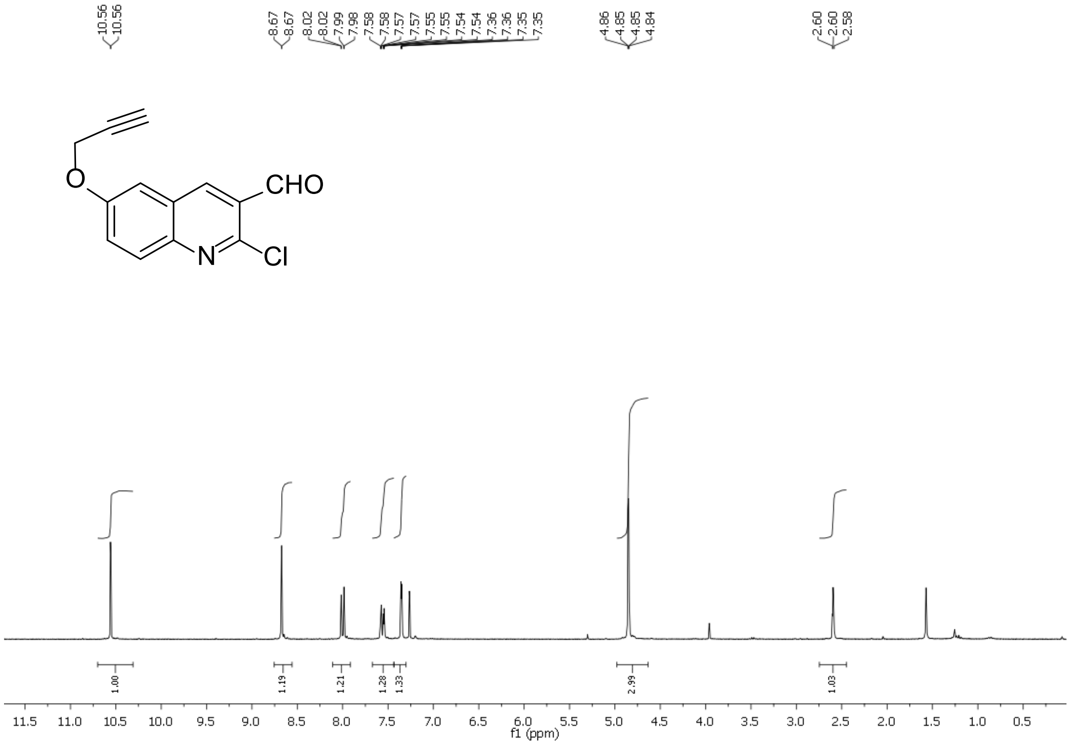

3 ^{1}H NMR

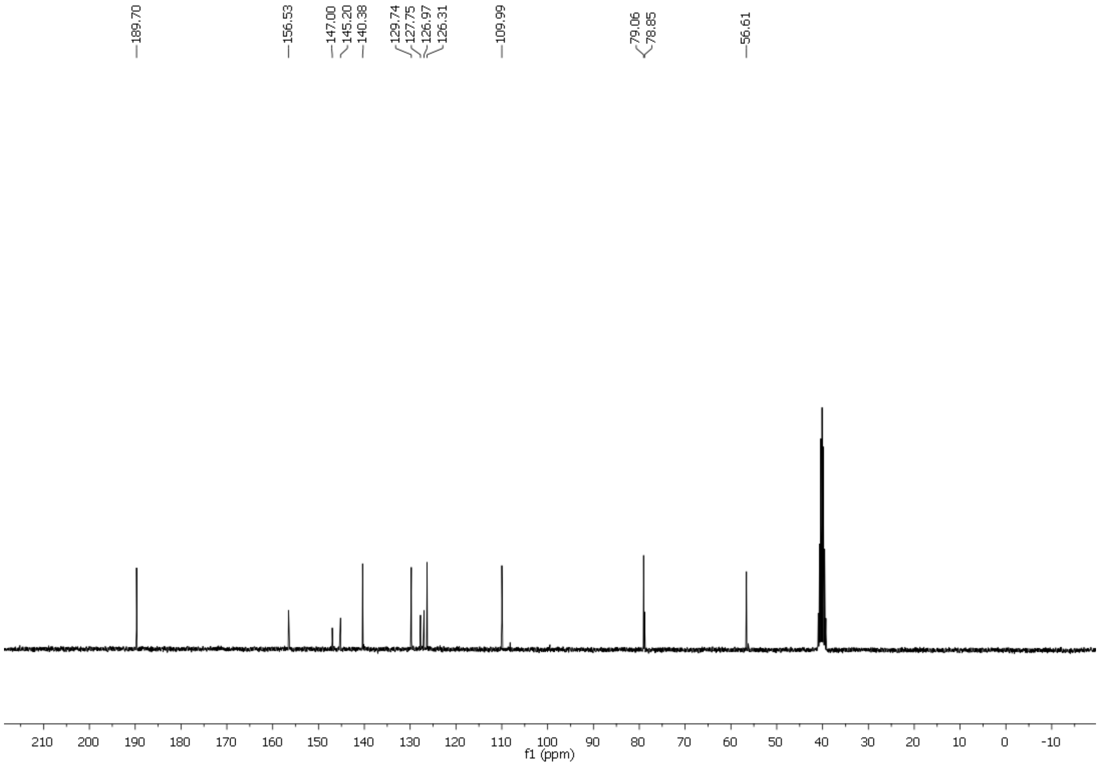

3 ^{13}C NMR

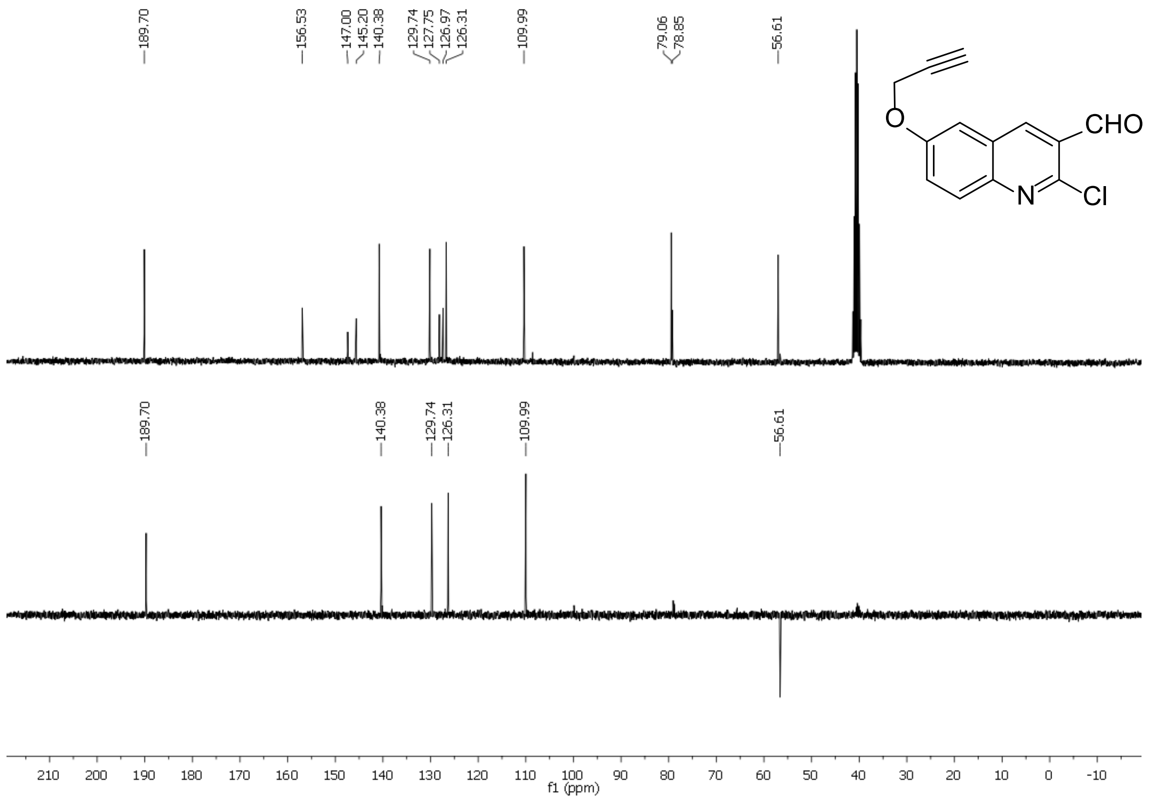

3 ^{13}C NMR vs DEPT

(*Z*)-*N*-Benzyl-1-(2-chloro-6-(prop-2-yn-1-yloxy)quinolin-3-yl)methanimine oxide (**QN1**). Following the **General procedure A**, the reaction of carbaldehyde **3** (100 mg, 0.408 mmol) with Na_2SO_4 (154 mg, 1.224 mmol), TEA (0.11 mL, 0.816 mmol) and *N*-benzylhydroxylamine hydrochloride (97 mg, 0.612 mmol) in THF/EtOH (5 mL, 4:1) for 1 h, gave after work-up and column chromatography (hexane/AcOEt, 4:1) nitrone **QN1** as a white solid (45 mg, 63%): mp 122-3 °C; ^{1}H NMR (300 MHz, $CDCl_3$) δ 10.14 (s, 1H), 8.03 (s, 1H), 7.79 (dd, *J*= 9.2, 2.7 Hz, 1H), 7.51-7.27 (m, 6H, H7, C_6H_5), 7.18 (d, *J*= 2.7 Hz, 1H), 5.09 (s, 2H, NCH_2Ph), 4.71 (d, *J*= 2.4 Hz, 2H, $OCH_2C{\equiv}CH$), 2.48 (t, *J* = 2.4 Hz, 1H, $OCH_2C{\equiv}CH$); ^{13}C NMR (75 MHz, $CDCl_3$) δ 156.6 (C), 146.5 (C), 143.6 (C), 136.6 (CH), 133.1 (C), 130.0 (CH), 129.8 (CH), 129.79 (CH), 129.74 (2 CH C2'), 129.56 (CH), 129.52 (CH), 128.2 (C), 124.7 (CH), 122.9 (C), 108.4 (CH), 78.1 (C), 76.7 (CH), 72.7 (CH_2), 56.5 (CH_2); MS (EI) 350.0 (1) [M^+]; 315.1 (100) [M^+-Cl]. HRMS (ESI_ACN). Calcd. for $C_{20}H_{15}ClN_2O_2$: 350.08221. Found: 350.08263. Anal. Calcd. for $C_{20}H_{15}ClN_2O_2 \cdot 1/3H_2O$: C, 67.32; H, 4.43; N, 7.85. Found: C, 67.34; H, 4.26; N, 7.91.

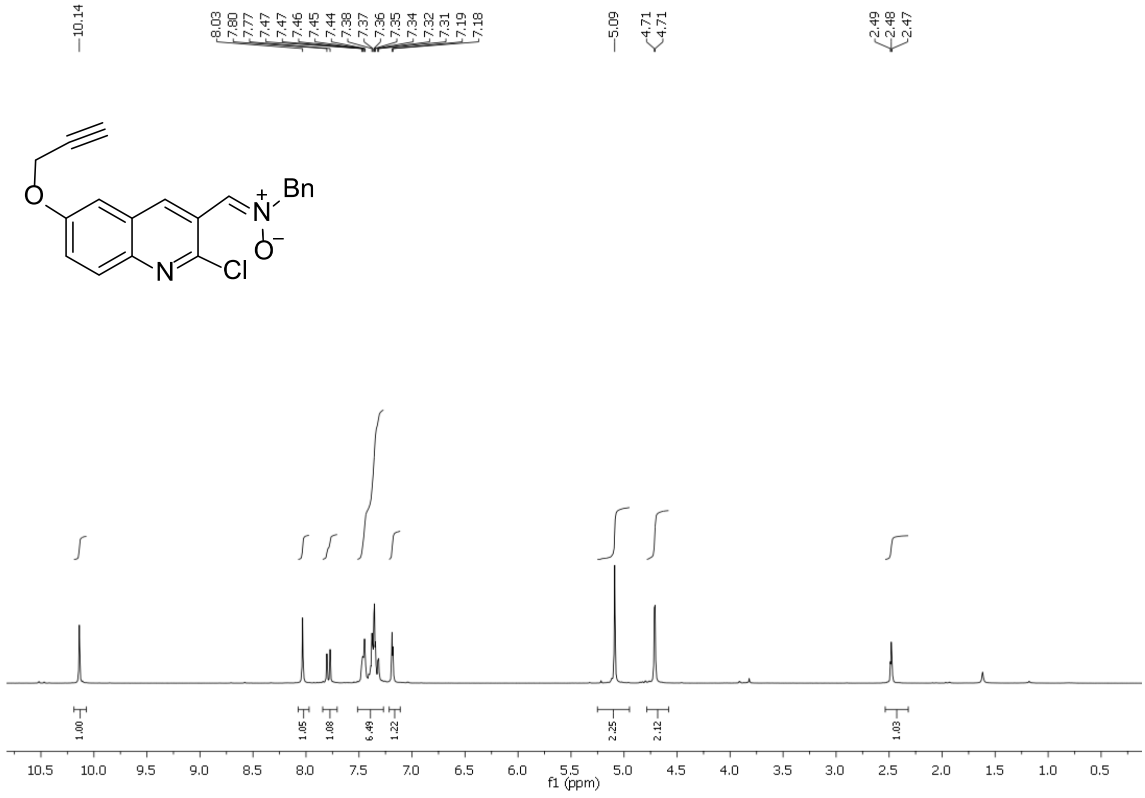

QN1 ^{1}H NMR

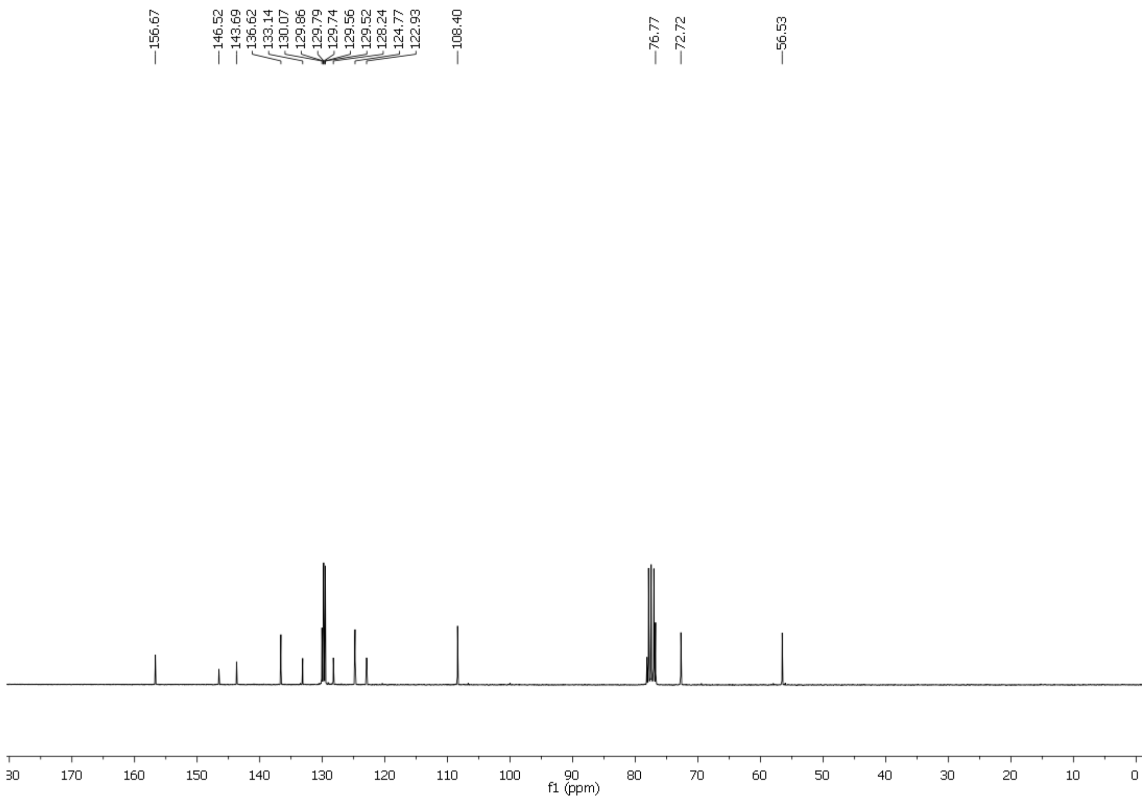

QN1 ^{13}C NMR

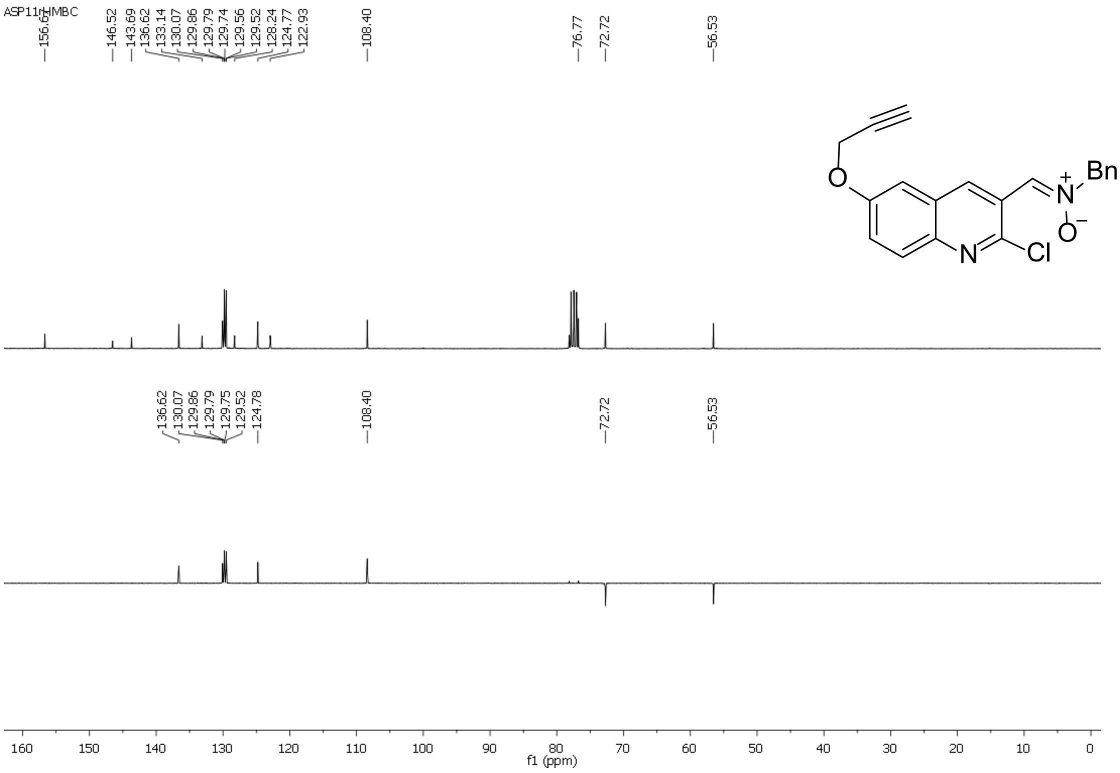

QN1 ^{13}C NMR vs DEPT NMR

(*Z*)-*N*-*tert*-Butyl-1-(2-chloro-6-(prop-2-yn-1-yloxy)quinolin-3-yl)methanimine oxide (QN2). Following the **General procedure A**, the reaction of carbaldehyde **3** (100 mg, 0.408 mmol) with Na_2SO_4 (154 mg, 1.224 mmol), TEA (0.11 mL, 0.816 mmol) and *N*-*tert*-butylhydroxylamine hydrochloride (77 mg, 0.612 mmol) in THF/EtOH (5 mL, 4:1) for 6 h, after work-up and column chromatography (hexane/AcOEt/DCM, 3:1:1) afforded compound **QN2** as a white solid (37 mg, 37%): mp 106-7 °C; ^{1}H NMR (300 MHz, $CDCl_3$) δ 10.27 (s, 1H), 8.21 (s, 1H), 7.82 (d, *J*= 9.2 Hz, 1H), 7.35 (dd, *J*= 9.2, 2.8 Hz, 1H), 7.24 (d, *J*= 2.8 Hz, 1H), 4.73 (d, *J*= 2.4 Hz, 2H), 2.49 (t, *J*= 2.4 Hz, 1H), 1.61 (s, 9H); ^{13}C NMR (75 MHz, $CDCl_3$) δ 156.6 (C), 147.1 (C), 143.5 (C), 136.3 (CH), 130.0 (CH), 128.4 (C), 125.8 (CH), 124.5 (CH), 123.4 (C), 108.4 (CH), 78.1 (C), 76.7 (CH), 72.9 (C), 56.5 (CH_2), 28.7 (3 x CH_3); MS (EI): 316.1 (2) [M^+]; 281.1 (32) [M^+-Cl]; 225.1 (100) [281.1–*tert*-Bu]. HRMS ESI_ACN. Calcd. for $C_{17}H_{17}ClN_2O_2$: 316.09786. Found: 316.09909. Anal. Calcd. for $C_{17}H_{17}ClN_2O_2$.½H_2O: C. 62.67; H, 5.57; N, 8.60, Found: C, 62.44; H, 5.29; N, 8.63.

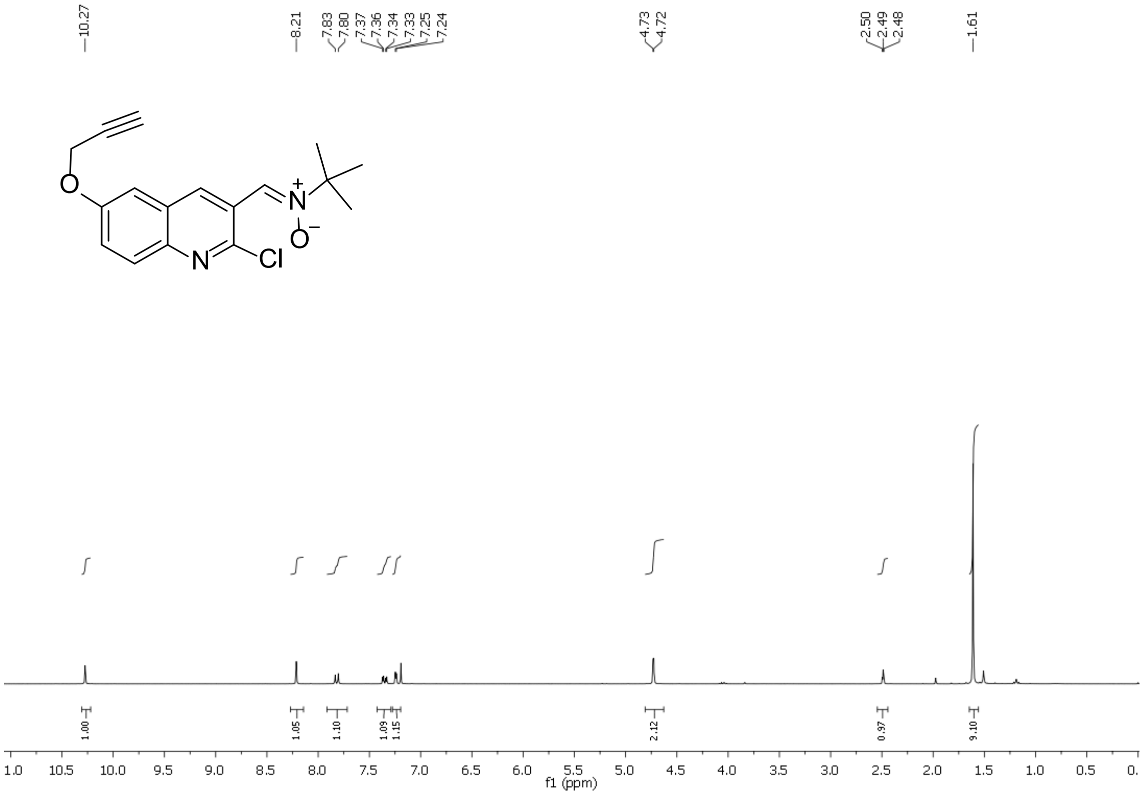

QN2 ^{1}H NMR

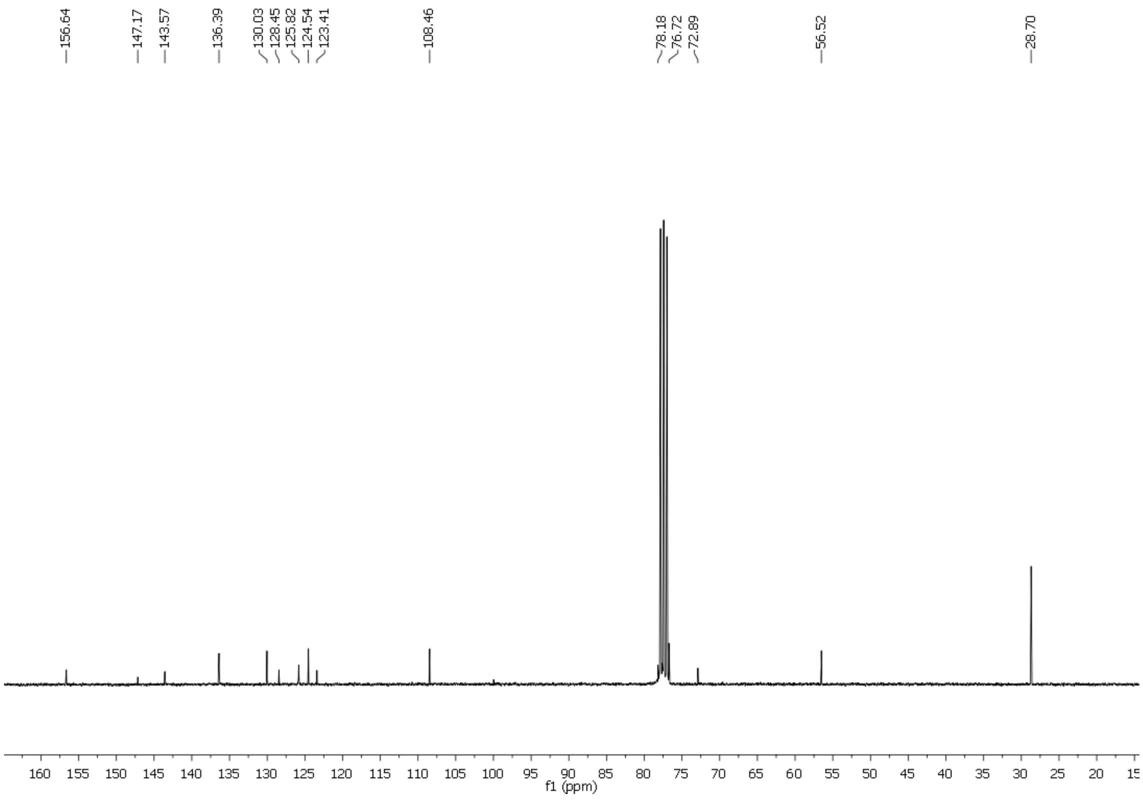

QN2 ^{13}C NMR

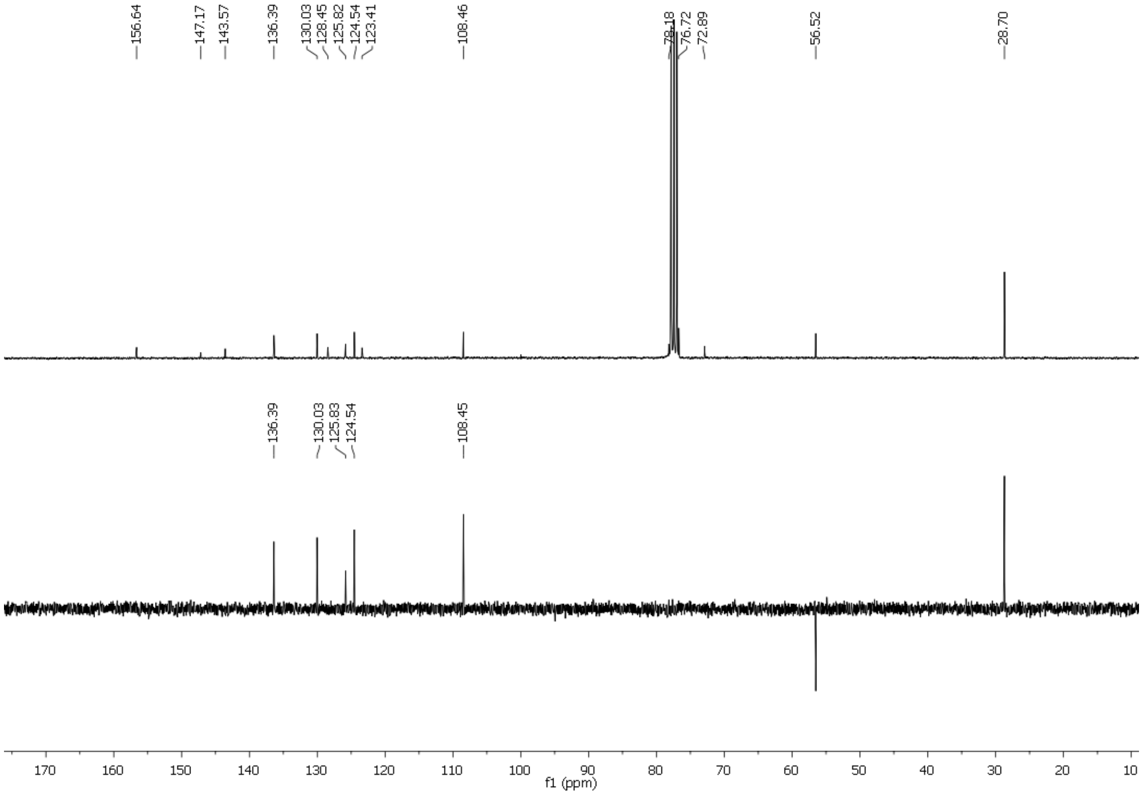

QN2 ^{13}C NMR vs DEPT NMR

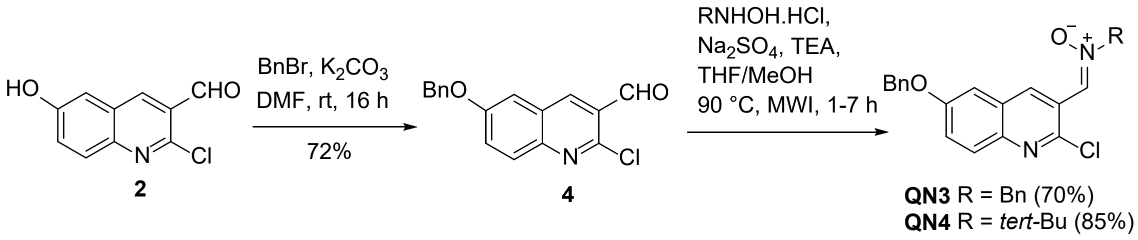

Scheme 2. Synthesis of 6-benzyloxyquinolin nitrones **QN3** and **QN4**.

6-(Benzyloxy)-2-chloroquinoline-3-carbaldehyde (4). K_2CO_3 (417 mg, 3.03 mmol) was added over a solution of compound **2** (314 mg, 1.51 mmol) in dry DMF (6.3 mL) at rt. After 10 min, BnBr (0.22 mL, 1.82 mmol) was added dropwise, and the mixture was stirred at rt for 16 h. Then, the solvent was evaporated to dryness, and the residue was dissolved in AcOEt (5 mL), and extracted with H_2O (3x10 mL). The organic phase was washed with brine and dry over $MgSO_4$. After filtration and evaporation of the solvent, the crude mixture was purified by column chromatography (hexane/AcOEt, 1:1) to yield

pure product **4** showing coincident analytical and spectroscopic data for those reported in literature (Bai, X.; Chen, Y.; Liu, Z.; Zhang, L.; Feng, B. Chem. Biodiv. **2019**, *16*, e1900056).

(*Z*)-*N*-Benzyl-1-(6-(benzyloxy)-2-chloroquinolin-3-yl)methanimine oxide (QN3). Following the **General procedure A**, the reaction of carbaldehyde **4** (70 mg, 0.236 mmol) with *N*-benzylhydroxylamine hydrochloride (56 mg, 0.353 mmol), Na_2SO_4 (89 mg, 0.708 mmol) and TEA (65 μL, 0.472 mmol) in THF/EtOH (3:0.5 mL), for 1 h, after work-up and column chromatography (hexane/AcOEt, 2:1), gave nitrone **QN3**, obtained as a white solid (65 mg, 70%): mp 182-3 °C; ^{1}H NMR (300 MHz, $CDCl_3$) δ 10.09 (s, 1H), 8.02 (s, 1H), 7.79 (d, *J*= 9.2 Hz, 1H), 7.53-7.23 (m, 11H, H7, 2 C_6H_5), 7.11 (d, *J*= 2.8 Hz, 1H), 5.08 (s, 4H); ^{13}C NMR (75 MHz, $CDCl_3$) δ 157.9 (C), 146.2 (C), 143.5 (C), 136.5 (CH), 133.1 (2 C), 130.0 (2 CH), 129.94 (CH), 129.78 (2 CH), 129.75 (CH), 129.53 (2 CH), 129.1 (2 CH), 128.7 (CH), 128.4 (C), 127.9 (CH), 125.1 (CH), 122.8 (C), 108.0 (CH), 72.7 (CH_2), 70.8 (CH_2); MS (EI): 402.1 (5) [M^+]; 367 (100) [M^+-Cl]. HRMS ESI_ACN. Calcd. for $C_{24}H_{19}ClN_2O_2$: 402.11351. Found: 402.11419. Anal. Calcd. for $C_{24}H_{19}ClN_2O_2$: C, 70.50; H, 4,85; N, 6.85. Found: C, 70.39; H, 4.79; N, 6.81.

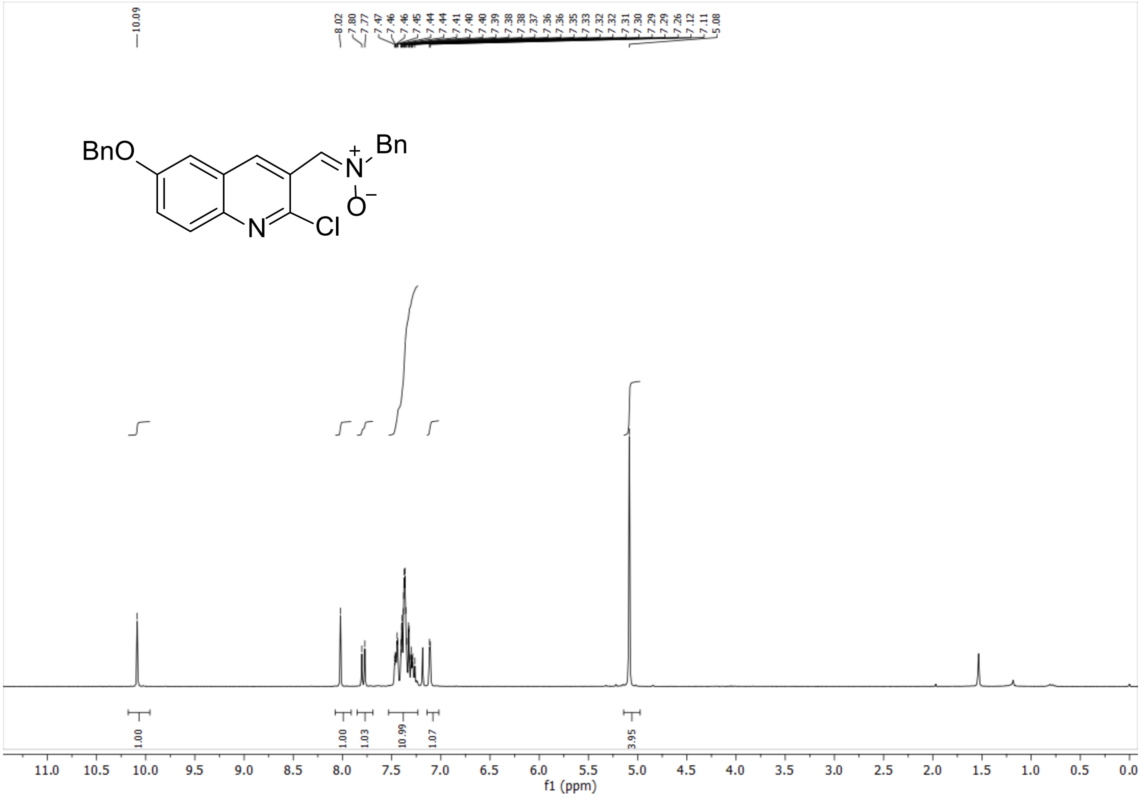

QN3 ^{1}H NMR

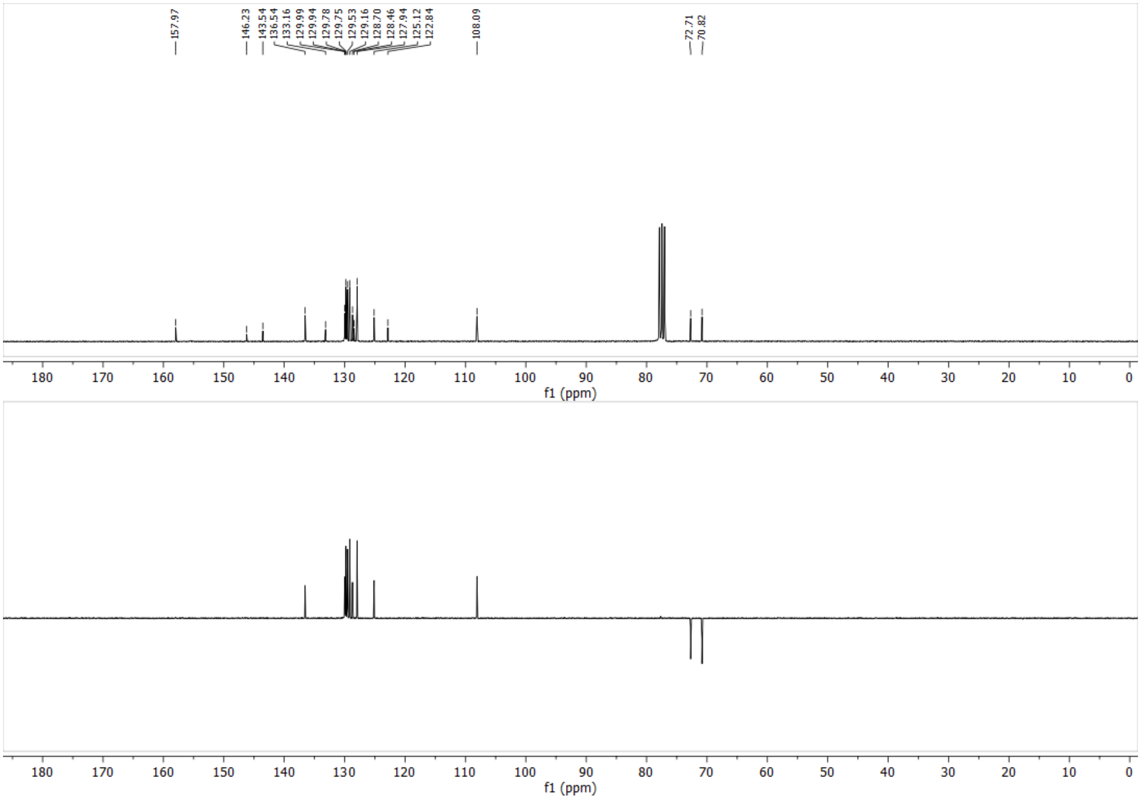

QN3 ^{13}C NMR vs DEPT

(*Z*)-*N-tert*-Butyl-1-(6-(benzyloxy)-2-chloroquinolin-3-yl)methanimine oxide (QN4). Following the **General procedure A**, the reaction of carbaldehyde **4** (70 mg, 0.236 mmol) with *N-tert*-butylhydroxylamine hydrochloride (44 mg, 0.353 mmol), Na_2SO_4 (89 mg, 0.708 mmol) and TEA (65 µL, 0.472 mmol) in THF/EtOH (3:0.5 mL), for 6 h, gave after work-up and column chromatography (hexane/AcOEt, 7:3) nitrone **QN4**, obtained as a white solid (73 mg, 85%): mp 205-6 °C; ^{1}H NMR (300 MHz, $CDCl_3$) δ 10.19 (s, 1H), 8.19 (s, 1H), 7.80 (d, *J*= 9.1 Hz, 1H), 7.45-7.24 (m, 6H), 7.13 (d, *J*= 2.8 Hz, 1H), 5.10 (s, 2H), 1.60 (s, 9H); ^{13}C NMR (75 MHz, $CDCl_3$) δ 157.9 (C), 146.8 (C), 143.3 (C), 136.5 (CH), 136.2 (C), 129.9 (CH), 129.1 (2 CH), 128.67 (CH), 128.63 (C), 127.8 (2 CH), 125.8 (CH), 124.8 (CH), 123.3 (C), 108.1 (CH), 72.8 (C), 70.7 (CH_2), 28.7 (3 x CH_3); MS (EI): 367.1 (2) [M^+]; 333.1 (37) [M^+-Cl]. HRMS ESI_ACN. Calcd. for $C_{21}H_{21}ClN_2O_2$: 368.12916. Found: 368.12987. Anal. Calcd. for $C_{21}H_{21}ClN_2O_2$: C, 68.35; H, 5,74; N, 7.59. Found: C, 68.22; H, 5.74; N, 7.58.

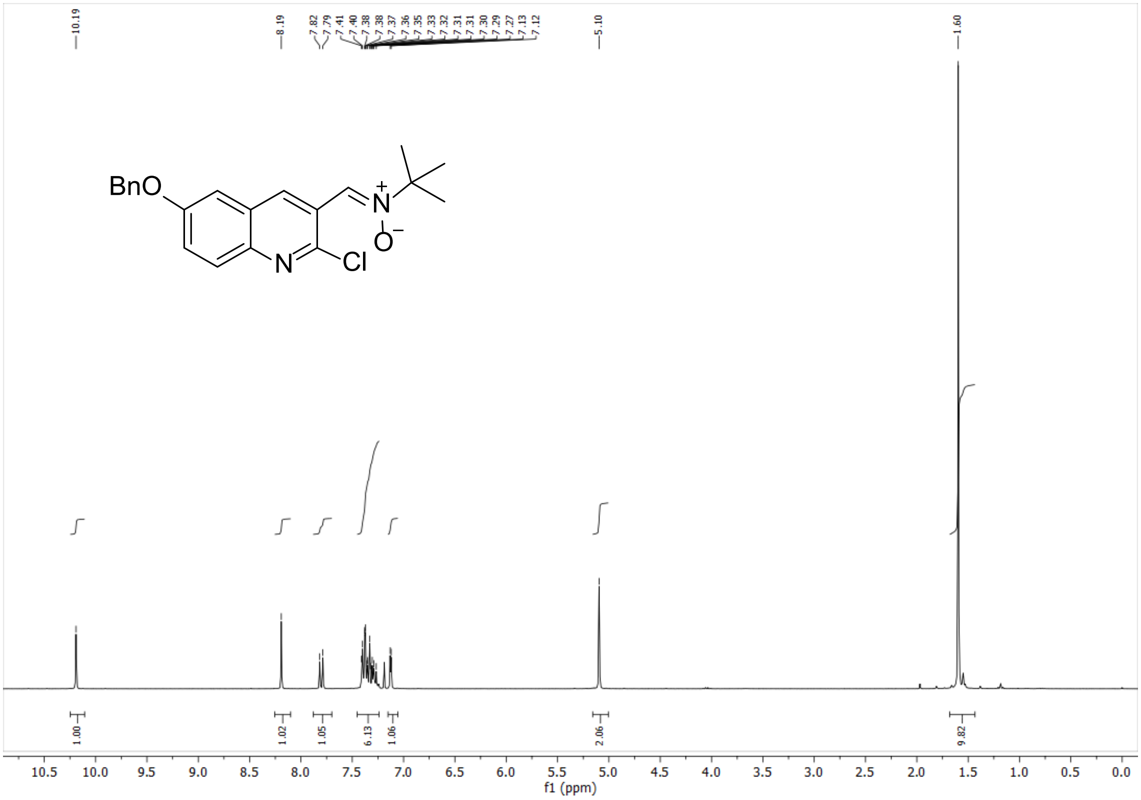

QN4 ^{1}H NMR

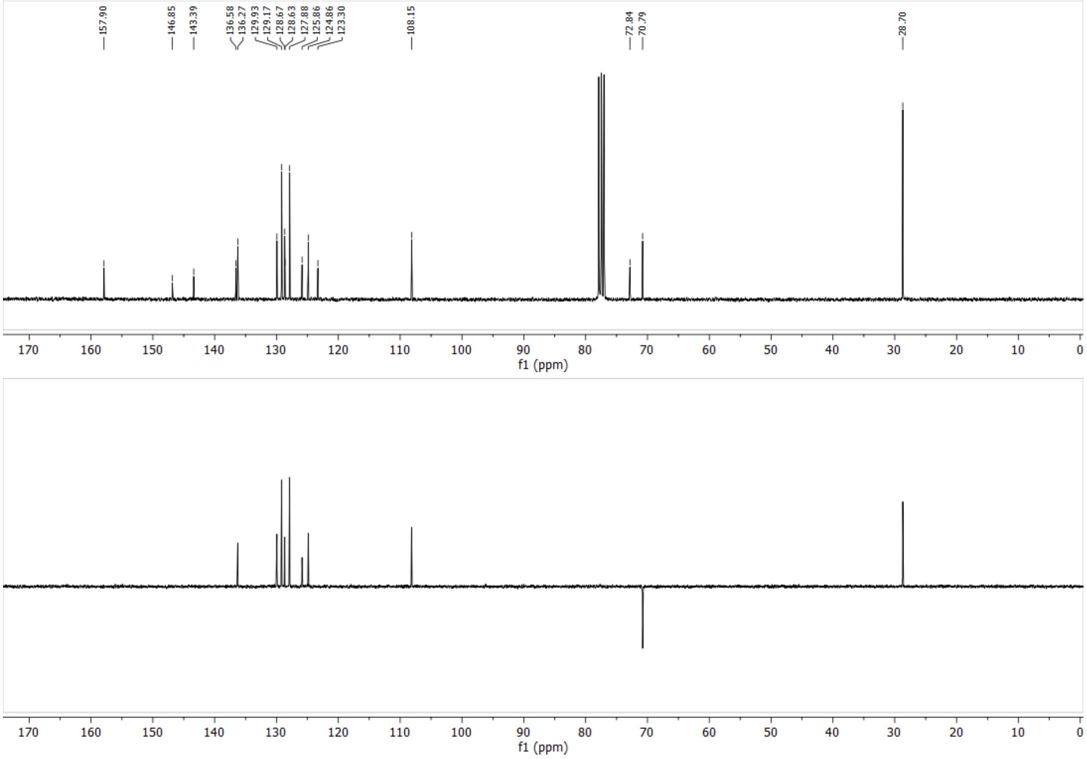

QN4 ^{13}C NMR vs DEPT

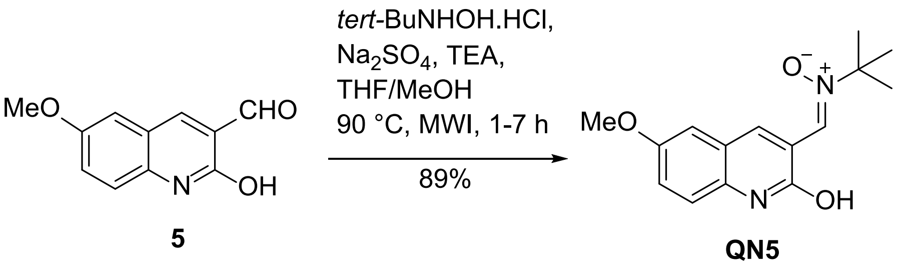

Scheme 3. Synthesis of 2-hydroxynitrone **QN5**.

(*Z*)-*N*-*tert*-Butyl-1-(2-hydroxy-6-methoxyquinolin-3-yl)methanimine oxide (**QN5**). Following the **General procedure A**, the reaction of commercial compound **5** (50 mg, 0.153 mmol) with *N*-*tert*-butylhydroxylamine hydrochloride (30 mg, 0.229 mmol), Na_2SO_4 (60 mg, 0.459 mmol) and TEA (42 , 0.306 mmol) in THF (2 mL), for 6 h, after work-up and column chromatography (DCM/AcOEt, 1:2), gave nitrone **QN5** as a yellow solid (40 mg, 89%): mp 172-3 °C; ^{1}H NMR (300 MHz, $CDCl_3$) δ 11.77 (br s, 1H), 10.06 (s, 1H), 8.26 (s, 1H), 7.28-7.15 (m, 1H), 7.09 (dd, *J*= 8.9, 2.6 Hz, 1H), 6.99 (d, *J*= 2.6 Hz, 1H), 3.76 (s, 3H, OCH_3), 1.59 (s, 9H); ^{13}C NMR (75 MHz, $CDCl_3$) δ 162.7 (C), 155.8 (C), 137.8 (CH), 133.1 (C), 125.3 (CH), 122.7 (C), 121.6 (CH), 121.4 (C), 117.1 (CH), 110.3 (CH), 72.1 (C), 56.0 (CH_3), 28.7 (3 x CH_3); MS (EI): 274.1 (62) [M^+]. HRMS ESI_ACN. Calcd. for $C_{15}H_{18}N_2O_3$: 274.13174. Found: 274.13130. Anal. Calcd for $C_{15}H_{18}N_2O_3$: C, 65.68; H, 6,61; N, 10.21. Found: C, 65.39; H, 6.58; N, 9.97.

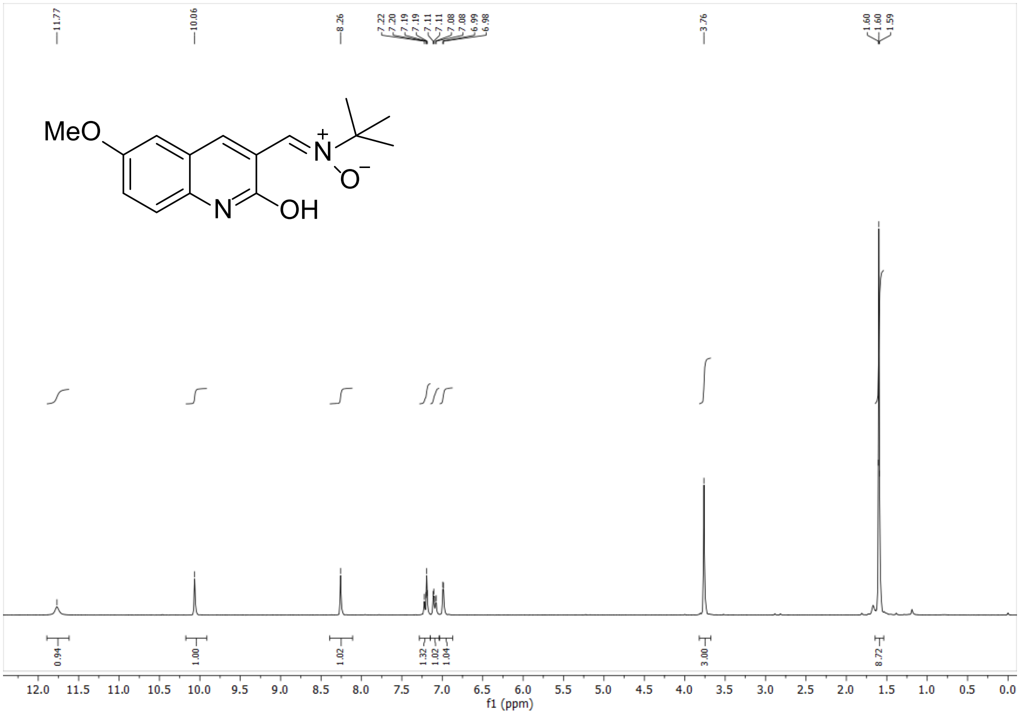

QN5 ^{1}H NMR

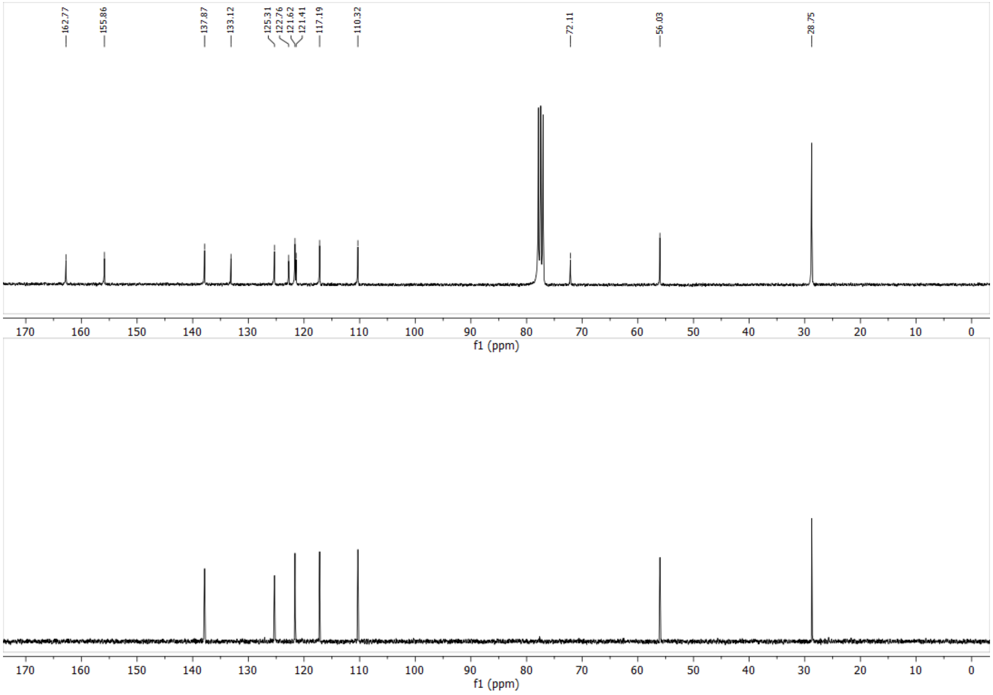

QN5 ^{13}C NMR vs DEPT

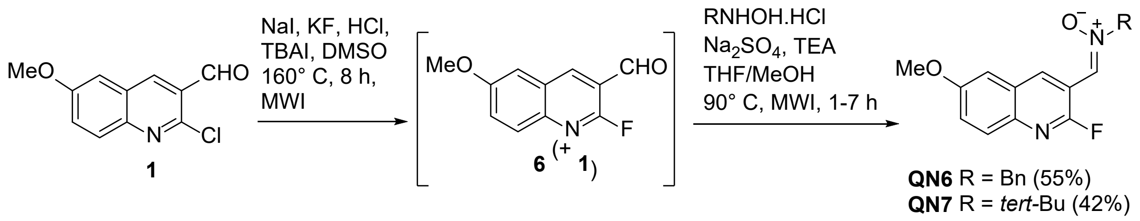

Scheme 4. Synthesis of 2-fluoroquinolin nitrones **QN6** and **QN7**.

(*Z*)-*N*-Benzyl-1-(6-(methoxy)-2-fluoroquinolin-3-yl)methanimine oxide (QN6). A solution of 2-chloro-6-methoxyquinoline-3-carbaldehyde (**1**) (70 mg, 0.317 mmol), NaI (190 mg, 1.267 mmol), KF (147 mg, 2.536 mmol), and catalytic amount of HCl and TBAI in DMSO (1 mL), was heated at 160 ^{0}C for 8 h under MWI. Then, the mixture was diluted with AcOEt and washed with H_2O (3x10 mL). The organic phase was washed with brine and dry over $MgSO_4$. After evaporation of the solvent, the crude mixture was purified by column chromatography (hexane/AcOEt, 7:3) to yield a mixture of major 2-fluoro-6-methoxyquinoline-3-carbaldehyde (**6**) {^{1}H NMR (300 MHz, $CDCl_3$) δ 10.32 (s, 1H, CHO), 8.35 (s, 1H, H4), 7.88 (dd, *J*= 9.2, 1.0 Hz, 1H, H8), 7.43 (dd, *J*= 9.2, 2.8 Hz, 1H), 7.10 (dd, *J*= 2.8, 1.0 Hz, 1H), 3.93 (s, 3H, OCH_3); MS (EI): 206.1 (100) [M+1]} and minor starting material **1**, that we could not separate (55 mg of a mixture of compounds 9/1 in 1:0.3 ^{1}H NMR ratio. Compound **6**, obtained in 65% based on the ^{1}H NMR spectrum, was used in the next steps without further purification.

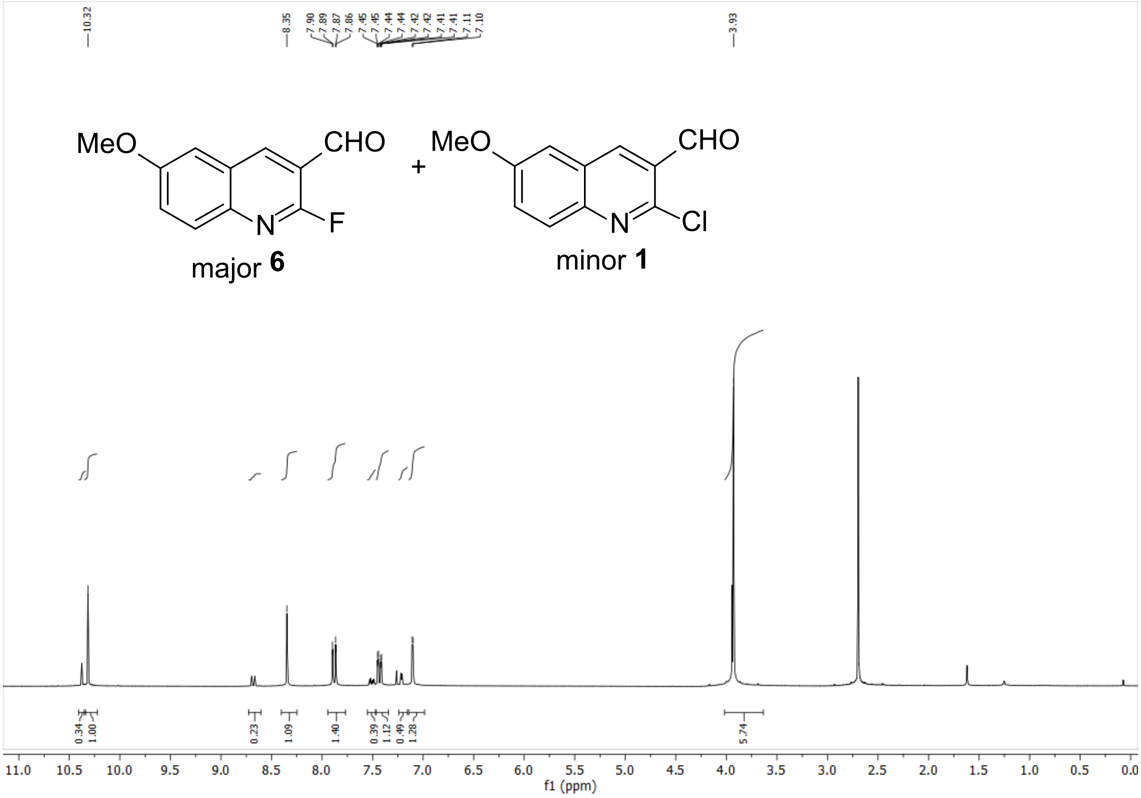

6 + 1 ^{1}H NMR

Following the **General procedure A**, the reaction of the mixture **6**+**1** (60 mg) with *N*-benzylhydroxylamine hydrochloride (70 mg, 0.439 mmol), Na_2SO_4 (111 mg, 0.879 mmol) and TEA (81 μL, 0.586 mmol) in THF/EtOH (3:0.5 mL), for 1 h, after work-up and column chromatography (hexane/AcOEt/Et_2O, 4:1:1) gave nitrone **QN6** as a white solid (50 mg, 55%): mp 197-9 °C; ^{1}H NMR (300 MHz, $CDCl_3$) δ 10.08 (d, *J*= 9.8 Hz, 1H), 7.77 (s, 1H), 7.69 (d, *J*= 9.1 Hz, 1H), 7.49-7.22 (m, 6H), 7.08 (d, *J*= 2.8 Hz, 1H), 5.07 (s, 2H), 3.82 (s, 3H); ^{13}C NMR (75 MHz, $CDCl_3$) δ 158.3 (C, d, *J*= 2.2 Hz), 156.5 (d, *J* = 242 Hz, C), 140.1 (C, d, *J*= 68.4 Hz), 138.51 (d, *J* = 3.4 Hz, CH), 133.1 (C), 129.7 (2 CH), 129.5 (2 CH), 129.2 (CH), 128.4 (C), 126.6 (CH), 124.4 (CH), 114.1 (C, d, *J*= 28.5 Hz), 107.2 (CH), 72.4 (CH_2), 56.0 (CH_3); MS (EI): 310.1 (100) [M^+]. HRMS ESI_ACN Calcd. for $C_{18}H_{15}FN_2O_2$: 310.11176. Found: 310.11076. Anal. Calcd for $C_{18}H_{15}FN_2O_2$: C, 69.67; H, 4,87; N, 9.03. Found: C, 69.52; H, 5.01; N, 9.04.

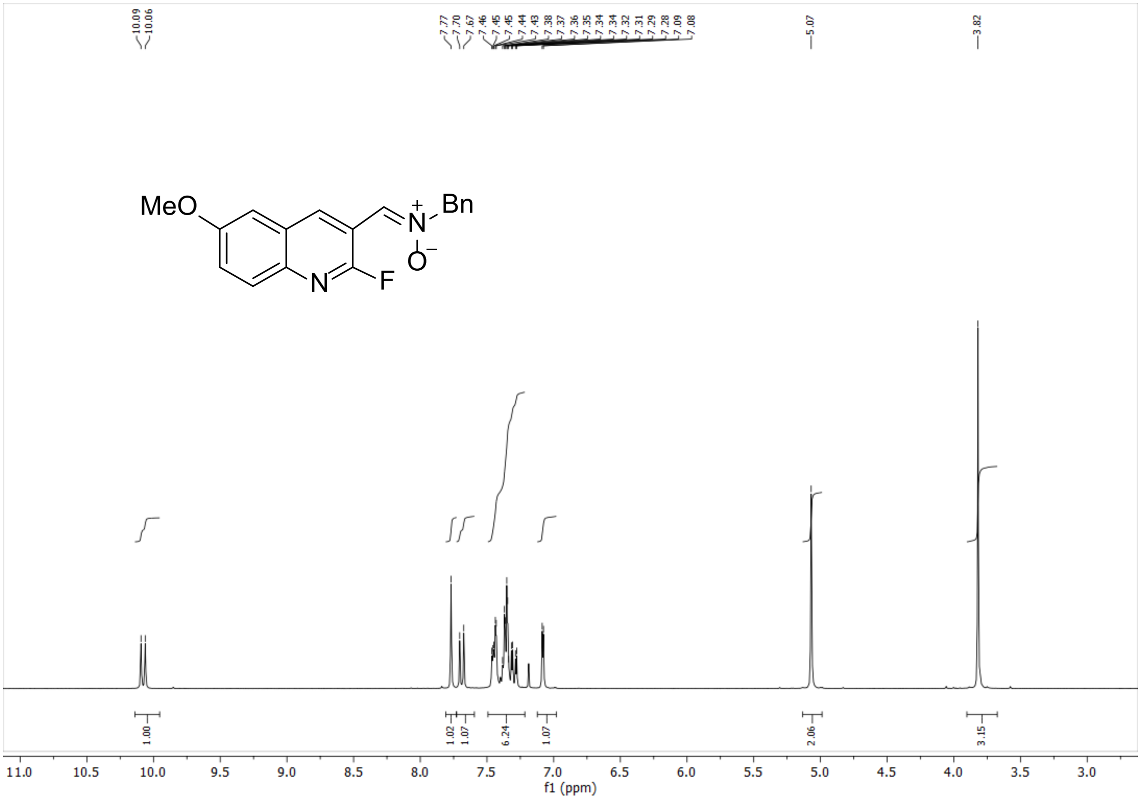

QN6 ^{1}H NMR

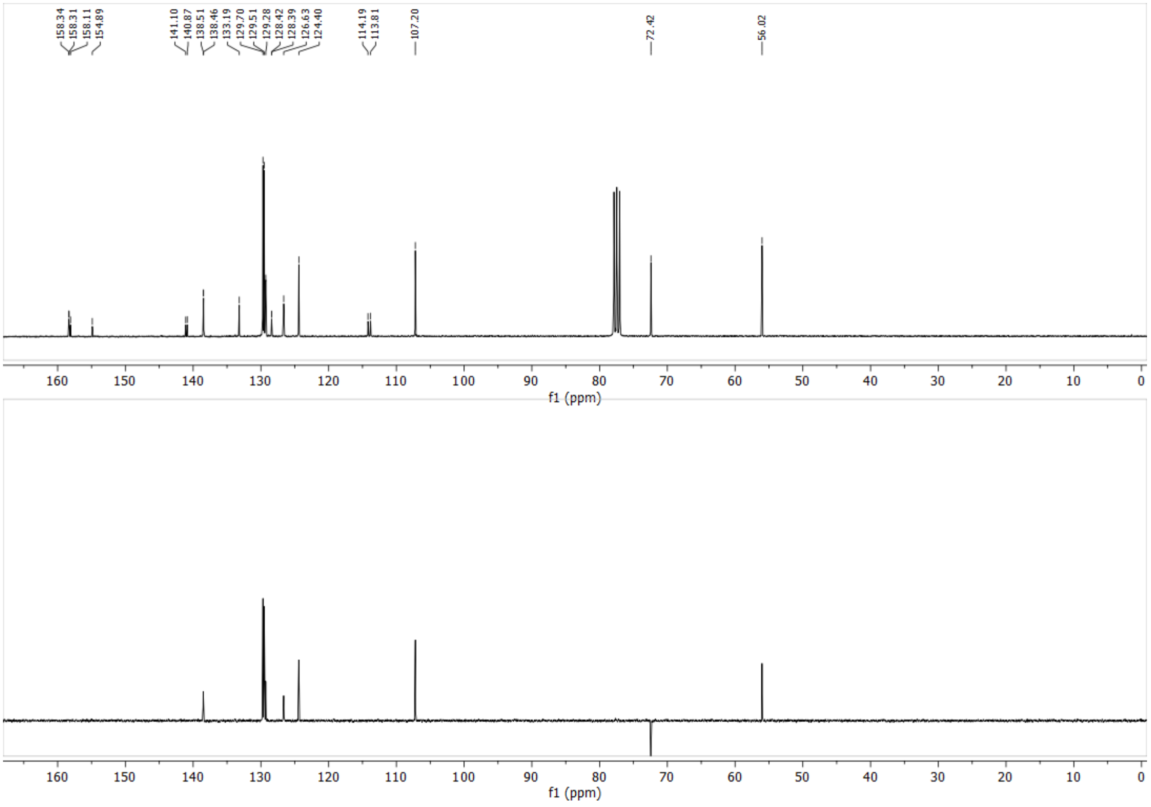

QN6 ^{13}C NMR vs DEPT

(*Z*)-*N-tert*-Butyl-1-(6-(methoxy)-2-fluoroquinolin-3-yl)methanimine oxide (QN7). Following the **General procedure A**, the reaction of mixture **6+1** (100 mg) with *N-tert*-butylhydroxylamine hydrochloride (88 mg, 0.731 mmol), Na_2SO_4 (184 mg, 1.461 mmol) and TEA (135 µL, 0.974 mmol) in THF (4 mL), for 6 h, after work-up and column chromatography (hexane/AcOEt/MeOH, 10:5:1) gave nitrone **QN7**, as a white solid (57 mg, 42%): mp 156-7 °C; ^{1}H NMR (300 MHz, $CDCl_3$) δ 10.26 (d, *J*= 9.8 Hz, 1H), 7.96 (s, 1H), 7.77 (d, *J*= 9.2 Hz, 1H), 7.37 (dd, *J*= 9.2, 2.8 Hz, 1H), 7.18 (s, 1H), 3.89 (s, 3H), 1.65 (s, 9H); ^{13}C NMR (75 MHz, $CDCl_3$) δ 158.2 (C, d, *J*= 2.0 Hz), 156.8 (d, *J* = 239 Hz, C), 140.7 (C, d, *J*= 17.4 Hz), 138.2 (d, *J* = 3.5 Hz, CH), 129.2 (CH), 128.5 (C), 124.0 (C), 122.4 (CH), 114.5 (C, d, *J*= 27.7 Hz), 107.2 (CH), 72.6 (C), 55.9 (CH_3), 28.7 (3 x CH_3); MS (EI): 276.1 (57) [M^+]. HRMS ESI_ACN. Calcd. for $C_{15}H_{17}FN_2O_2$: 276.12741. Found: 276.12834. Anal. Calcd. $C_{15}H_{17}FN_2O_2$: C, 63.15; H, 6.36; N, 9.82. Found: C, 63.35; H, 6.15; N, 9.74.

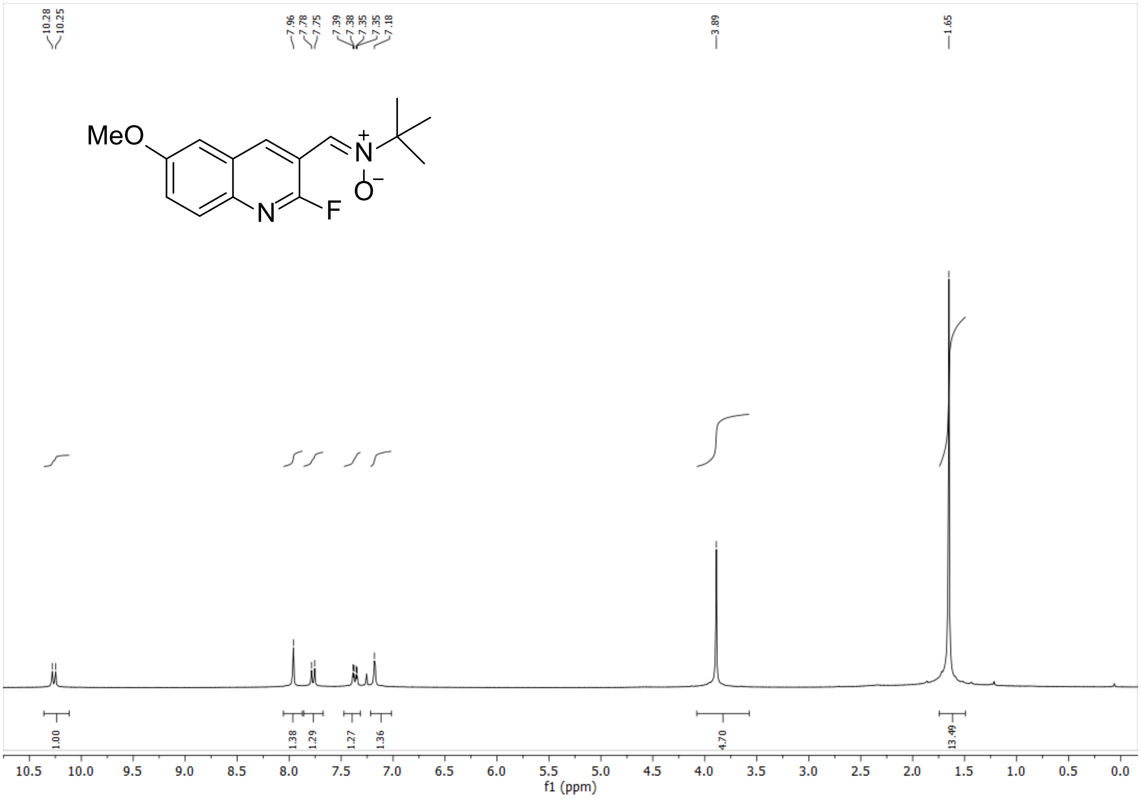

QN7 ^{1}H NMR

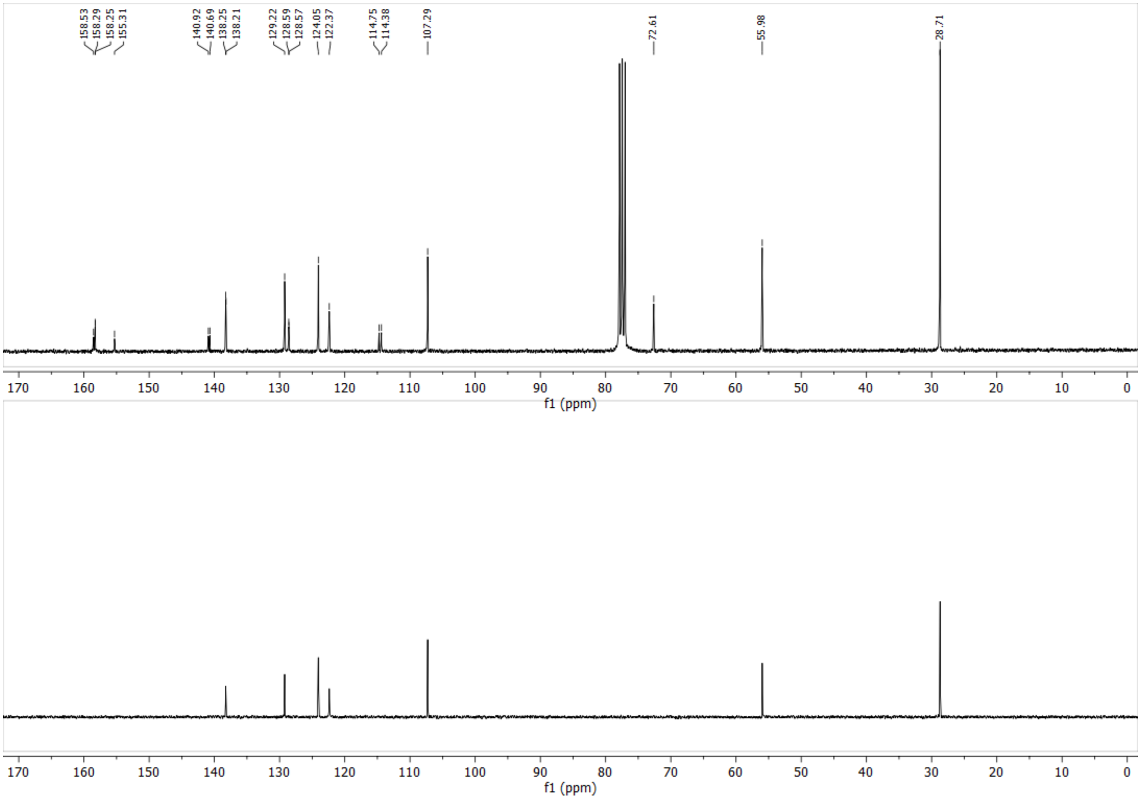

QN7 ^{13}C NMR vs DEPT

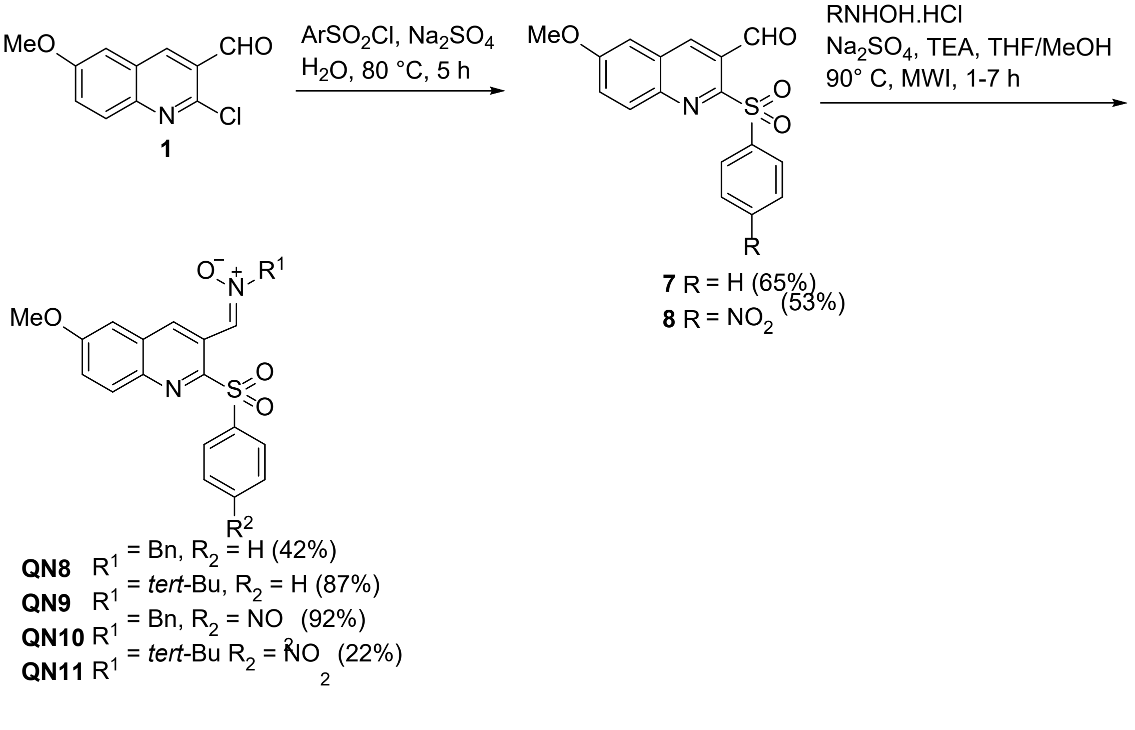

Scheme 5. Synthesis of 2-arylsulfonylquinolin nitrones **QNs 8-11**.

(*Z*)-*N*-Benzyl-1-(6-methoxy-2-(phenylsulfonyl)quinolin-3-yl)methanimine oxide (QN8). Following the **General procedure B**, the reaction of 2-chloro-6-methoxyquinoline-3-carbaldehyde (**1**) (42 mg, 0.189 mmol) with phenylsulfonyl chloride (72 µL, 0.567 mmol) and Na_2SO_3 (71 mg, 0.567 mmol) in H_2O (0.7 mL), after purification on column chromatography (hexane/AcOEt, 2:1) gave a mixture of unreacted compound **1** and 6-methoxy-2-(phenylsulfonyl)quinoline-3-carbaldehyde (**7**) (40 mg, 65%, yield estimated on the ^{1}H NMR basis spectrum), as a thick gum {^{1}H NMR (300 MHz, $CDCl_3$) (for major compound **7**) δ 11.09 (s, 1H), 8.69 (s, 1H), 8.12-7.96 (m, 2H), 7.85 (d, *J*= 9.2 Hz, 1H), 7.64-7.48 (m, 3H), 7.42 (dd, *J*= 9.2, 2.7 Hz, 1H), 7.14 (d, *J*= 2.7 Hz, 1H), 3.89 (s, 3H); MS (EI): 327.1 (5) [M^+]}.

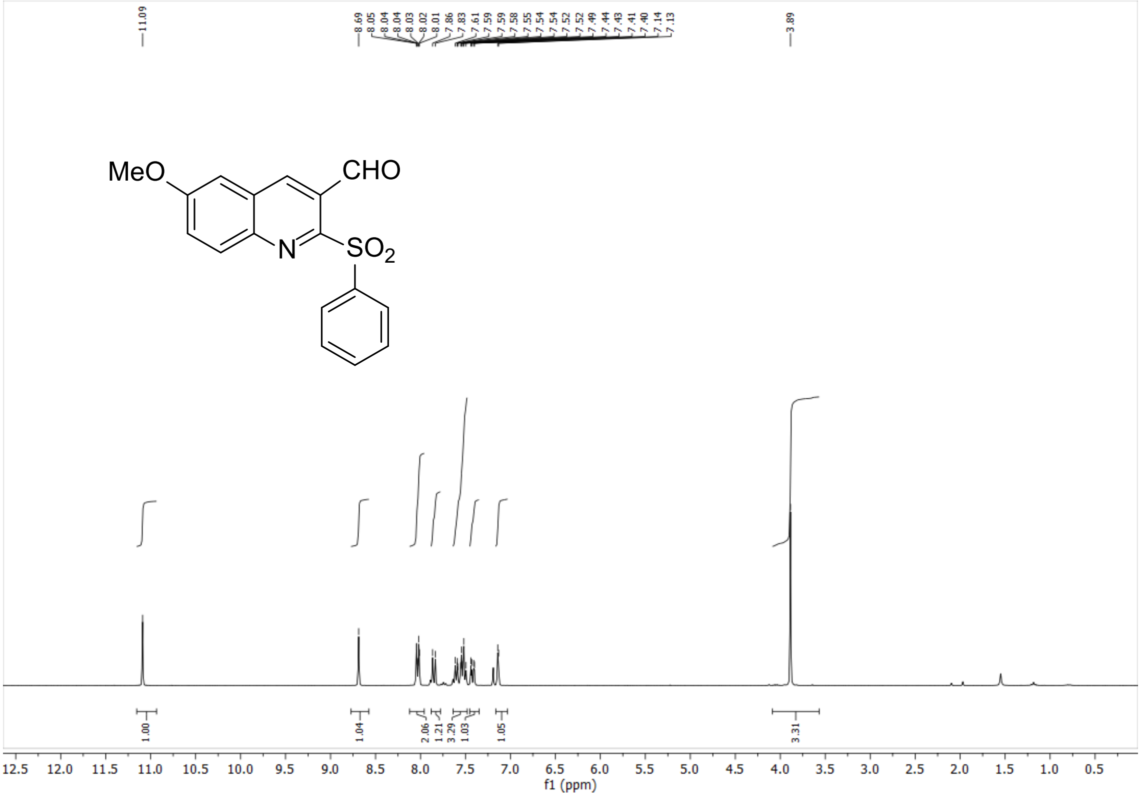

7 ^{1}H NMR

Following the **General procedure A**, the reaction of mixture **1**+**7** (200 mg, 0.611 mmol) with *N*-benzylhydroxylamine hydrochloride (146 mg, 0.917 mmol), Na_2SO_4 (260 mg, 1.835 mmol) and TEA (170 µL, 1.223 mmol) in THF (4 mL), for 1 h and work-up, after purification on column chromatography (hexane/AcOEt, 2:1) gave the expected nitrone **QN8** as a light yellow solid (97 mg, 42%): mp 164-6 °C; ^{1}H NMR (300 MHz, $CDCl_3$) δ 10.29 (s, 1H), 8.89 (s, 1H), 7.87 (dd, *J*= 8.4, 1.4 Hz, 1H), 7.69-7.25 (m, 11H), 7.04 (d, *J*=

2.7 Hz, 1H), 5.12 (s, 2H), 3.82 (s, 3H); ^{13}C NMR (75 MHz, $CDCl_3$) δ 160.5 (C), 152.5 (C), 141.6 (C), 139.1 (C), 136.3 (CH), 134.1 (C), 133.3 (CH), 131.6 (CH), 130.3 (C), 130.0 (2 CH), 129.7 (2 CH), 129.5 (2 CH), 129.4 (2 CH), 129.2 (CH), 128.4 (CH) 125.3 (CH), 121.4 (C), 106.1 (CH), 73.0 (CH_2), 56.1 (CH_3); MS (EI): 432.1 (3) [M^+], 291.1 (100) [M^+-SO_2Ph]. HRMS ESI_ACN. Calcd. for $C_{24}H_{20}N_2O_4S$: 432.11438. Found: 432.11454. Anal. Calcd. for $C_{24}H_{20}N_2O_4S$: C, 66.65; H, 4.66; N, 6.48; S, 7.41. Found: C, 66.88; H, 4.88; N, 6.74; S, 7.26.

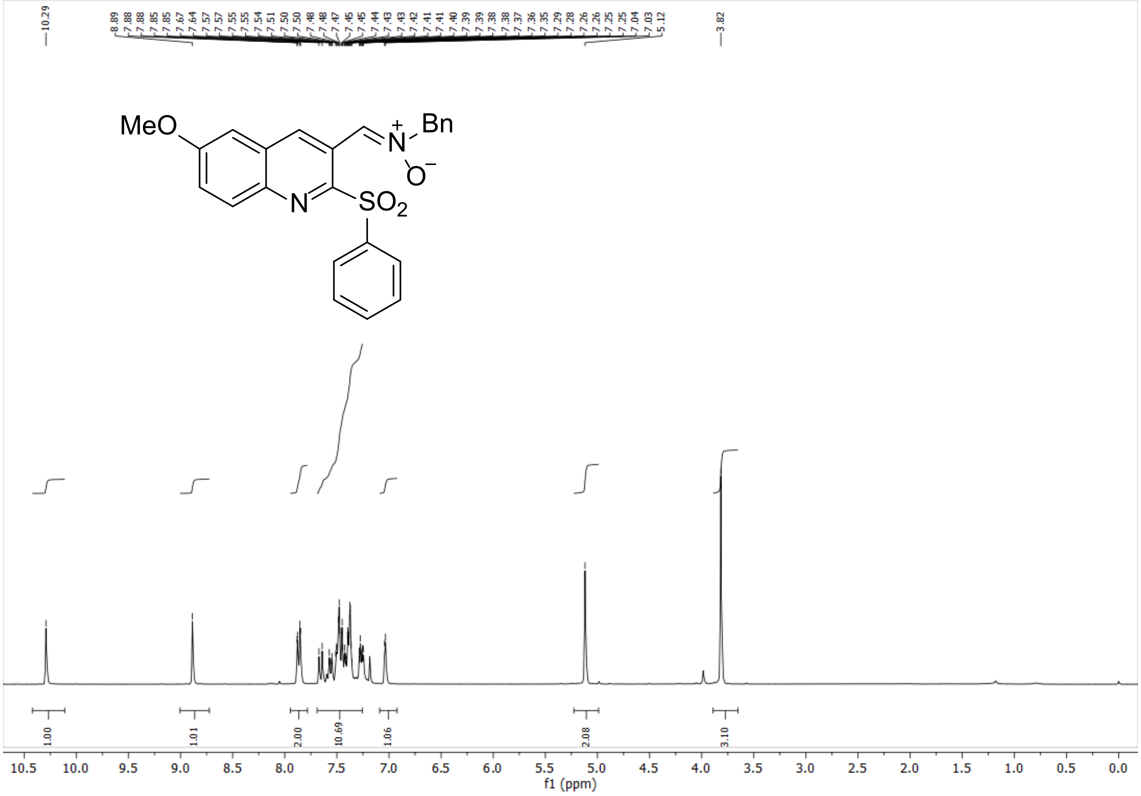

QN8 ^{1}H NMR

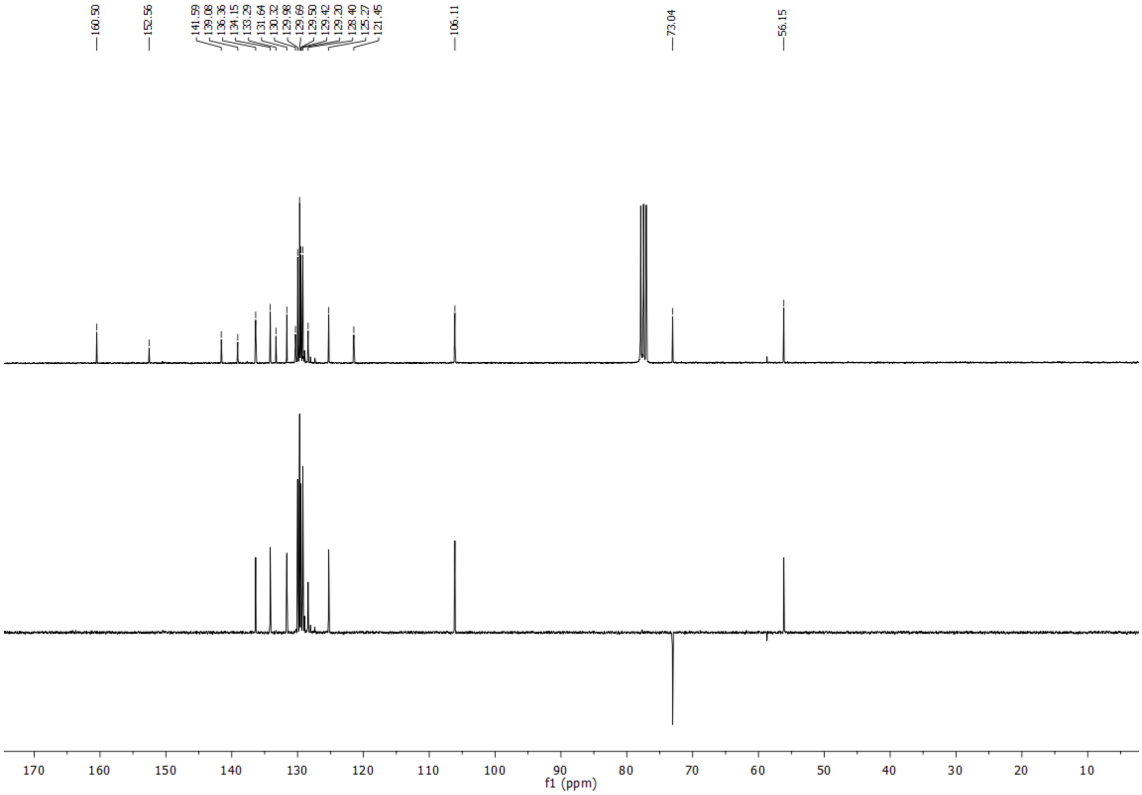

QN8 ^{13}C NMR vs DEPT

(*Z*)-*N*-*tert*-Butyl-1-(6-methoxy-2-(phenylsulfonyl)quinolin-3-yl)methanimine oxide (QN9). Following the **General procedure A**, the reaction of mixture **1**+**7** (70 mg, 0.214 mmol) with *N*-*tert*-butylhydroxylamine hydrochloride (90 mg, 0.321 mmol), Na_2SO_4 (180 mg, 0.642 mmol) and TEA (126 µL, 0.428 mmol) in THF (5 mL), for 6 h and work-up, after column chromatography (hexane/AcOEt, 2:1) gave nitrone **QN9** obtained as a light yellow solid (80 mg, 87%): mp 137-8 °C; ^{1}H NMR (300 MHz, $CDCl_3$) δ 10.39 (s, 1H), 9.04 (s, 1H), 8.00-7.90 (m, 2H), 7.70-7.41 (m, 4H), 7.27 (dd, *J*= 9.2, 2.7 Hz, 1H), 7.07 (d, *J*= 2.7 Hz, 1H), 3.83 (s, 3H), 1.62 (s, 9H); ^{13}C NMR (75 MHz, $CDCl_3$) δ 160.4 (C), 152.9 (C), 141.4 (C), 139.2 (C), 136.0 (CH), 134.1 (CH), 131.5 (CH), 130.5 (C), 129.6 (2 CH), 129.1 (2 CH), 124.9 (CH), 124.3 (CH), 122.0 (C), 106.1 (CH), 73.1 (C), 28.7 (3 x CH_3); MS (EI): 398.1 (3) [M^+], 257.1 (27) [M^+-SO_2Ph]. HRMS ESI_ACN. Calcd. for $C_{21}H_{22}N_2O_4S$: 398.13003. Found: 398.13047. Anal. Calcd. for $C_{21}H_{22}N_2O_4S$: C, 62.36; H, 5.65; N, 6.93; S, 7.93 Found: C, 62.49; H, 5.54; N, 6.75; S, 7.84.

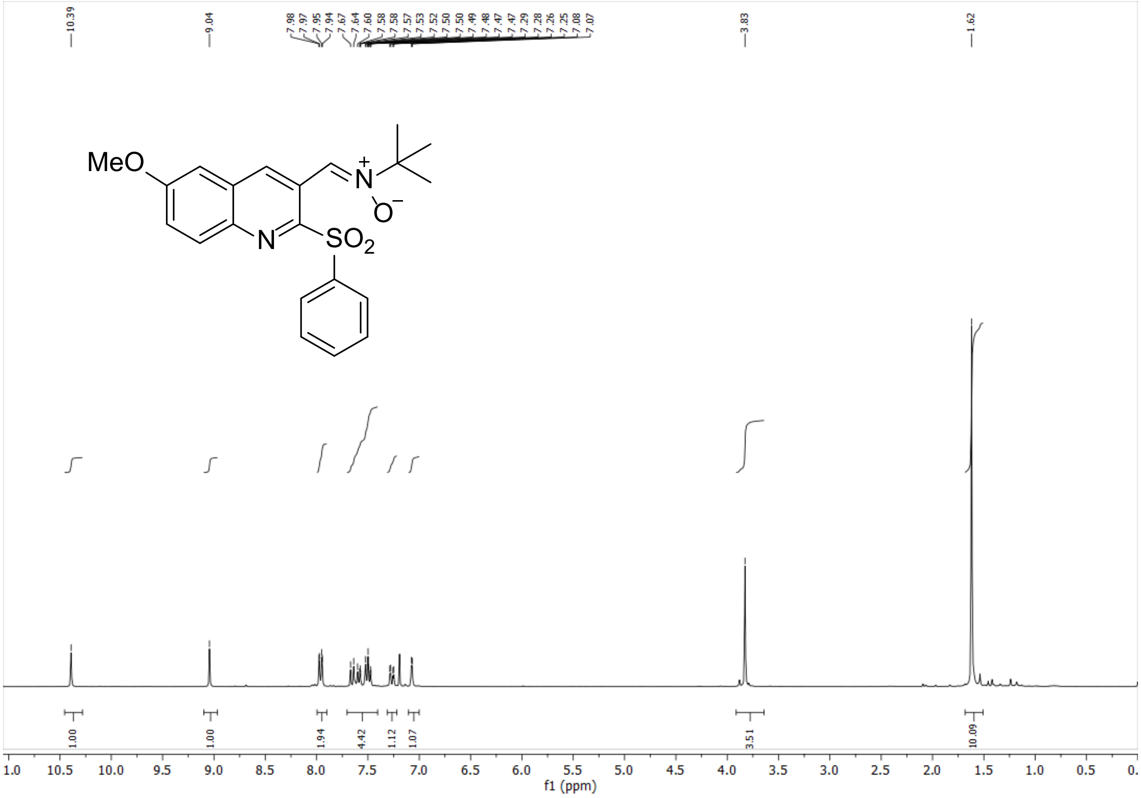

QN9 ^{1}H NMR

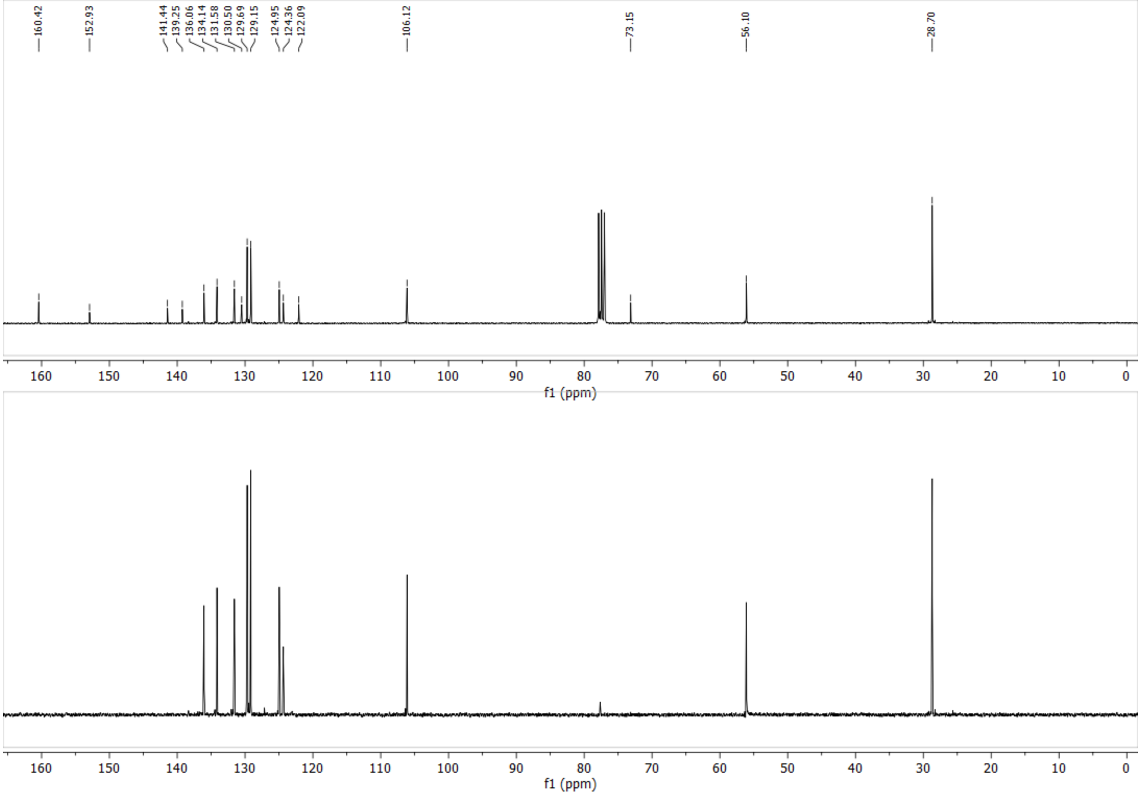

QN9 ^{13}C NMR vs DEPT

(*Z*)-*N*-Benzyl-1-(2-((4-nitrophenyl)sulfonyl)-6-methoxyquinolin-3-yl)methanimine oxide (QN10). Following the **General procedure B**, the reaction of 2-chloro-6-methoxyquinoline-3-carbaldehyde (**1**) (200 mg, 0.904 mmol) with 4-nitrobenzenesulfonyl chloride (239 mg, 1.085 mmol) and Na_2SO_3 (148 mg, 1.175 mmol) in H_2O (3 mL), after purification on column chromatography (hexane/AcOEt, 4:1), afforded 6-methoxy-2-((4-nitrophenyl)sulfonyl)quinoline-3-carbaldehyde (**8**) (178 mg, 53%, yield estimated on the ^{1}H NMR basis spectrum), a thick yellow gum, as a mixture of non-separable compounds **8** and **1** [^{1}H NMR (300 MHz, $CDCl_3$) (for compound **8**) δ 10.55 (d, *J*= 0.7 Hz), 8.64 (d, *J*= 0.7 Hz, 1H), 8.42 (d, *J*= 8.7 Hz, 2H), 8.30 (d, *J*= 8.7 Hz, 2H), 7.96 (dd, *J*= 9.3, 0.7 Hz, 1H), 7.52 (dd, *J*= 9.3, 2.8Hz, 1H), 7.19 (d, *J*= 2.8 Hz, 1H)].

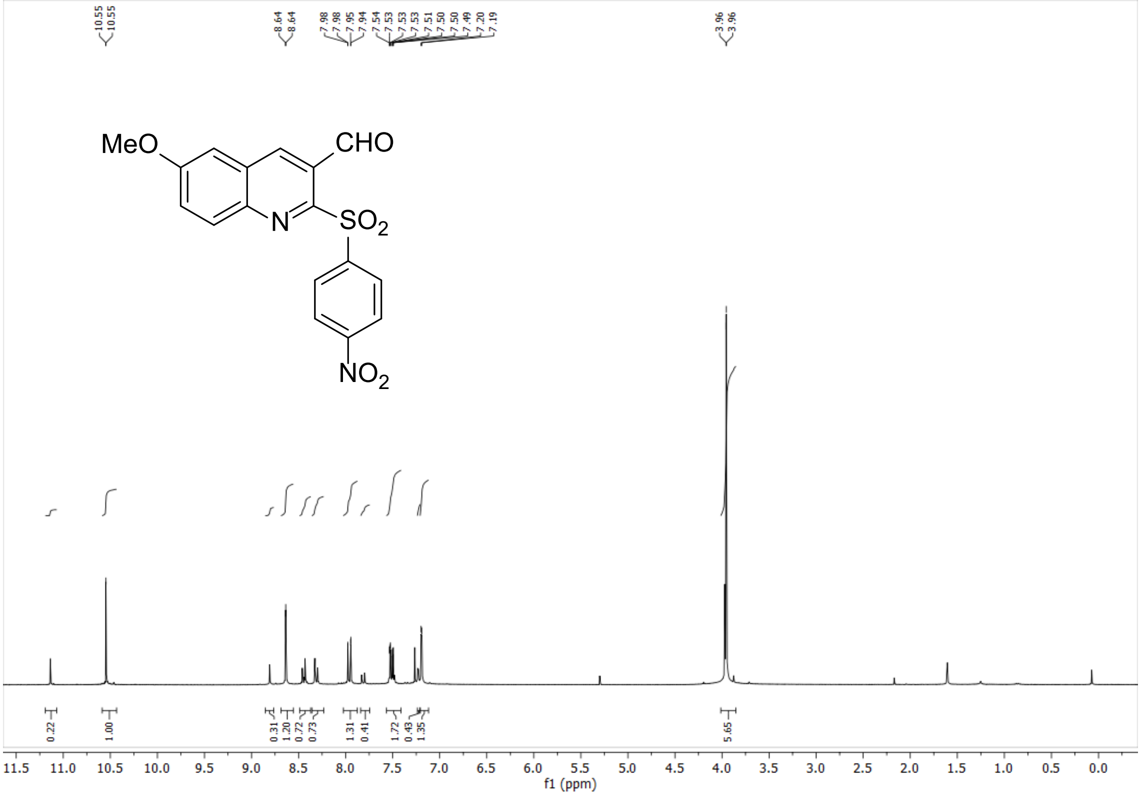

8 ^{1}H NMR

Following the **General procedure A**, the reaction of mixture **1**+**8** (90 mg, 0.410 mmol) with *N*-benzylhydroxylamine hydrochloride (98 mg, 0.615 mmol), Na_2SO_4 (116 mg, 0.821 mmol) and TEA (170 μL, 1.231 mmol) in THF (3 mL), for 1 h, after work-up and purification on column chromatography (DCM/AcOEt, 5:1), gave nitrone **QN10** as a yellow solid (80 mg, 92%): mp 175-7 °C; ^{1}H NMR (300 MHz, $CDCl_3$) δ 10.31 (s, 1H), 8.79 (s, 1H), 8.30 (d, *J*= 9.0 Hz, 2H), 8.10 (d, *J*= 9.0 Hz, 2H), 7.62-7.22 (m, 7H), 7.06 (d,

J= 2.0 Hz, 1H), 5.14 (s, 2H), 3.83 (s, 3H); ^{13}C NMR (75 MHz, $CDCl_3$) δ 160.7 (C), 151.7 (C), 151.2 (C), 144.7 (C), 141.2 (C), 136.4 (CH), 133.1 (C), 131.4 (2 CH), 131.3 (C), 130.4 (2 CH), 129.9 (CH), 129.8 (CH), 129.5 (2 CH), 127.5 (CH), 125.6 (CH), 124.1 (2 CH), 121.3 (C), 106.1 (CH), 73.1 (CH_2), 56.2 (CH_3); MS (EI): 477.1 (1) [M^+], 291.1 (100) [M^+-SO_2Ar]. HRMS ESI_ACN. Calcd. for $C_{24}H_{19}N_3O_6S$: 477.09946. Found: 477.09948. Anal. Calcd. for $C_{24}H_{19}N_3O_6S$: C, 59.25; H, 4.14; N, 8.64; S, 6.59. Found: C, 59.19; H, 4.12; N, 8.74; S, 6.54.

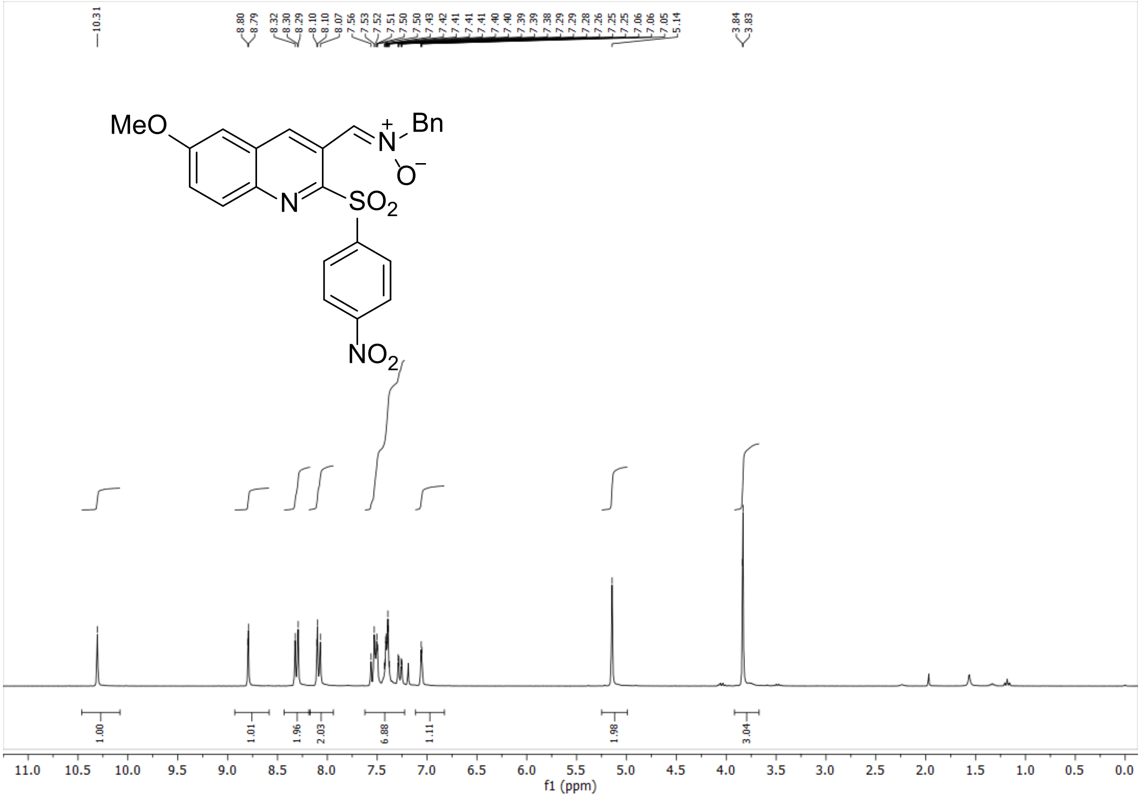

QN10 ^{1}H NMR

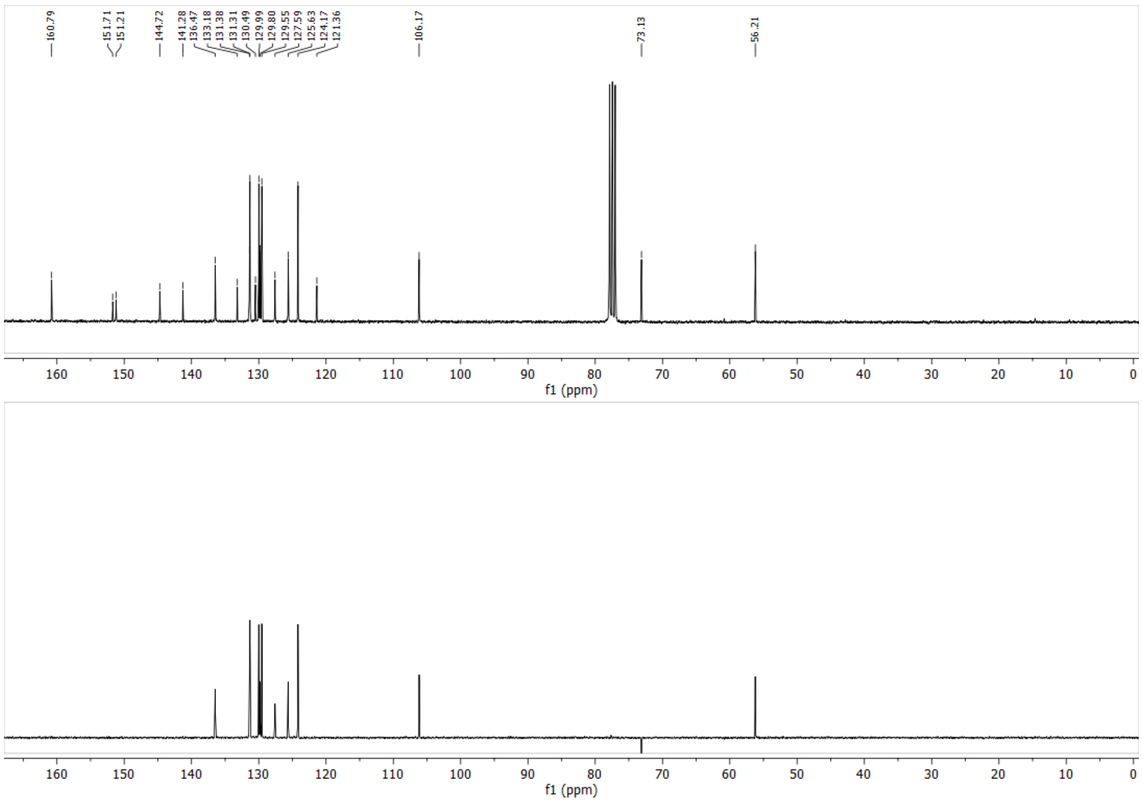

QN10 ^{13}C NMR vs DEPT

(*Z*)-*N*-*tert*-Butyl-1-(2-((4-nitrophenyl)sulfonyl)-6-methoxyquinolin-3-yl)methanimine oxide (QN11). Following **the General procedure A**, the reaction of mixture **1**+**8** (116 mg, 0.525 mmol), with *N*-*tert*-butylhydroxylamine hydrochloride (98 mg, 0.615 mmol), Na_2SO_4 (98 mg, 0.791 mmol) and TEA (220 µL, 1.550 mmol) in THF (5 mL), for 6 h, after work-up and purification on column chromatography (DCM/AcOEt, 10:1), gave nitrone **QN11** as a yellow solid (51 mg, 22%): mp 144-5 °C; ^{1}H NMR (300 MHz, $CDCl_3$) δ 10.41 (s, 1H), 8.99 (s, 1H), 8.36 (d, *J*= 8.9 Hz, 2H), 8.18 (d, *J*= 8.9 Hz, 2H), 7.52 (d, *J*= 9.2 Hz, 1H), 7.26 (dd, *J*= 9.2, 2.8 Hz, 1H), 7.09 (d, *J*= 2.8 Hz, 1H), 3.84 (s, 3H), 1.64 (s, 9H); ^{13}C NMR (75 MHz, $CDCl_3$) δ 160.6 (C), 152.2 (C), 151.2 (C), 144.8 (C), 141.1 (C), 136.1 (CH), 131.4 (2 CH), 131.2 (CH), 130.6 (C), 125.2 (CH), 124.1 (2 CH), 123.5 (CH), 121.9 (C), 106.2 (CH), 73.3 (C), 56.1 (CH_3), 28.7 (3 x CH_3); MS (EI): 443.1 (3) [M^+], 257.1 (32) [M^+-SO_2Ar]. HRMS ESI_ACN. Calcd. for $C_{21}H_{21}N_3O_6S$: 443.11511. Found: 443.11544. Anal. Calcd. for $C_{21}H_{21}N_3O_6S$: C, 56.88; H, 4.77; N, 9.48; S, 7.23 Found: C, 56.68; H, 4.88; N, 9.41; S, 7.06.

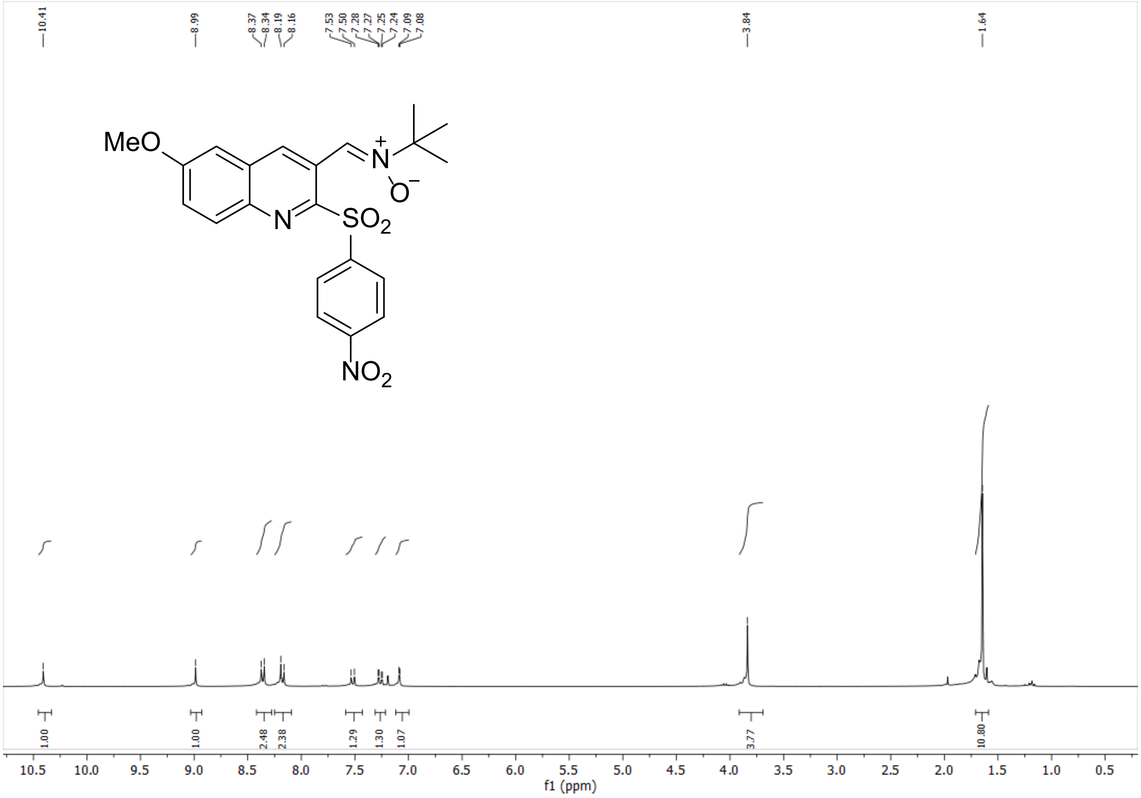

QN11 ^{1}H NMR

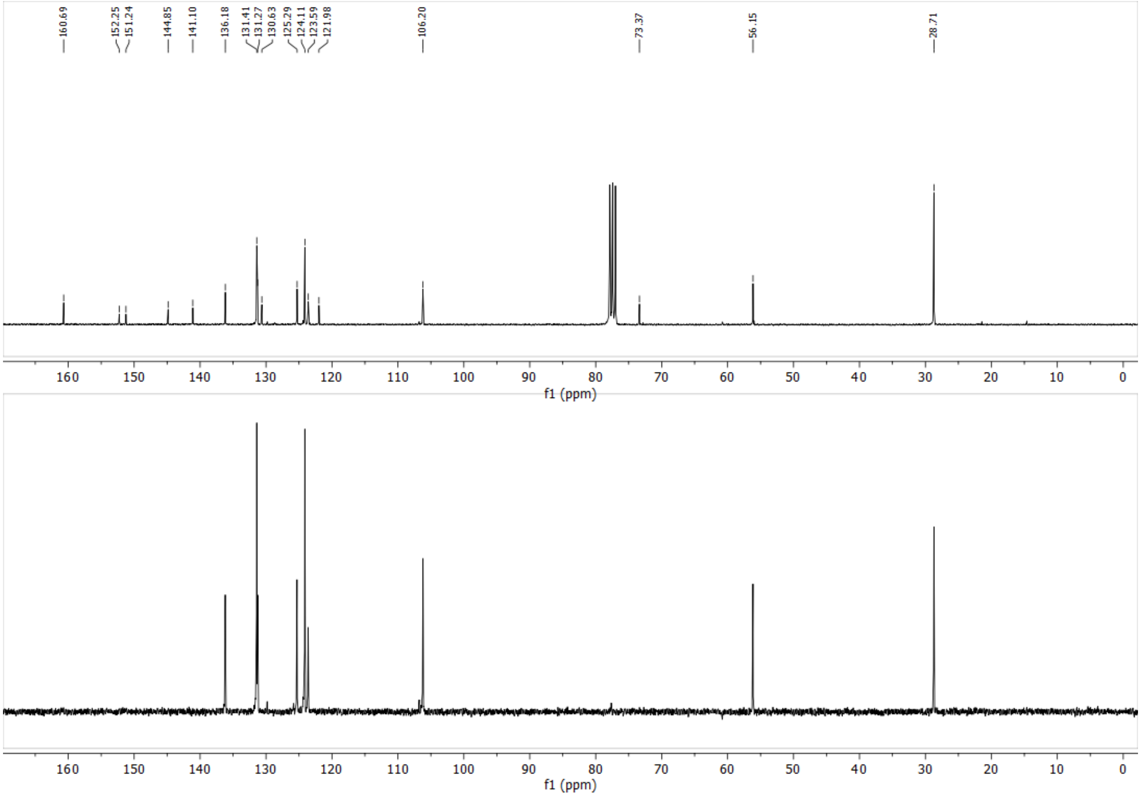

QN11 ^{13}C NMR vs DEPT

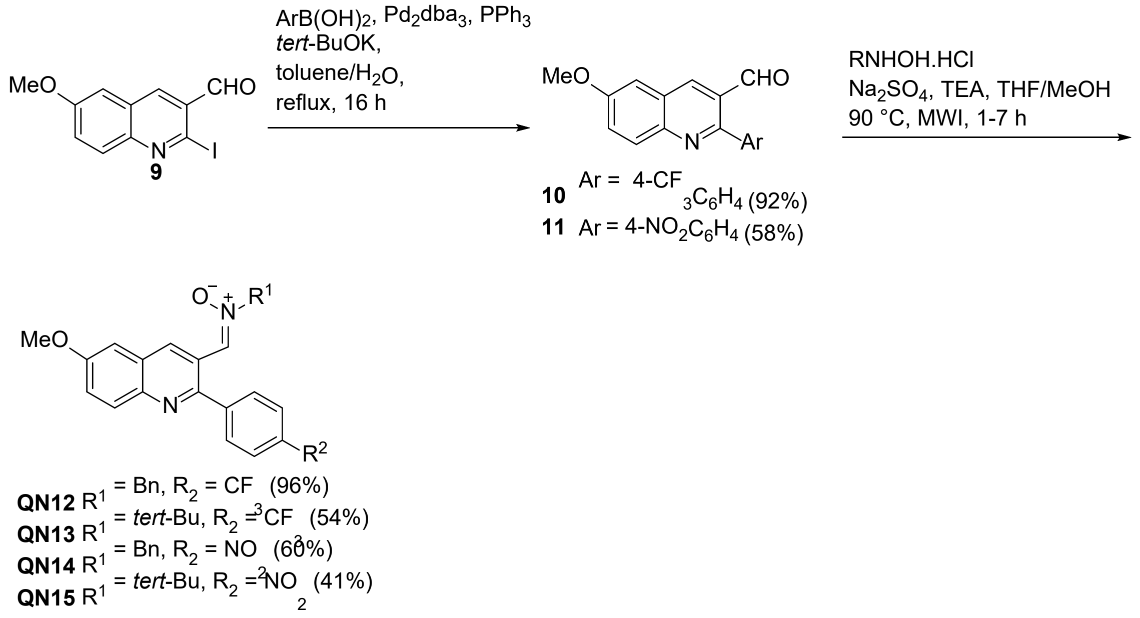

Scheme 6. Synthesis of 2-arylquinolin nitrones **QNs 12-15**.

6-Methoxy-2-(4-trifluoromethylphenyl)quinoline-3-carbaldehyde (10). Following the **General procedure C**, the reaction of commercial 2-iodo-6-methoxyquinoline-3-carbaldehyde (**9**) (50 mg, 0.159 mmol), *p*-trifluoromethylphenylboronic acid (36 mg, 0.191 mmol), *tert*-BuOK (18 mg, 0.159 mmol), Pd_2dba_3 (7 mg, 0.007 mmol) and PPh_3 (2 mg, 0.007 mmol) in toluene/H_2O (4+1 mL), after purification on column chromatography (hexane/AcOEt, 5:1), gave compound **10** (48 mg, 92%) was obtained as a yellow solid: ^1H NMR (300 MHz, $CDCl_3$) δ 10.09 (s, 1H), 8.68 (s, 1H), 8.03 (d, *J*= 9.2 Hz, 1H), 7.74 (s, 4H), 7.48 (dd, *J* = 9.2, 2.8 Hz, 1H), 7.19 (s, 1H), 3.92 (s, 3H). HRMS ESI_ACN Calcd. for $C_{18}H_{12}NF_3O_2$: 331.08201. Found: 331.08222.

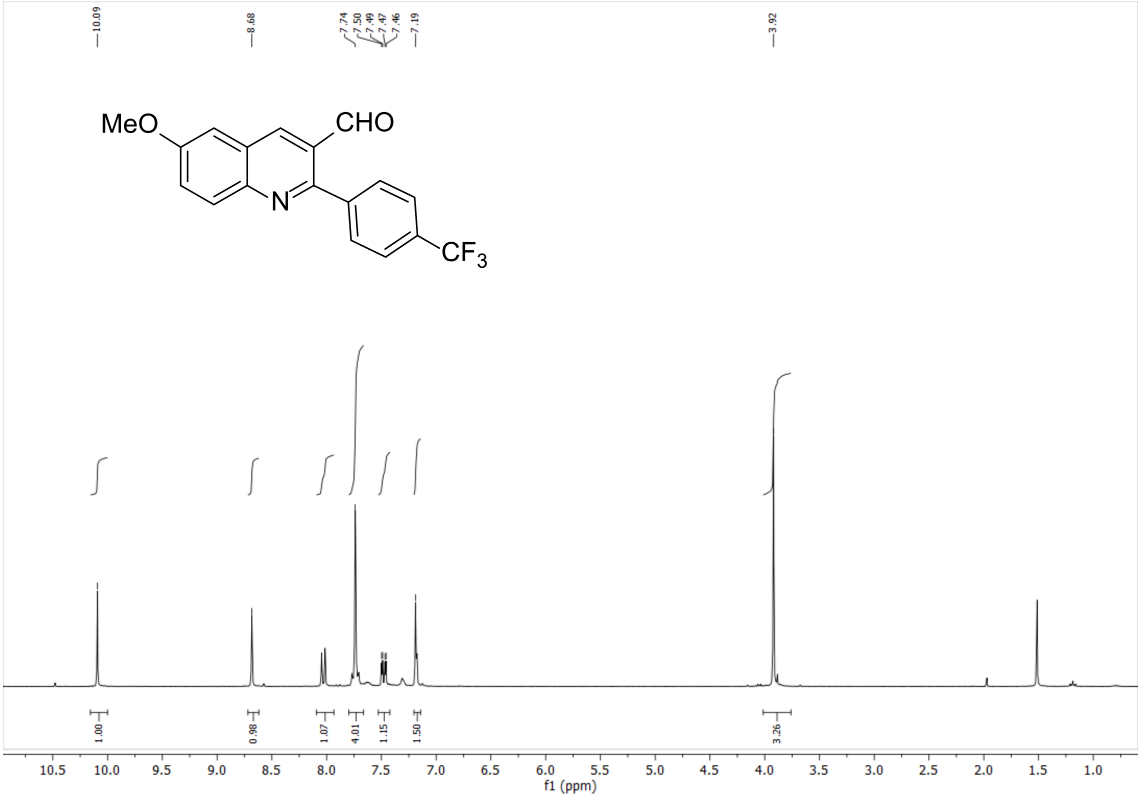

10 ^{1}H NMR

(*Z*)-*N*-Benzyl-1-(6-methoxy-2-(4-(trifluoromethyl)phenyl)quinolin-3-yl)methanimine oxide (QN12). Following the **General procedure A**, the reaction of compound **10** (100 mg, 0.302 mmol) with *N*-benzylhydroxylamine hydrochloride (71 mg, 0.453 mmol), Na_2SO_4 (86 mg, 0.604 mmol) and TEA (120 μL, 0.906 mmol) in THF (3 mL), for 1 h, after purification on column chromatography (DCM/AcOEt, 10:1), gave nitrone **QN12** as a yellow solid (120 mg, 96%): mp 234-5 °C; ^{1}H NMR (300 MHz, $CDCl_3$) δ 10.15 (s, 1H), 7.88 (d, *J*= 9.2 Hz, 1H), 7.56 (d, *J*= 8.0 Hz, 2H), 7.44 (d, *J*= 8.0 Hz, 2H), 7.37-7.22 (m, 6H), 7.19 (d, *J*= 4.8 Hz, 1H), 7.13 (d, *J*= 2.7 Hz, 1H), 4.95 (s, 2H), 3.87 (s, 3H); ^{13}C NMR (75 MHz, $CDCl_3$) δ 158.8 (C), 155.0 (C), 143.9 (C), 143.9 (C), 143.25 (C), 134.7 (CH), 132.8 (C), 131.3 (CH), 131.0 (CH), 130.2 (2 CH), 130.0 (2 CH), 129.8 (2 CH), 129.5 (2 CH), 128.5 (C), 125.93, 125.88, 125.83, 125.78 (C, q, *J*= 15.0 Hz), 124.6 (CH), 122.4 (C), 106.4 (CH), 72.2 (CH_2), 56.0 (CH_3); MS (EI): 436.1 (29) [M^+]. HRMS ESI_ACN. Calcd. for $C_{25}H_{19}F_3N_2O_2$: 436.13986. Found: 436.13835. Anal. Calcd. for $C_{25}H_{19}F_3N_2O_2$: C, 67.87; H, 4.48; N, 6.33. Found: C, 67.90; H, 4.65; N, 6.52.

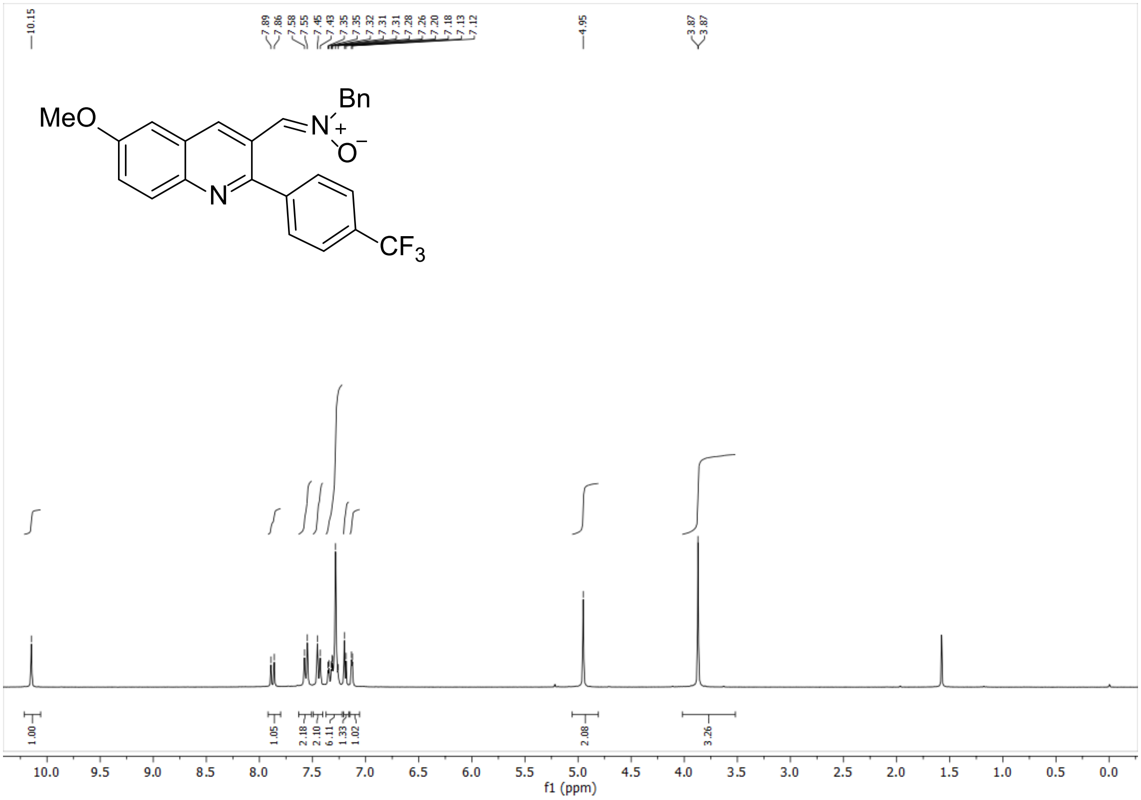

QN12 ^{1}H NMR

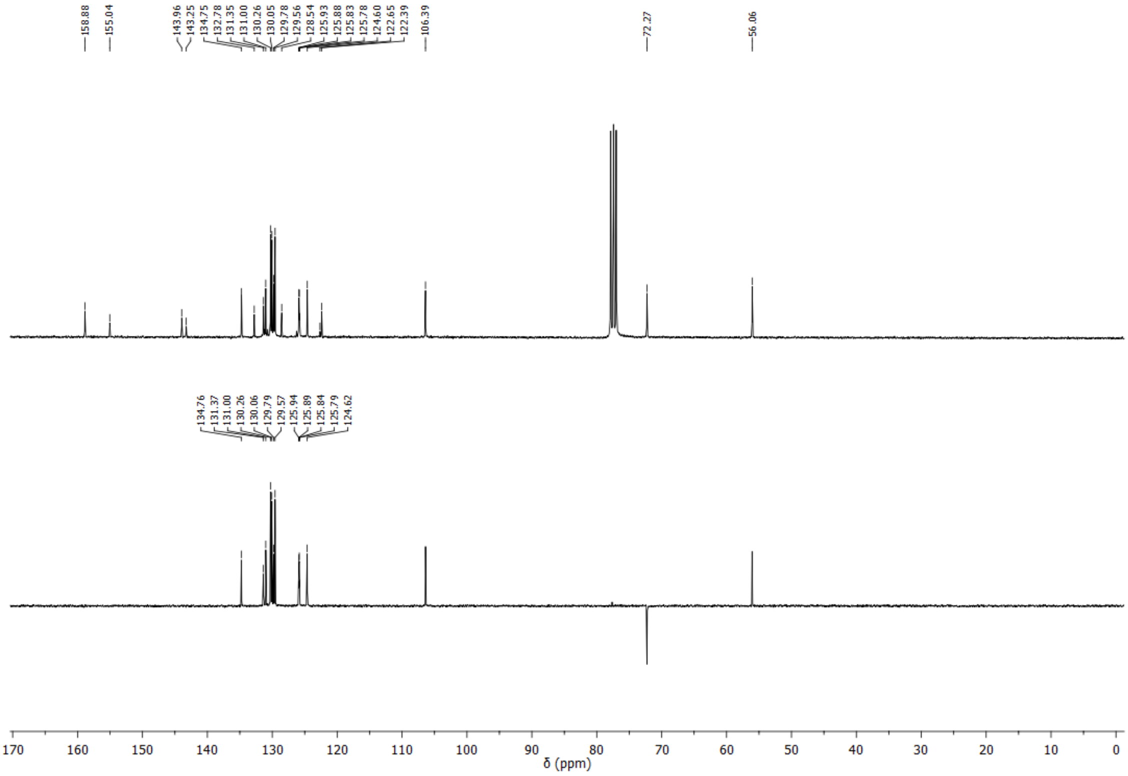

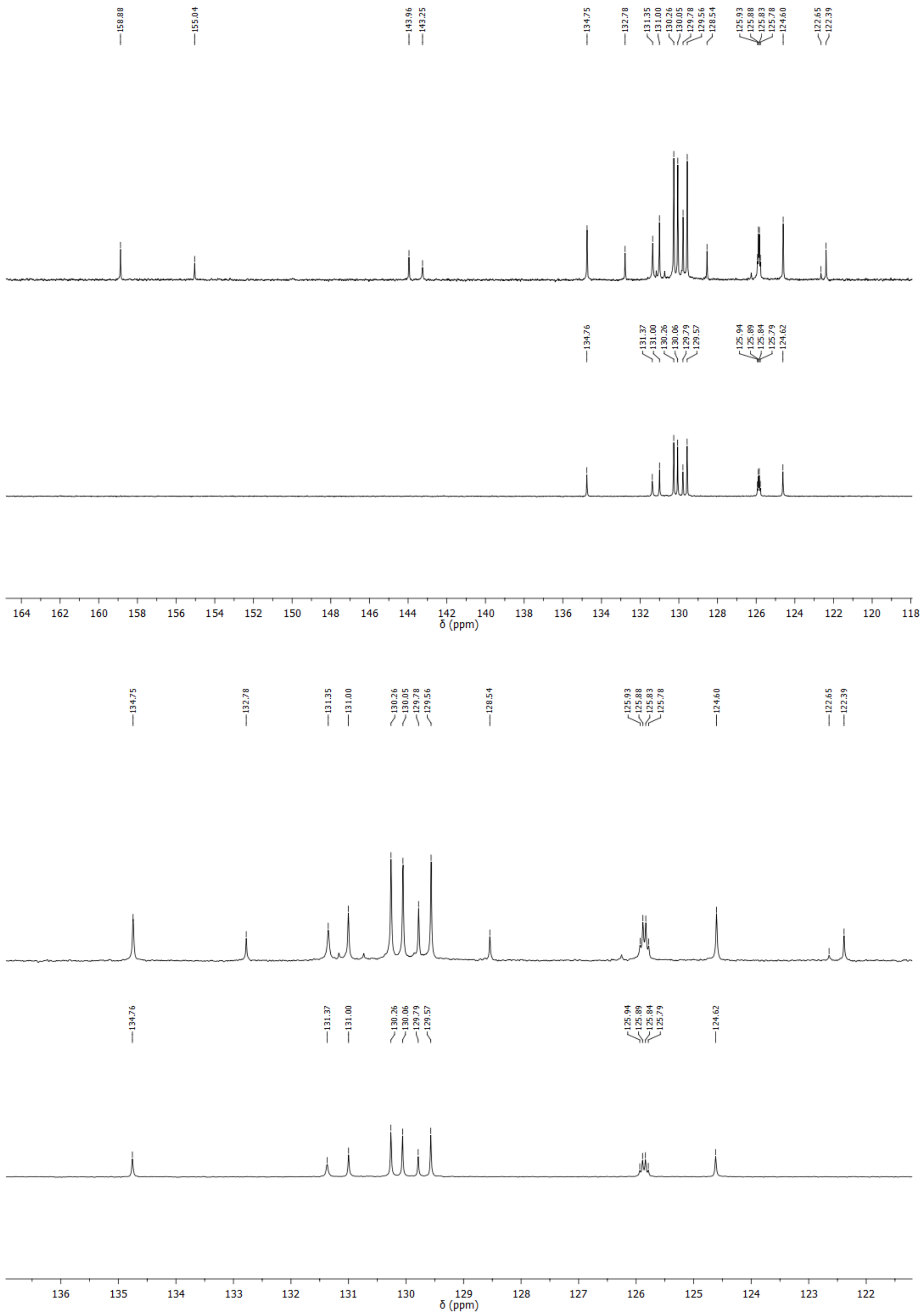

QN12 ^{13}C NMR vs DEPT

(*Z*)-*N-tert*-Butyl-1-(6-methoxy-2-(4-(trifluoromethyl)phenyl)quinolin-3-yl)methanimine oxide (QN13). Following the **General procedure A**, the reaction of compound **10** (120 mg, 0.363 mmol) with *N-tert*-butylhydroxylamine hydrochloride (68 mg, 0.544 mmol), Na_2SO_4 (103 mg, 0.726 mmol) and TEA (150 µL, 1.089 mmol) in THF (3 mL), for 6 h, after purification on column chromatography (DCM/AcOEt, 7:1), gave nitrone **QN13** as a yellow solid (78 mg, 54%): mp 192-4 °C; ^{1}H NMR (300 MHz, $CDCl_3$) δ 10.25 (s, 1H), 7.91 (ddd, *J*= 9.2, 2.8, 1.2 Hz, 1H), 7.72 (d, *J*= 9.1 Hz, 2H), 7.65 (d, *J*= 9.1 Hz, 2H), 7.59 (s,1H), 7.34 (dd, *J* = 9.2, 2.8 Hz, 1H), 7.14 (dd, *J* = 2.8, 1.2 Hz, 1H), 3.87 (s, 3H), 1.45 (s, 9H); ^{13}C NMR (75 MHz, $CDCl_3$) δ 158.8 (C), 155.3 (C), 143.8 (3 C), 134.7 (CH), 131.0 (CH), 130.9 (2 CH), 130.4 (2 CH), 128.6 (C), 127.0 (CH), 126.7 (q, *J* = 270.5 Hz, C), 125.98, 125.93, 125.88, 125.82 (C, q, *J*= 15.0 Hz), 124.3 (CH), 122.9 (C), 106.4 (CH), 72.3 (C), 56.0 (CH_3), 28.6 (3 x CH_3). HRMS ESI_ACN. Calcd. for $C_{22}H_{21}F_3N_2O_2$: 402.15551. Found: 402.15507. Anal. Calcd. for $C_{22}H_{21}F_3N_2O_2$: C, 64.94; H, 5.33; N, 6.90. Found: C, 64.77; H, 5.32; N, 7.17.

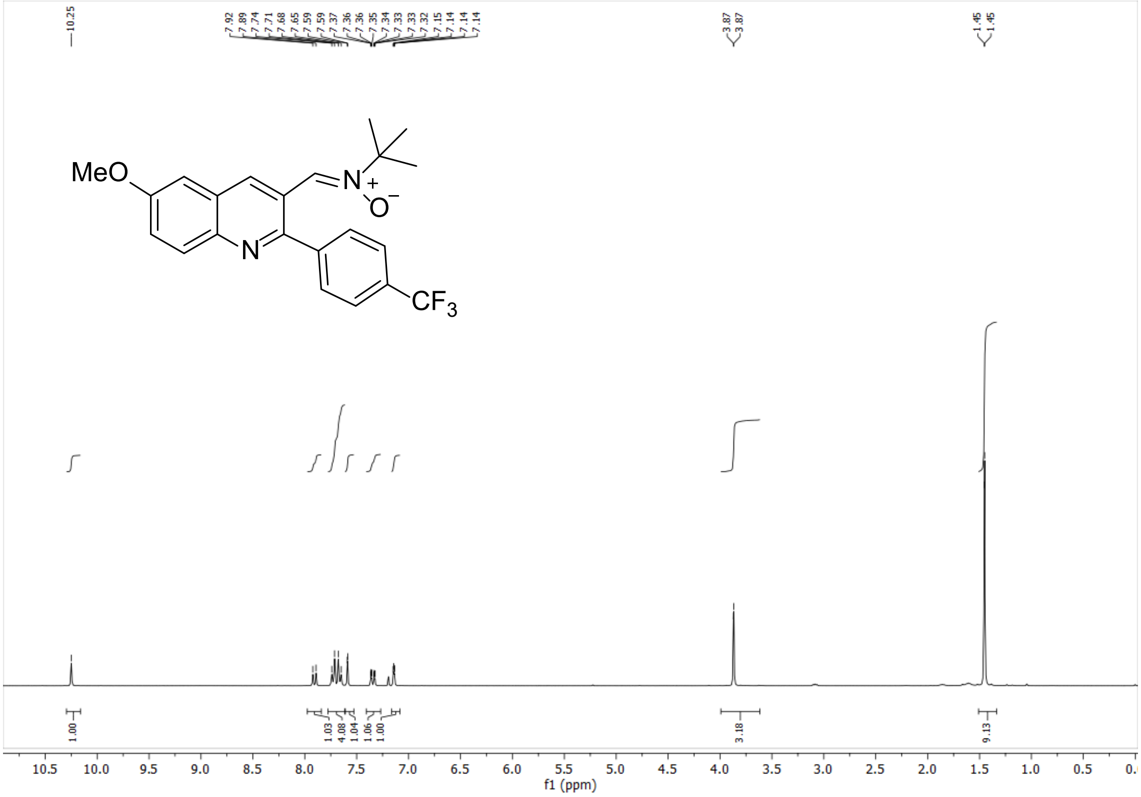

QN13 ^{1}H NMR

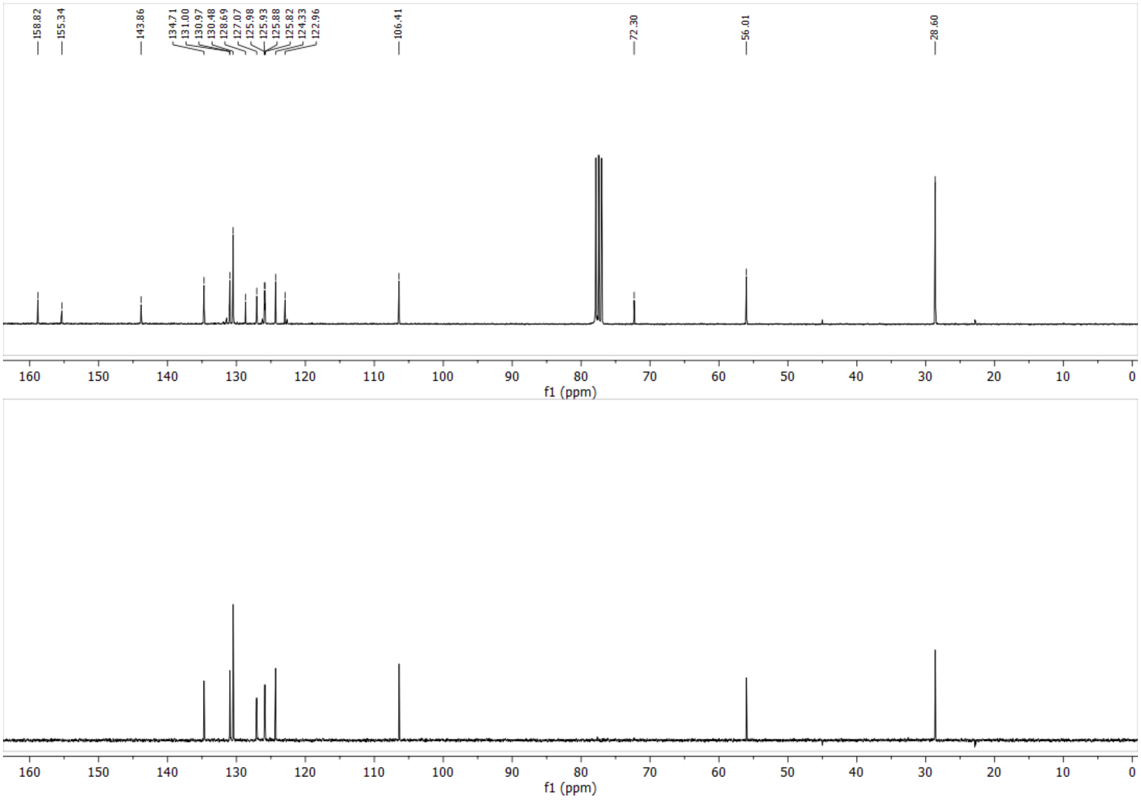

QN13 ^{13}C NMR vs DEPT

6-Methoxy-2-(4-nitrophenyl)quinoline-3-carbaldehyde (11). Following the **General procedure C**, the reaction of compound **9** (100 mg, 0.452 mmol), *p*-nitrophenylboronic acid (90 mg, 0.542 mmol), *tert*-BuOK (50 mg, 0.452 mmol), Pd_2dba_3 (21 mg, 0.022 mmol) and PPh_3 (18 mg, 0.068 mmol) in toluene/H_2O (4+1 mL), after purification on column chromatography (hexane/AcOEt, 8:2), gave a mixture of **9**+**11** [^{1}H NMR (300 MHz, $CDCl_3$) (major compound **11**) [δ 10.17 (s, 1H), 8.77 (s, 1H), 8.42 (dd, *J*= 9.0, 2.3 Hz, 2H), 8.11 (d, *J*= 9.2 Hz, 1H), 7.86 (dd, *J*= 9.0, 2.3 Hz, 2H), 7.57 (dd, *J*= 9.2, 1.9 Hz, 1H), 7.26 (m, 1H), 4.00 (s, 3H)] (81 mg, 58%, yield estimated by ^{1}H NMR in the reaction mixture) was obtained as a non-separable mixture with starting material. The mixture of compounds was submitted to the next step without further purification.

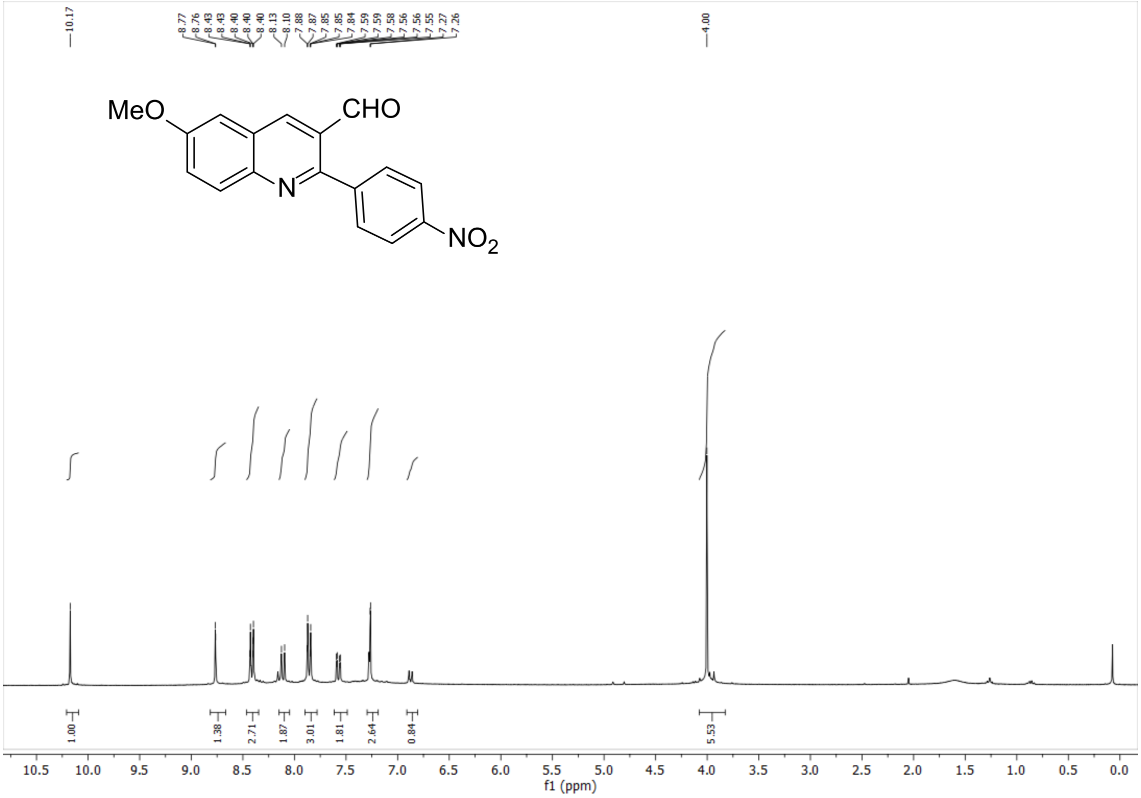

9+11 ^{1}H NMR

(*Z*)-*N*-Benzyl-1-(6-methoxy-2-(4-nitrophenyl)quinolin-3-yl)methanimine oxide (QN14). Following the **General procedure A**, the reaction of mixture **9+11** (100 mg, 0.325 mmol) with *N*-benzylhydroxylamine hydrochloride (77 mg, 0.487 mmol), Na_2SO_4 (138 mg, 0.975 mmol) and TEA (90 µL, 0.650 mmol) in THF (3.2 mL), for 1 h, after purification on column chromatography (DCM/AcOEt, 2:1), gave nitrone **QN14** as a yellow solid (80 mg, 60%): mp 251-3 °C; ^{1}H NMR (300 MHz, $CDCl_3$) δ 10.13 (s, 1H), 8.15 (d, *J*= 8.7 Hz, 2H), 7.88 (d, *J*= 9.2 Hz, 1H), 7.51 (d, *J*= 8.7 Hz, 2H), 7.39-7.24 (m, 6H), 7.18-7.16 (m, 1H), 7.14-7.12 (m, 1H), 4.96 (s, 2H), 3.88 (s, 3H); ^{13}C NMR (75 MHz, $CDCl_3$) δ 159.1 (C), 153.9 (C), 148.0 (C), 146.0 (C), 143.9 (C), 134.8 (CH), 132.8 (C), 131.0 (2 CH), 130.9 (2 CH), 130.0 (CH), 129.8 (CH), 129.7 (2 CH), 129.6 (CH), 128.7 (C), 124.8 (CH), 124.0 (2 CH), 122.3 (C), 106.3 (CH), 72.3 (CH_2), 56.1 (CH_3); MS (EI): 413.1 (23) [M^+], 291.1 (100) [M^+-$C_6H_4NO_2$]. HRMS ESI_ACN. Calcd. for $C_{24}H_{19}N_3O_4$: 413.13756. Found: 413.12755. Anal. Calcd. for $C_{24}H_{19}N_3O_4$: C, 64.55; H, 4.42; N, 9.22. Found: C, 64.50; H, 4.33; N, 8.99.

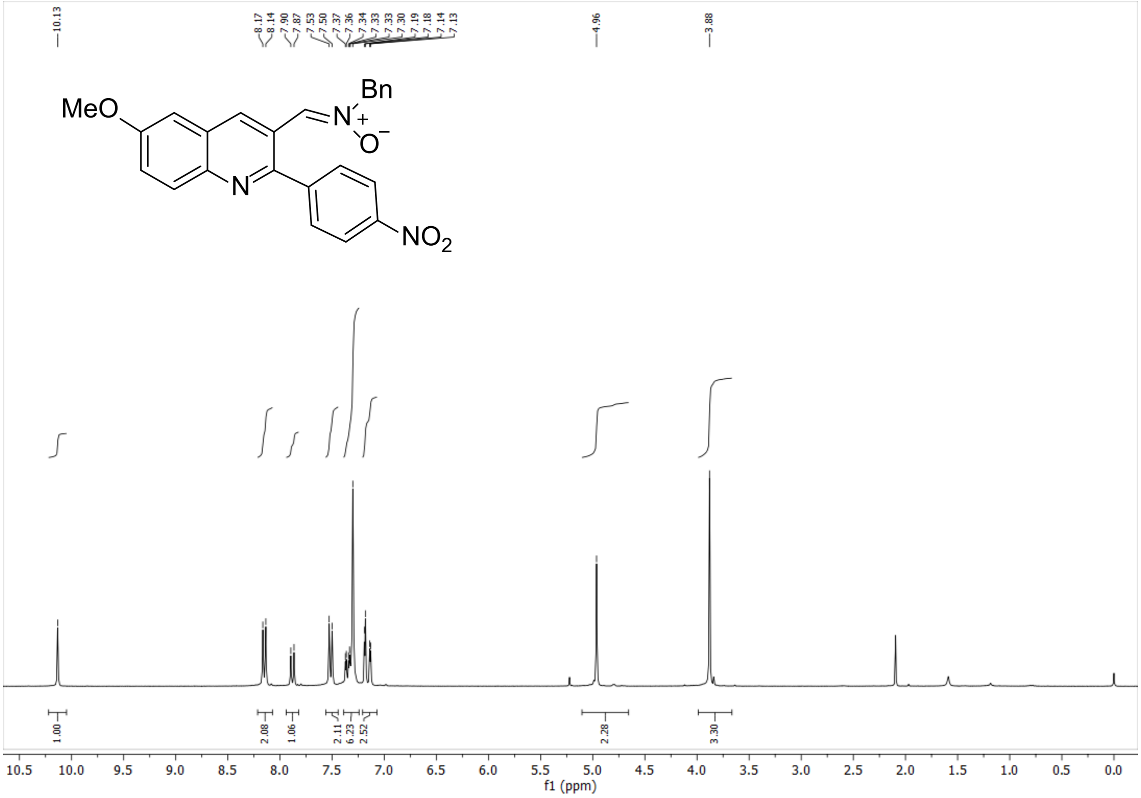

QN14 ^{1}H NMR

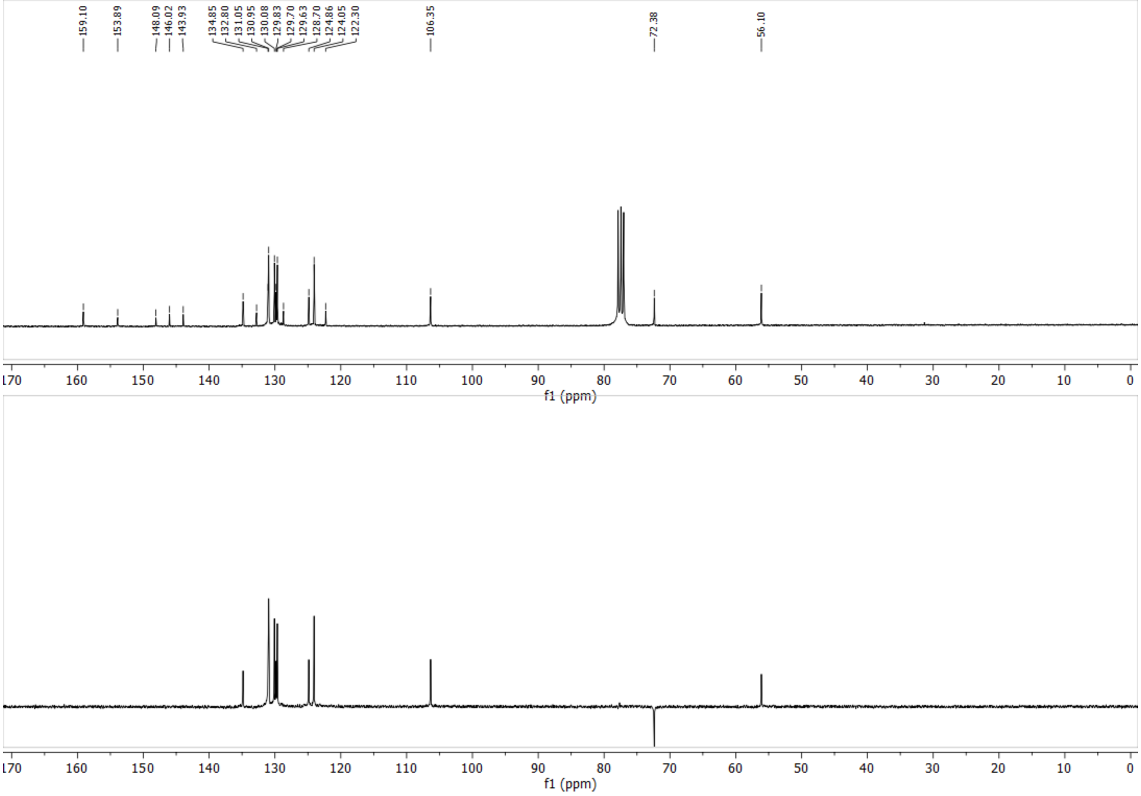

QN14 ^{13}C NMR vs DEPT

(*Z*)-*N*-*tert*-Butyl-1-(6-methoxy-2-(4-nitrophenyl)quinolin-3-yl)methanimine oxide (QN15). Following the **General procedure A**, the reaction of mixture **9**+**11** (100 mg, 0.325 mmol) with *N*-*tert*-butylhydroxylamine hydrochloride (61 mg, 0.487 mmol), Na_2SO_4 (138 mg, 0.975 mmol) and TEA (90 µL, 0.650 mmol) in THF (3.2 mL), for 6 h; after purification on column chromatography (DCM/AcOEt, 4:1), gave nitrone **QN15**, as a yellow solid (45 mg, 41%): mp 189-191°C; ^{1}H NMR (300 MHz, $CDCl_3$) δ 10.23 (s, 1H), 8.32 (d, *J*= 8.8 Hz, 2H), 7.91 (d, *J*= 9.2 Hz, 1H), 7.74 (d, *J*= 8.8 Hz, 2H), 7.56 (s, 1H), 7.36 (dd, *J*= 9.2, 2.8 Hz, 1H), 7.14 (d, *J*= 2.8 Hz, 1H), 3.87 (s, 3H), 1.46 (s, 9H); ^{13}C NMR (75 MHz, $CDCl_3$) δ 159.0 (C), 154.2 (C), 148.3 (C), 146.6 (C), 143.8 (C), 134.8 (CH), 131.1 (2 CH), 131.0 (CH), 128.8 (C), 126.6 (CH), 124.6 (CH), 124.1 (2 CH), 122.9 (C), 106.3 (CH), 72.4 (C), 56.0 (CH_3), 28.6 (3 x CH_3); MS (EI): 379.1 (38) [M^+], 322.1 (80) [M^+-*tert*-Bu]. HRMS ESI_ACN. Calcd. for $C_{21}H_{21}N_3O_4$: 379.15321. Found: 379.11535. Anal. Calcd. for $C_{21}H_{21}N_3O_4$: C, 66.48; H, 5.58; N, 11.08. Found C, 66.34; H, 5.46; N, 10.37.

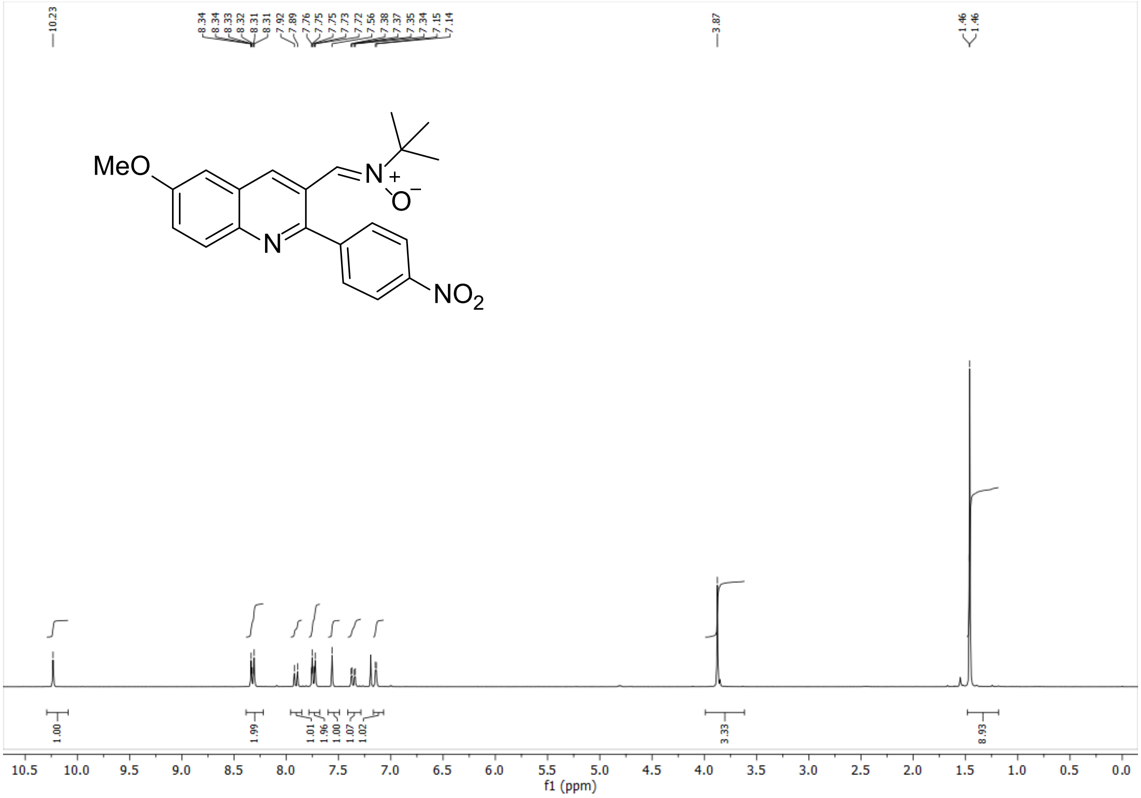

QN15 ^{1}H NMR

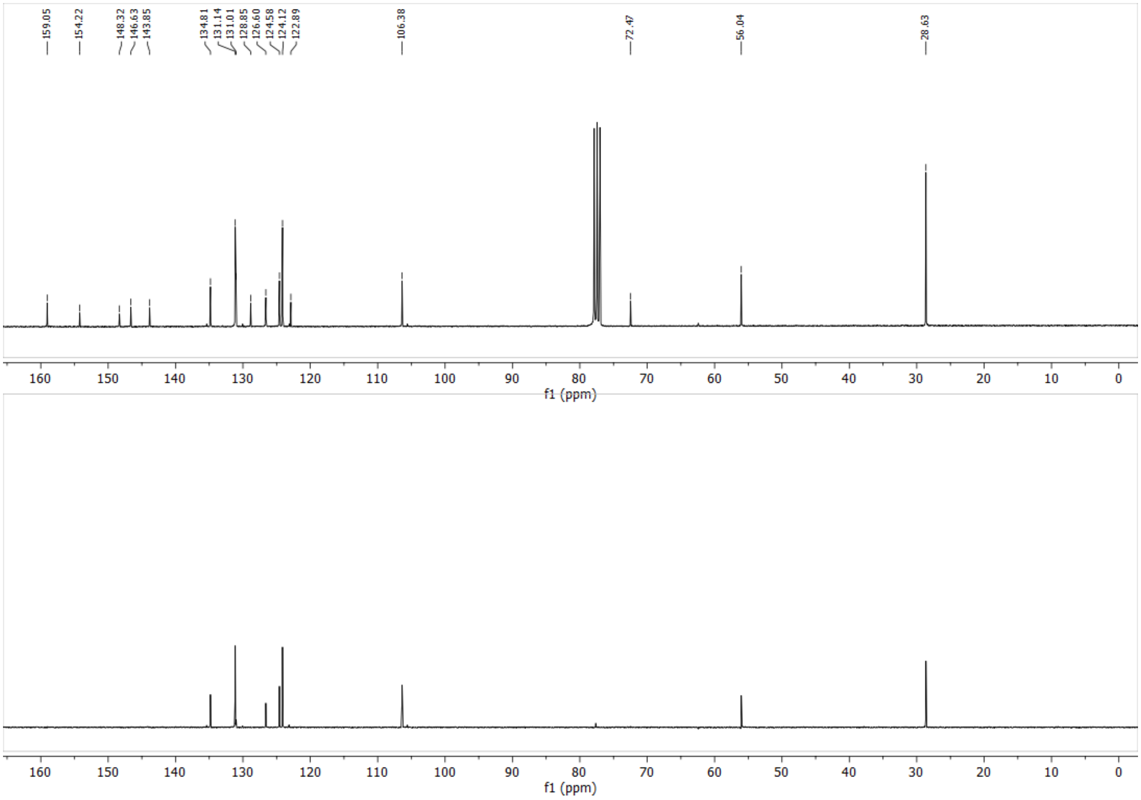

QN15 ^{13}C NMR vs DEPT

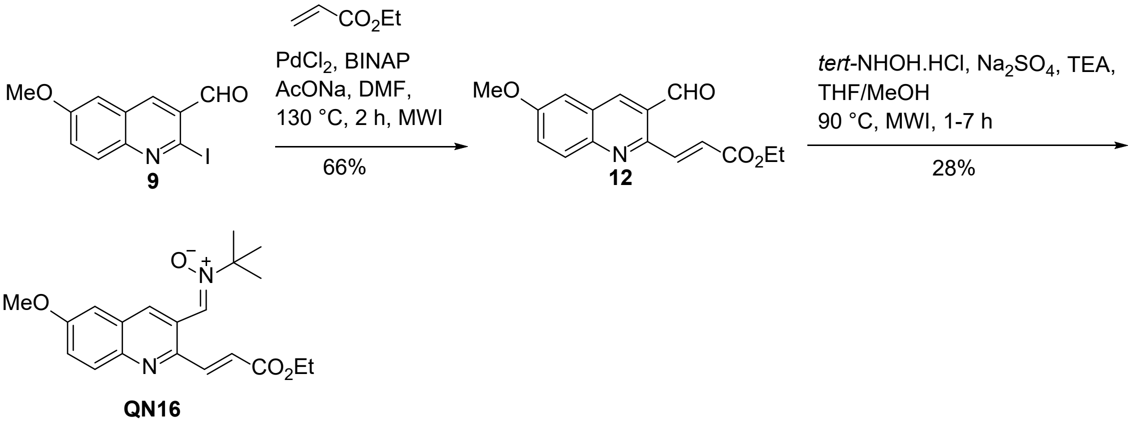

Scheme 7. Synthesis of 2-ethoxyoxopropenyl quinoline nitrone **QN16**.

Ethyl (*E*)-3-(3-formyl-6-methoxyquinolin-2-yl)acrylate (12). A solution of 2-iodoquinoline **9** (50 mg, 0.159 mmol), ethyl acrylate (34 mL, 0.318 mmol), $PdCl_2$ (3 mg, 5 mol%), BINAP (10 mg, 5 mol%), and AcONa (36 mg, 0.318 mmol) in DMF (1 mL) was heated at 130 ^{0}C for 2 h under MWI. After that time, the solvent was evaporated under reduced pressure, and the mixture purified on column chromatography

(hexane/AcOEt, 10:1) to yield pure product **12** (30 mg, 66%) as a yellow thick gum: ^{1}H NMR (300 MHz, $CDCl_3$) δ 10.40 (d, *J* = 1.2 Hz, 1H), 8.60 (dd, *J*= 15.3, 1.2 Hz, 1H), 8.49 (s, 1H), 8.00 (d, *J*= 9.3 Hz, 1H), 7.49 (dd, *J*= 9.3, 2.4 Hz, 1H), 7.22 (d, *J*= 15.3 Hz, 1H) 7.16 (d, *J*= 2.4 Hz, 1H), 4.31 (q, *J*= 6.9 Hz, 2H), 3.95 (s, 3H), 1.36 (t, *J*= 6.9 Hz, 3H); ^{13}C NMR (75 MHz, $CDCl_3$) δ 191.1 (C), 166.9 (C), 159.5 (C), 149.6 (C), 146.1 (C), 142.0 (CH), 139.3 (CH), 131.8 (CH), 128.8 (C), 128.0 (C), 126.7 (CH), 126.2 (CH), 106.1 (CH), 61.1 (CH_2), 56.1 (CH_3), 14.7 (CH_3); MS (EI): 285.1 (31) [M^+]. HRMS ESI_ACN. Calcd. for $C_{16}H_{15}NO_4$: 285.10011. Found: 285.10143.

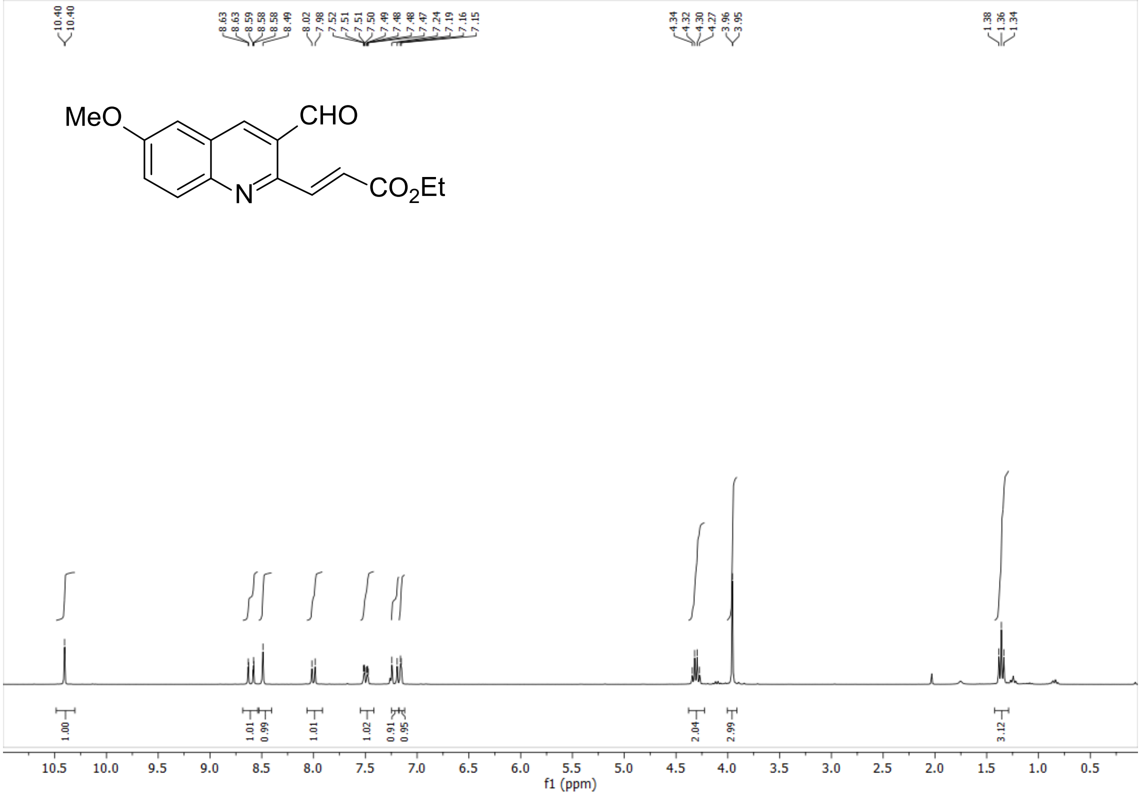

12 ^{1}H NMR

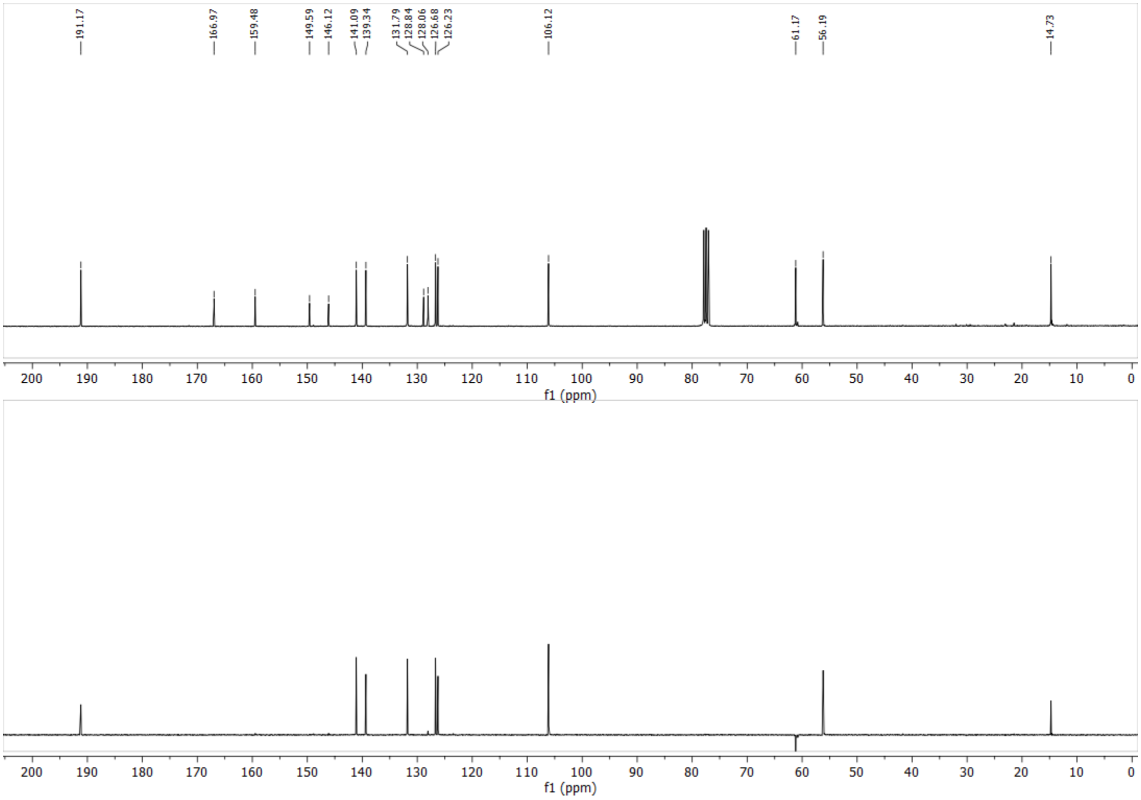

12^{13}C NMR vs DEPT

(*Z*)-*N*-*tert*-Butyl-1-(2-((E)-3-ethoxy-3-oxoprop-1-en-1-yl)-6-methoxyquinolin-3-yl)methanimine oxide (QN16). Following the **General procedure A**, the reaction of compound **12** (80 mg, 0.280 mmol) with *N*-*tert*-butylhydroxylamine hydrochloride (52 mg, 0.421 mmol), Na_2SO_4 (119 mg, 0.843 mmol) and TEA (78 µL, 0.562 mmol) in THF (2 mL) for 6 h, after work-up and purification on column chromatography (DCM/AcOEt, 7:3), gave nitrone **QN16** as a yellow solid (26 mg, 26%); mp 152-4 °C; ^{1}H NMR (300 MHz, $CDCl_3$) δ 10.05 (s, 1H), 7.98 (s, 1H), 7.95 (d, *J*= 15.4 Hz, 1H), 7.86 (d, *J*= 9.2 Hz, 1H), 7.31 (dd, *J*= 9.2, 2.8 Hz, 1H), 7.07 (d, *J*= 15.4 Hz, 1H), 7.06 (d, *J*= 7.4 Hz, 1H), 4.24 (q, *J*= 7.1 Hz, 2H), 3.84 (s, 3H), 1.62 (s, 9H), 1.29 (t, *J*= 7.1 Hz, 3H); ^{13}C NMR (75 MHz, $CDCl_3$) δ 167.1 (C), 159.0 (C), 148.5 (C), 144.1 (C), 139.5 (CH), 134.3 (CH), 131.2 (CH), 129.4 (C), 125.8 (CH), 124.8 (CH), 124.5 (CH), 123.7 (C), 106.1 (CH), 72.7 (C), 61.2 (CH_2), 56.0 (CH_3), 28.7 (3 x CH_3), 14.7 (CH_3). HRMS ESI_ACN. Calcd. for $C_{20}H_{24}N_2O_4$: 356.17361. Found: 356.17531. Anal. Calcd. for $C_{20}H_{24}N_2O_4$: C, 67.40; H, 6.79; N, 7.86. Found: C, 67.28; H, 6.82; N, 7.88.

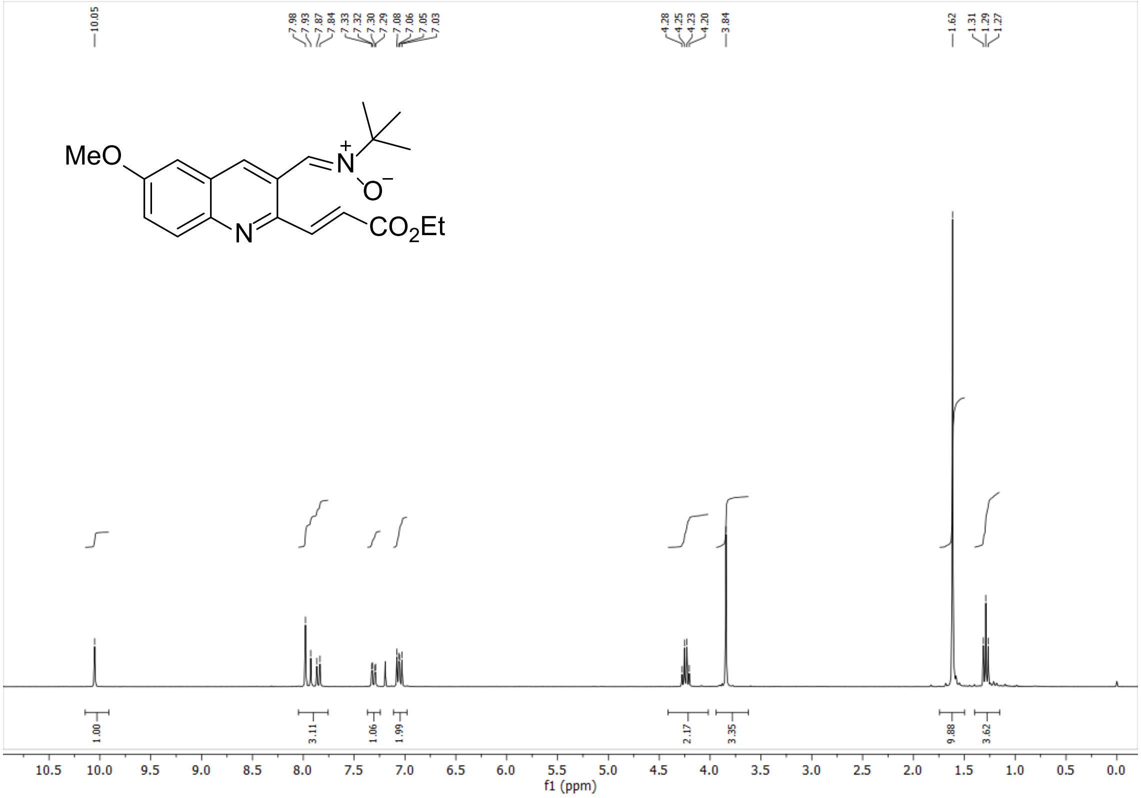

QN16 ^{1}H NMR

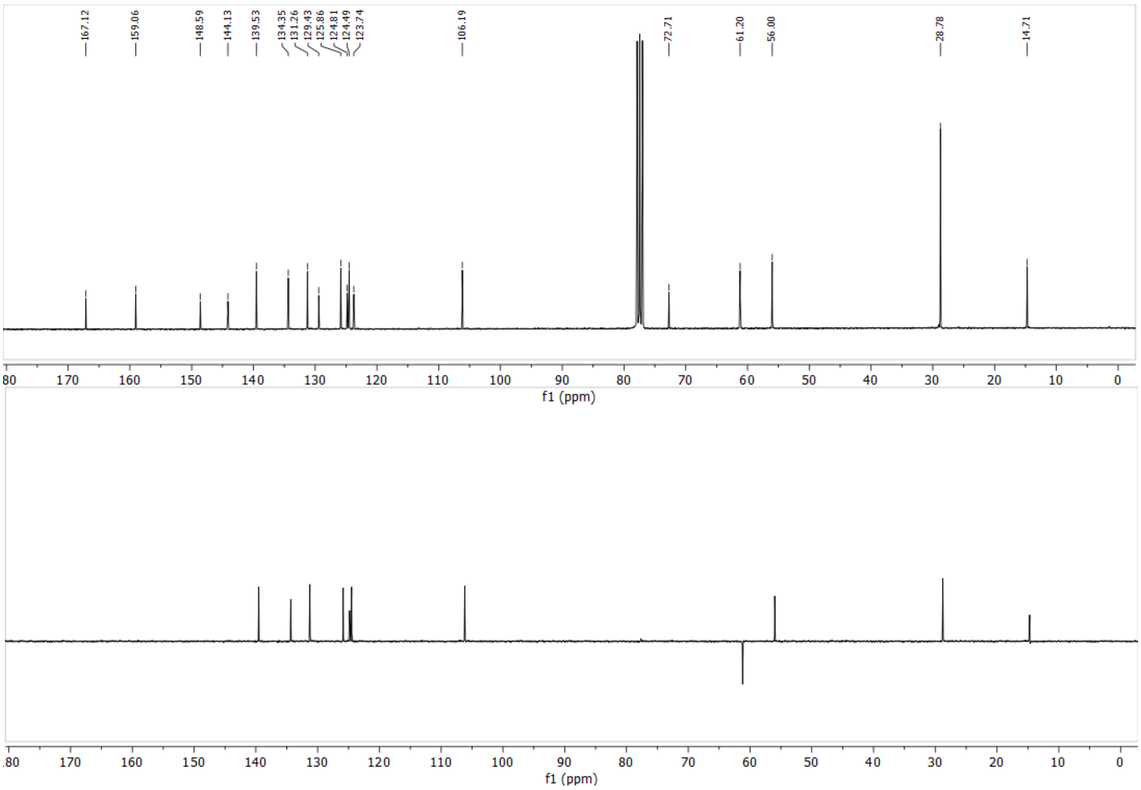

QN16 ^{13}C NMR vs DEPT

2. Neuroprotection studies of QNs 1-16

Table S1. Neuroprotective activity for **NXY-059**, **QN23** and **QNs 1-16** in neuronal cultures exposed to OGD.[a]

Compound	Concn (μM)	Neuroprotection (%)
**NXY-059**	100	41.93 ± 2.26
	250	51.26 ± 2.12
	500	45.64 ± 1.56
**QN23**	10	49.00 ± 0.89
	**100**	79.70 ± 0.98 **
	**250**	60.68 ± 1.29 **
	500	17.60 ± 0.77
**QN1**	0.1	19.81± 0.92
	1	26.89± 0.88
	10	34.81± 0.78
	100	41.26± 1.91
**QN2**	1	33.66± 0.73
	10	33.26± 0.93
	100	39.98± 0.77
	**250**	62.43± 0.59 **
**QN3**	0.1	30.05± 0.55
	1	45.23± 1.00
	10	20.59± 0.88
	20	< 0
**QN4**	0.01	15.30± 0.43
	0.1	40.38± 0.35
	**1**	67.89± 1.44 **
	**10**	64.93± 0.57 **
	100	36.67± 0.44
**QN5**	1	24.50± 0.54
	10	42.41± 0.98
	100	41.71± 0.62
	**250**	60.19 ± 1.08 *
**QN6**	1	26.27± 0.89
	10	25.08± 1.03
	100	24.81± 0.72
**QN7**	1	38.45± 0.62
	**10**	58.64± 1.11 *
	100	38.84± 1.10
	200	51.72± 0.99

**QN8**	0.1	25.21± 0.58
	1	54.11± 2.27
	10	54.69± 0.83
**QN9**	1	22.00 ± 1.05
	10	33.73 ± 3.19
	100	< 0
	250	< 0
**QN10**	0.05	30.62± 0.80
	0.1	41.14± 0.59
	1	47.25± 0.94
	10	< 0
**QN11**	0.1	27.23± 1.09
	1	46.50± 1.94
	10	14.91± 0.60
	20	1.14± 0.42
**QN12**	0.1	7.26± 0.16
	1	2.20± 0.05
	10	< 0
	20	< 0
**QN13**	1	39.29 ± 1.16
	10	5.95 ± 0.12
	100	< 0
	200	< 0
**QN14**	0.1	38.54± 0.70
	1	60.10± 1.24
	10	16.76± 0.55
	20	14.74± 0.10
**QN15**	0.1	32.42± 0.43
	1	48.09± 1.21
	10	46.81± 1.21
	**50**	70.26 ± 0.63 *
**QN16**	1	42.20 ± 0.47
	10	55.73 ± 1.65
	100	20.86
	250	< 0

[a] Neuroprotection was defined as the percentage to reach the control value, defined as 100%, from R24h value, defined as 0%. *P < 0.05 and **P < 0.01, compared with **NXY-059** (250 µM), by ANOVA and Dunnett's post-test. Statistical significances of the data lower than 250 µM **NXY-059** value were not shown.

In order to express cell viability as a more useful concept in our search for protective compounds in the ischemic disease, we defined neuroprotection activity as the effect that achieved a cellular viability higher than the vehicle-treated group (R24h), which set our basal neuroprotection value (0%). Cell viability observed in the control group was set as 100% of neuroprotection. The neuroprotection values obtained for standards **NXY-059**, **QN23**, and **QNs 1–16** have been gathered together in Table S1.
